# Supplementary material for: Synthesis and Antibacterial Evaluation of N-phenylacetamide Derivatives Containing 4-Arylthiazole Moieties
Source: Molecules. 2020 Apr 12;25(8):1772. doi: 10.3390/molecules25081772 (PMC7221908; doi:10.3390/molecules25081772)
Supplement: Supplementary file 1 [file molecules-25-01772-s001.pdf]

molecules

Supporting information

Synthesis and Antibacterial Evaluation of *N*-Phenylacetamide Derivatives Containing 4-Arylthiazole Moiety

Hui Lu, Xia Zhou*, Lei Wang, Linhong Jin*

State Key Laboratory Breeding Base of Green Pesticide and Agricultural Bioengineering, Key Laboratory of Green Pesticide and Agricultural Bioengineering, Ministry of Education, Guizhou University, Huaxi District, Guiyang 550025, China. luhui2624904231@163.com (H.L.); wanglei880328@163.com(L.W.);

* Correspondence: E-mail: linhong_j@126.com, zhouxia_j@126.com; Tel.: +86-851-3620-521 (X.Z. & L.J.); Fax: +868513622211 (X.Z. & L.J.)

^{1}H and ^{13}C NMR spectra of target compound **5-I**

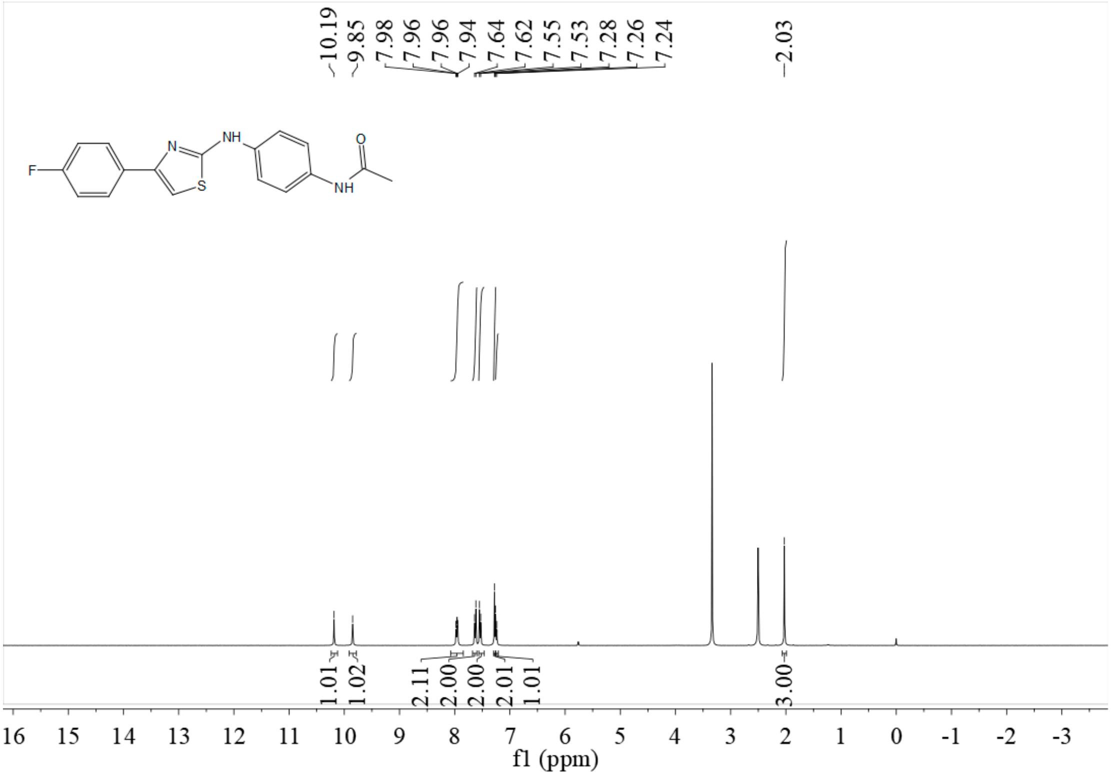

^{1}H NMR spectrum of compound **A**1

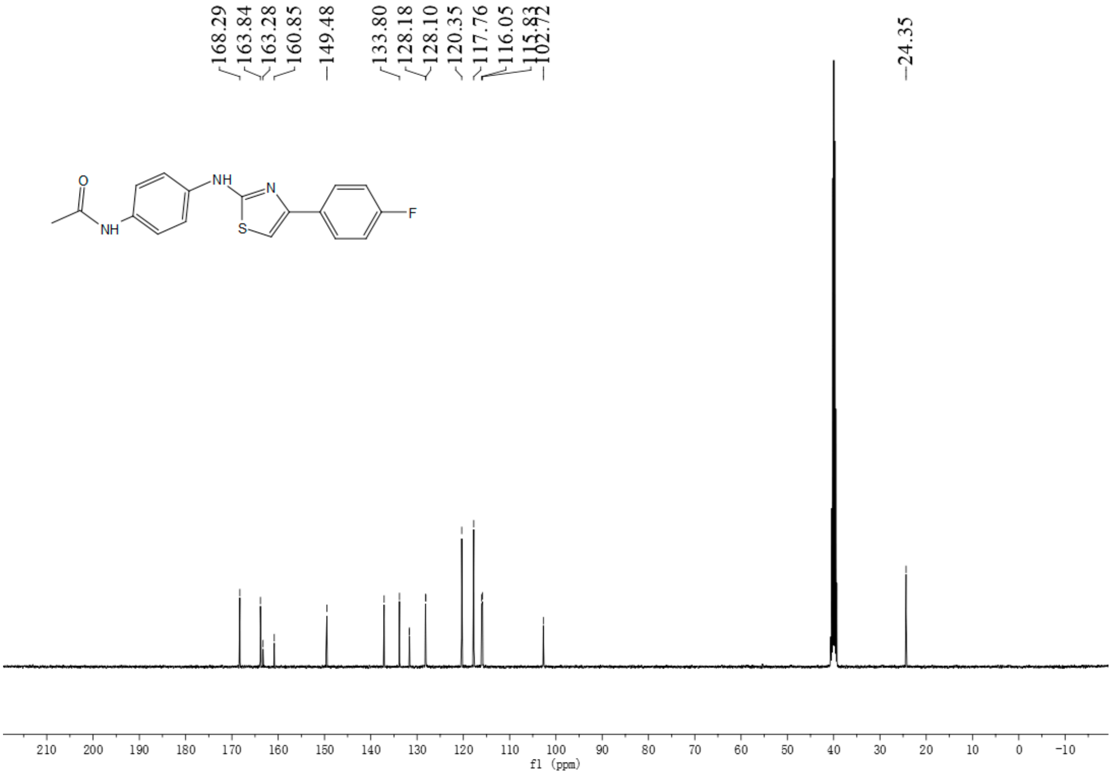

^{13}C NMR spectrum of compound **A**1

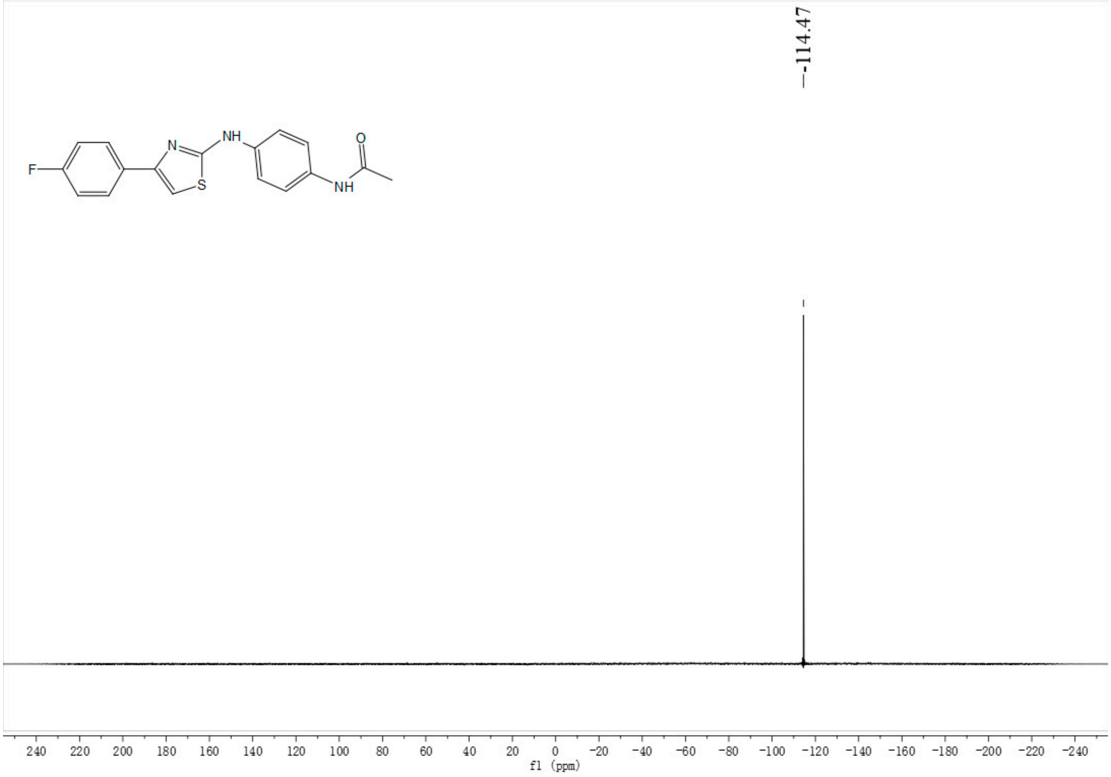

^{19}F NMR spectrum of compound **A**1

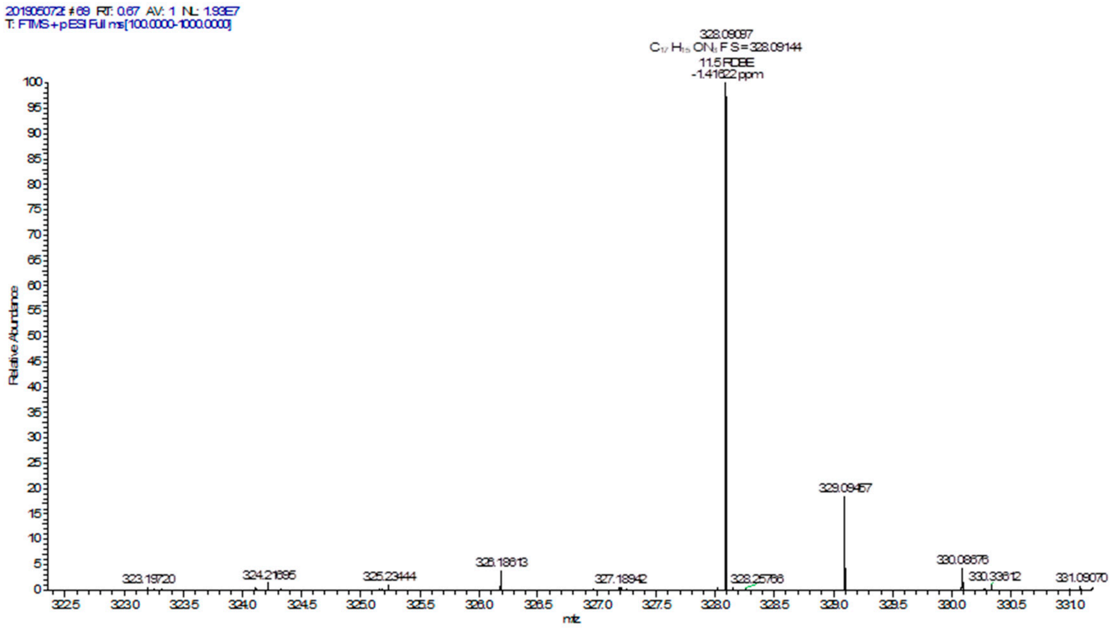

HRMS(ESI) of compound **A**1

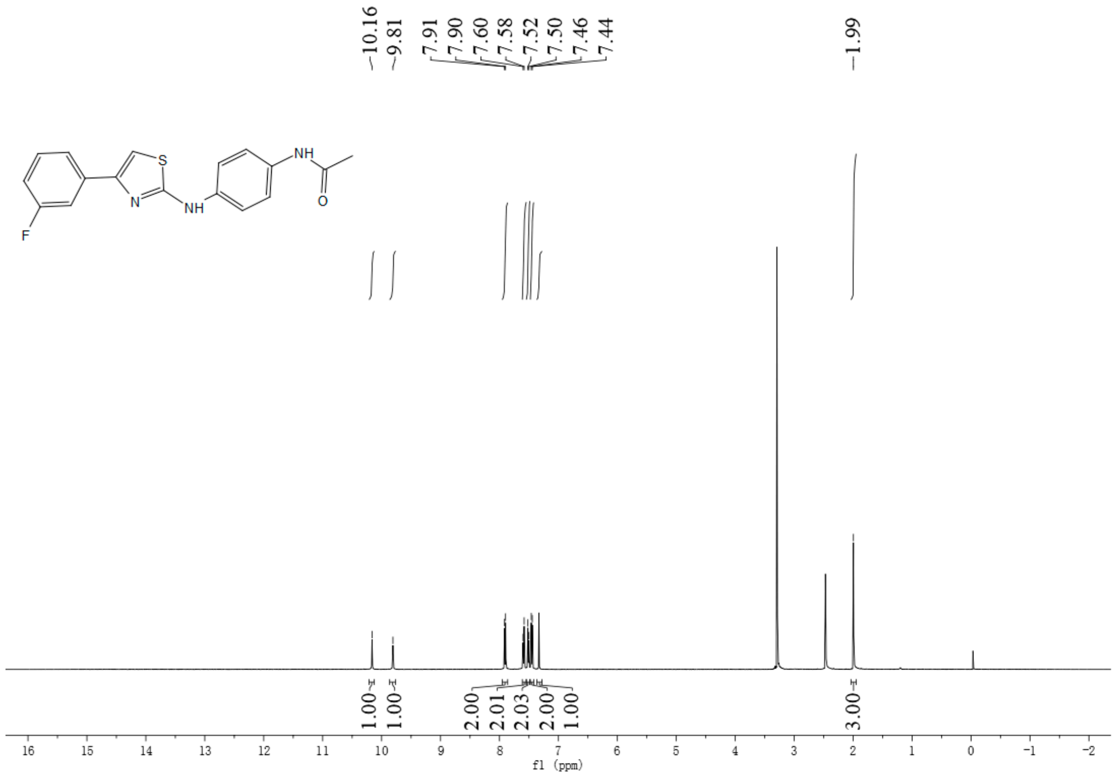

^{1}H NMR spectrum of compound **A**$_2$

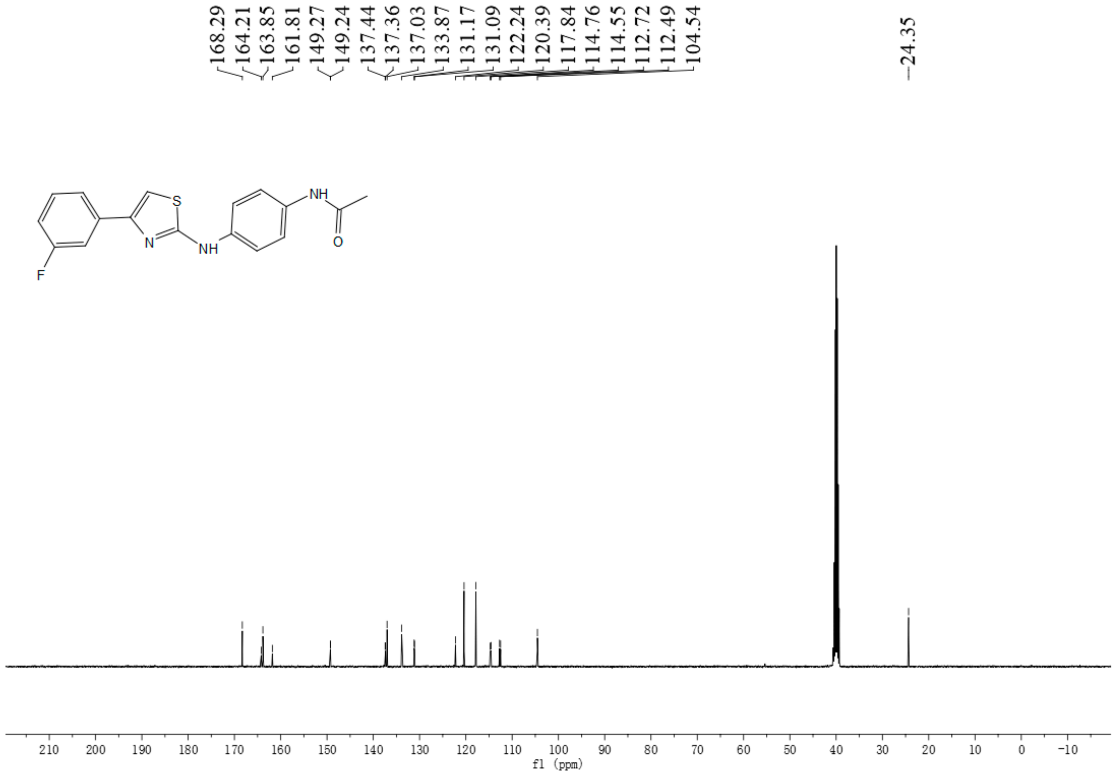

^{13}C NMR spectrum of compound **A**$_2$

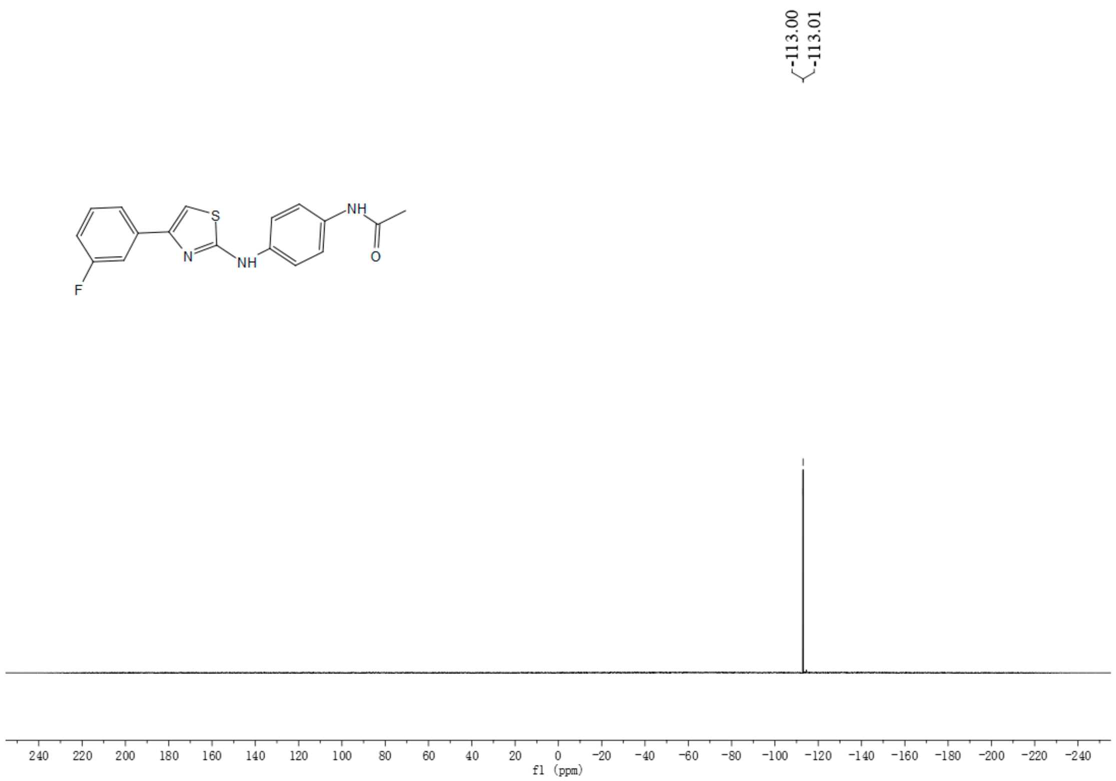

^{19}F NMR spectrum of compound **A**2

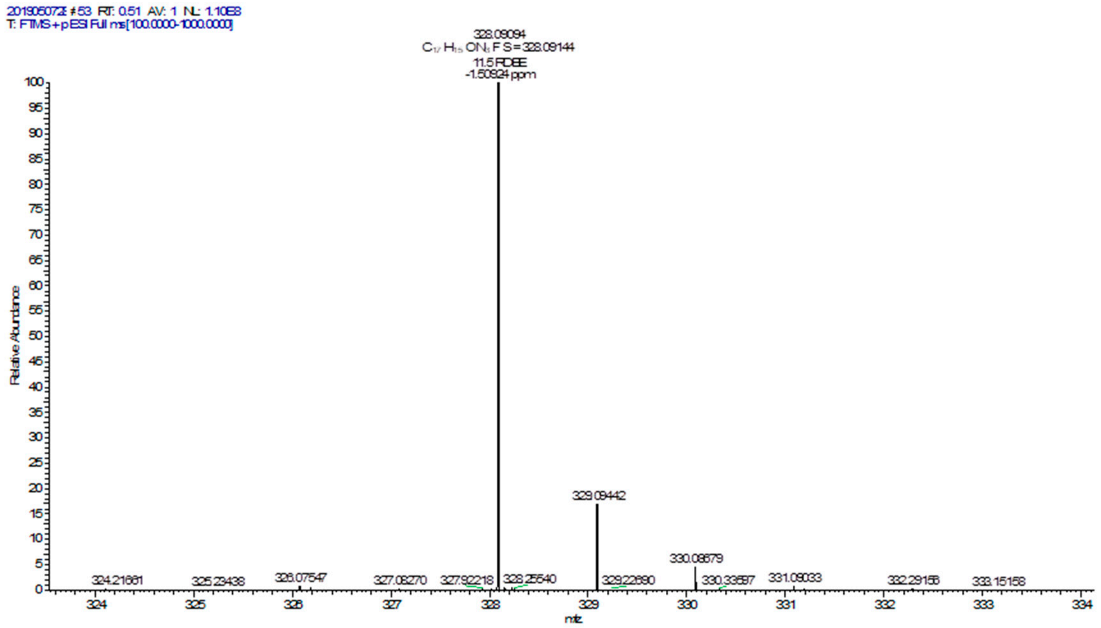

HRMS(ESI) of compound **A**2

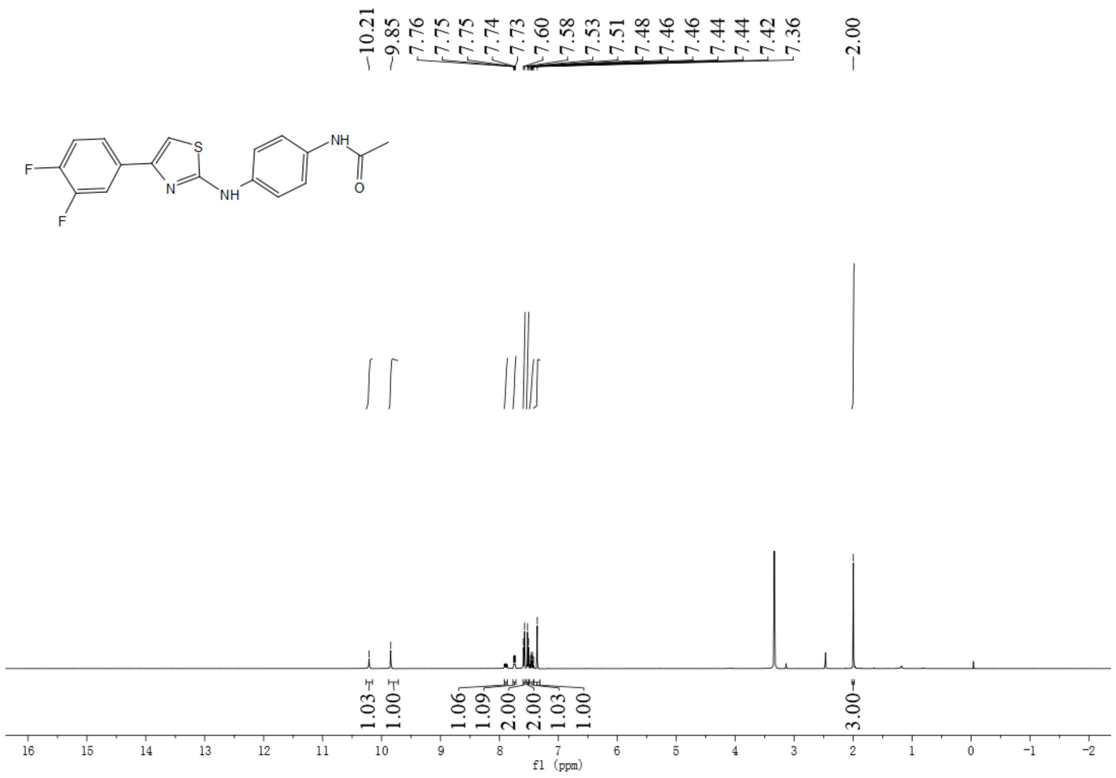

^{1}H NMR spectrum of compound **A**3

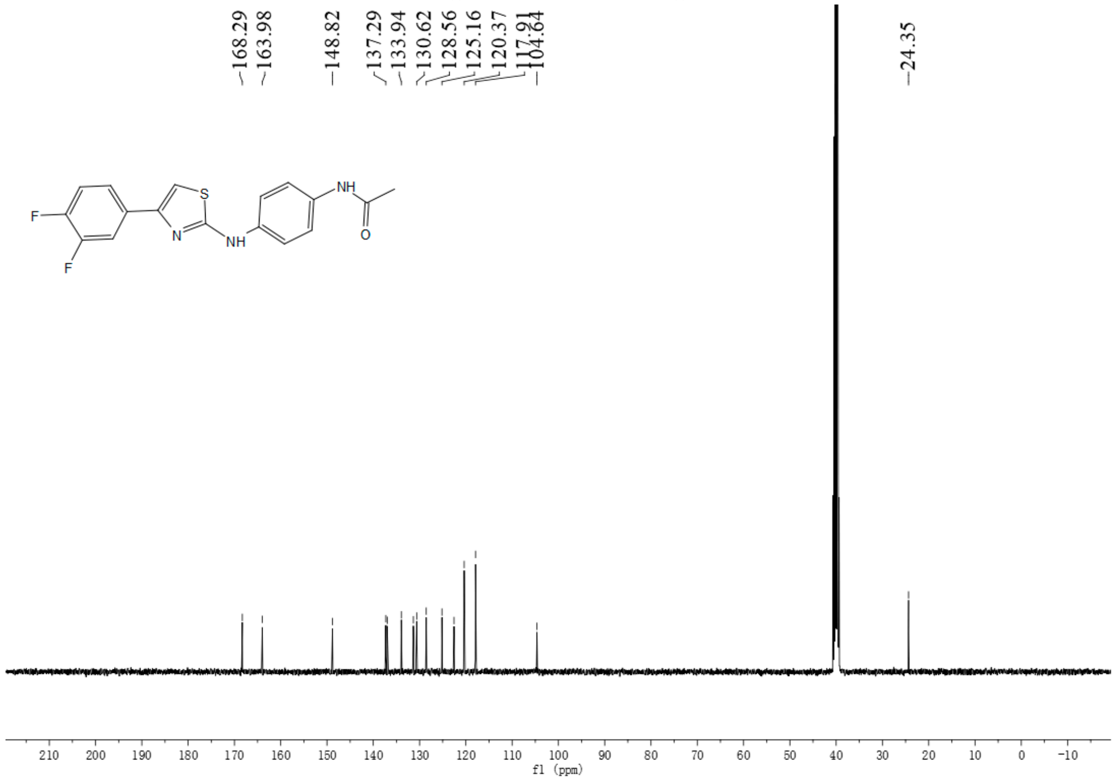

^{13}C NMR spectrum of compound **A**3

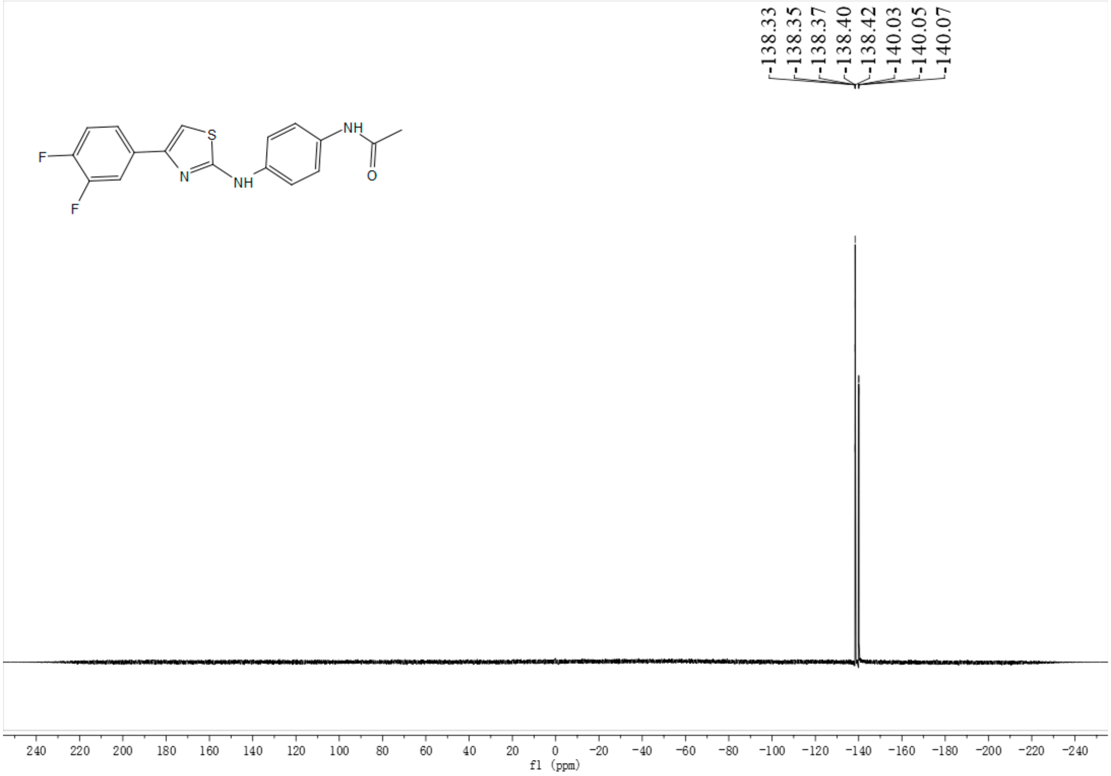

^{19}F NMR spectrum of compound **A**3

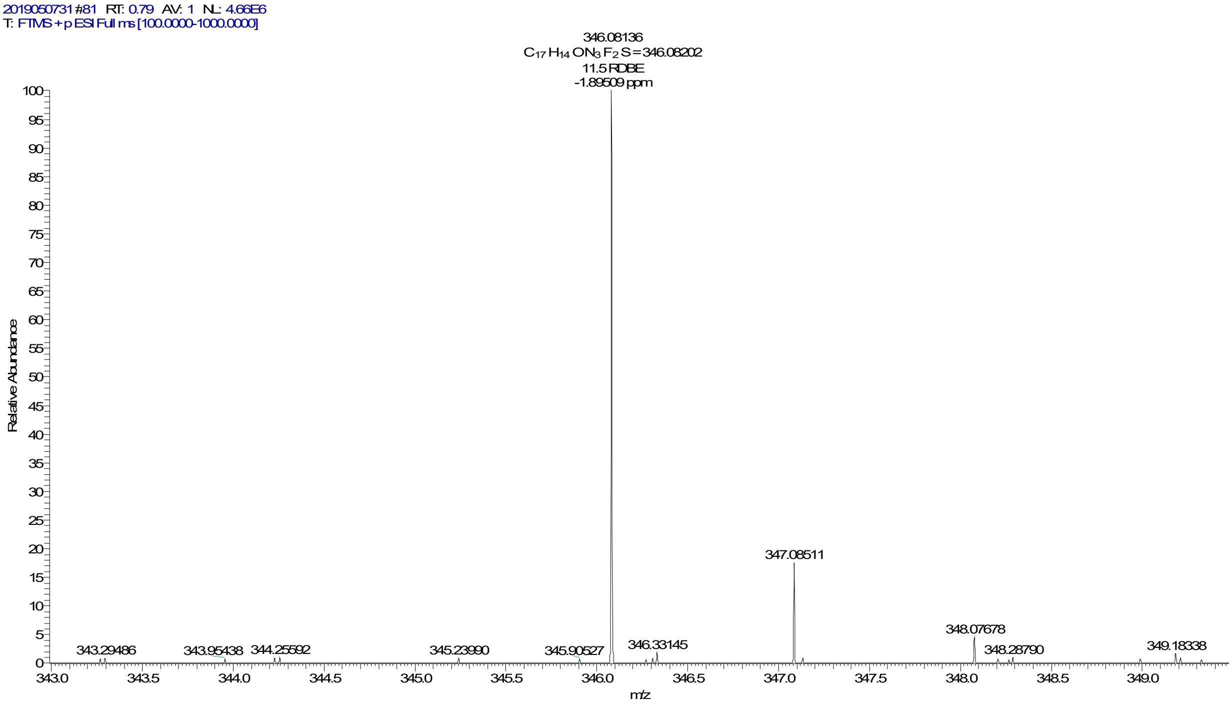

HRMS(ESI) of compound **A**3

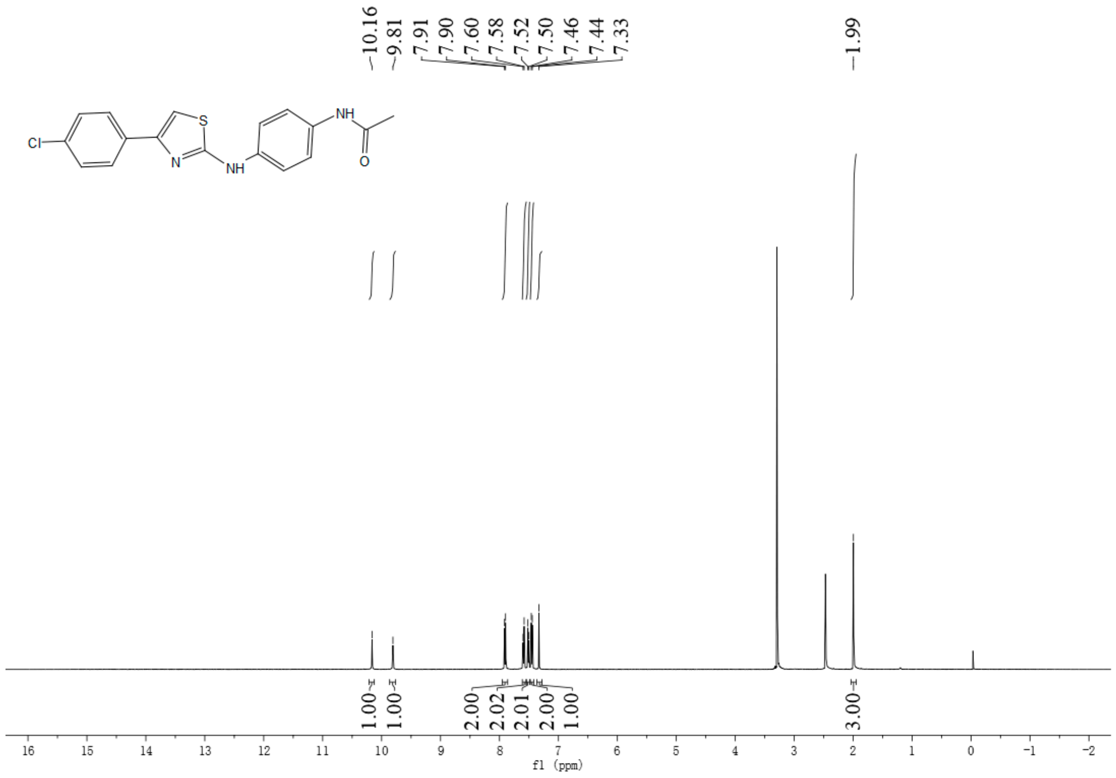

^{1}H NMR spectrum of compound **A**$_4$

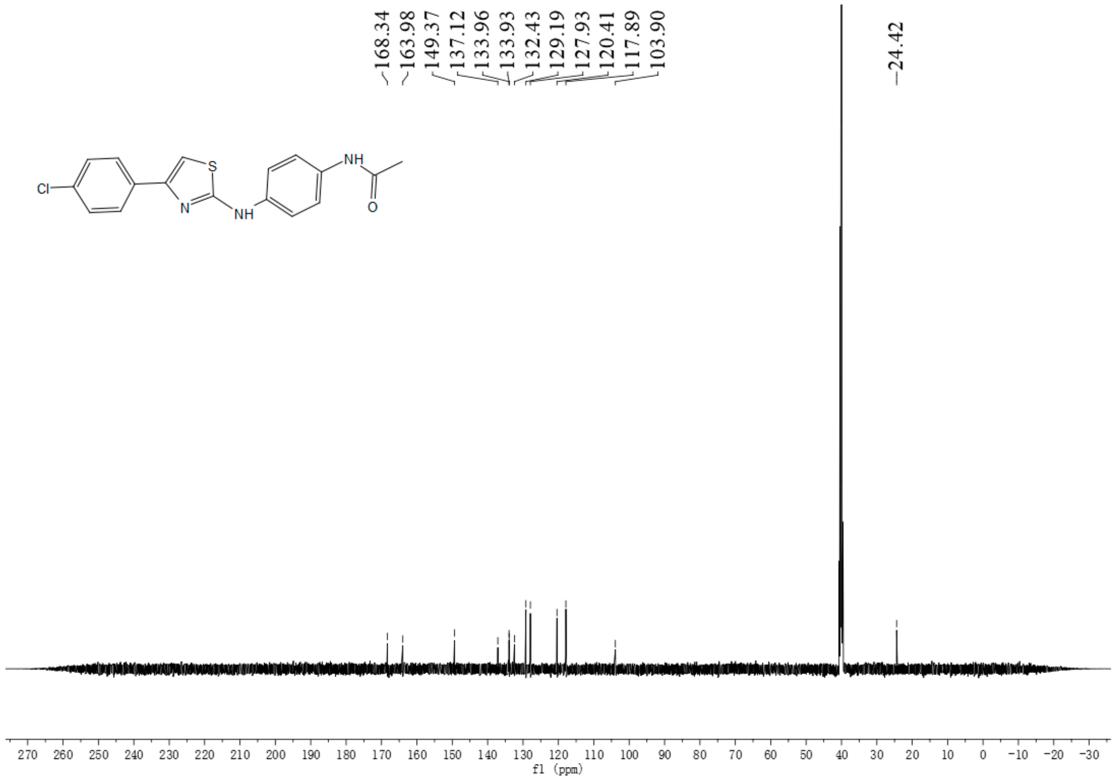

^{13}C NMR spectrum of compound **A**$_4$

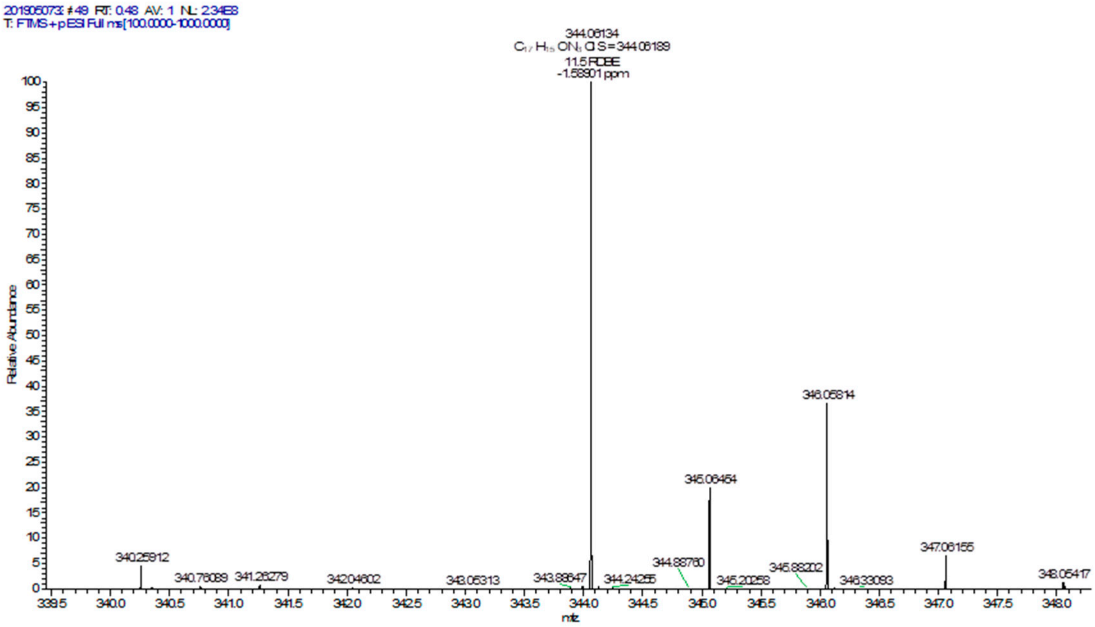

HRMS(ESI) of compound **A**4

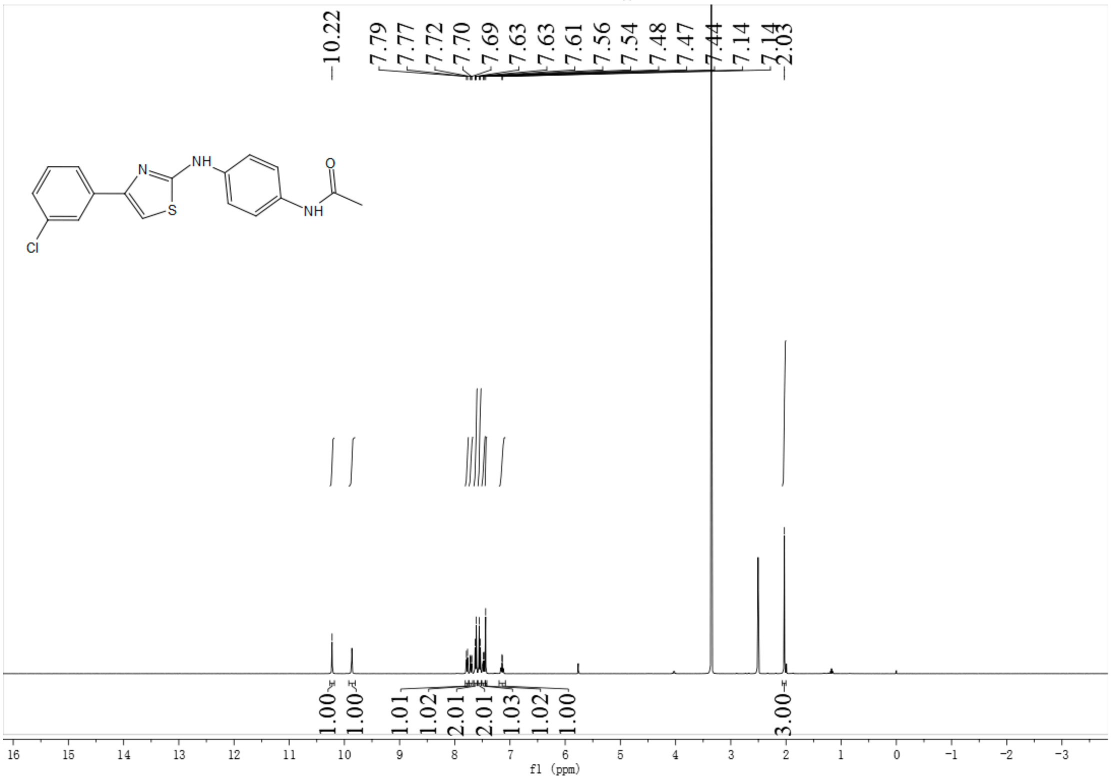

¹H NMR spectrum of compound **A**5

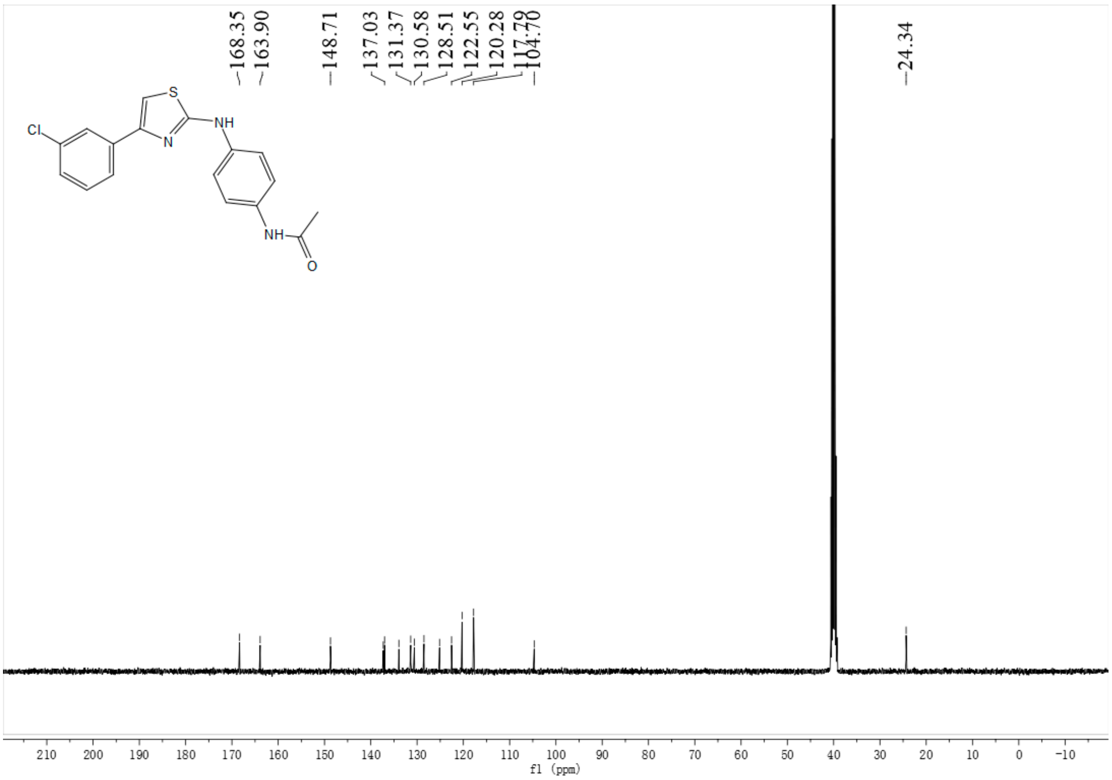

^{13}C NMR spectrum of compound **A**5

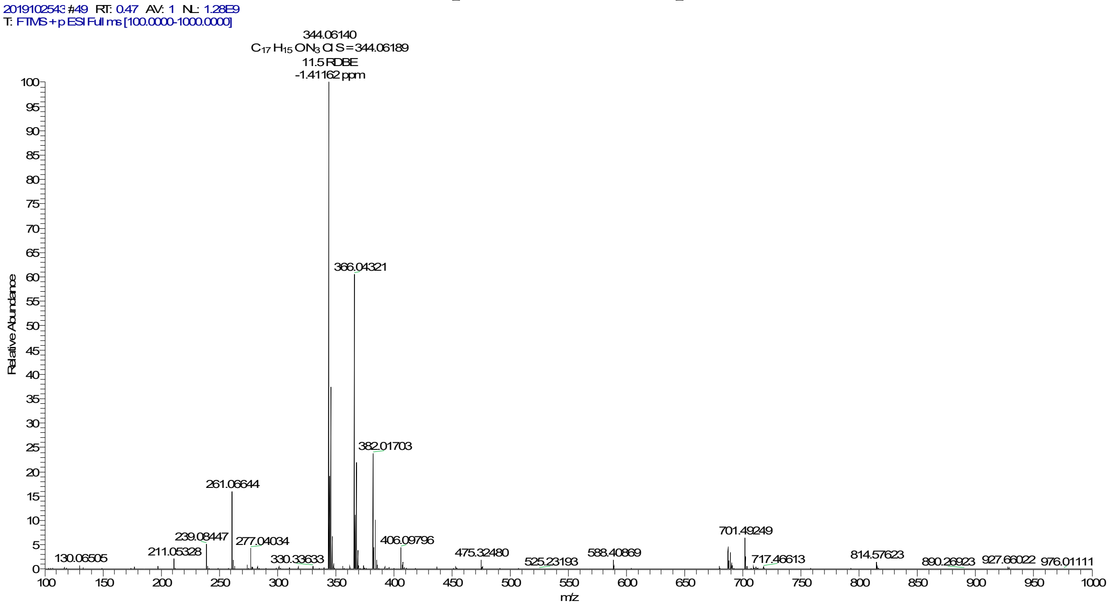

HRMS(ESI) of compound **A**5

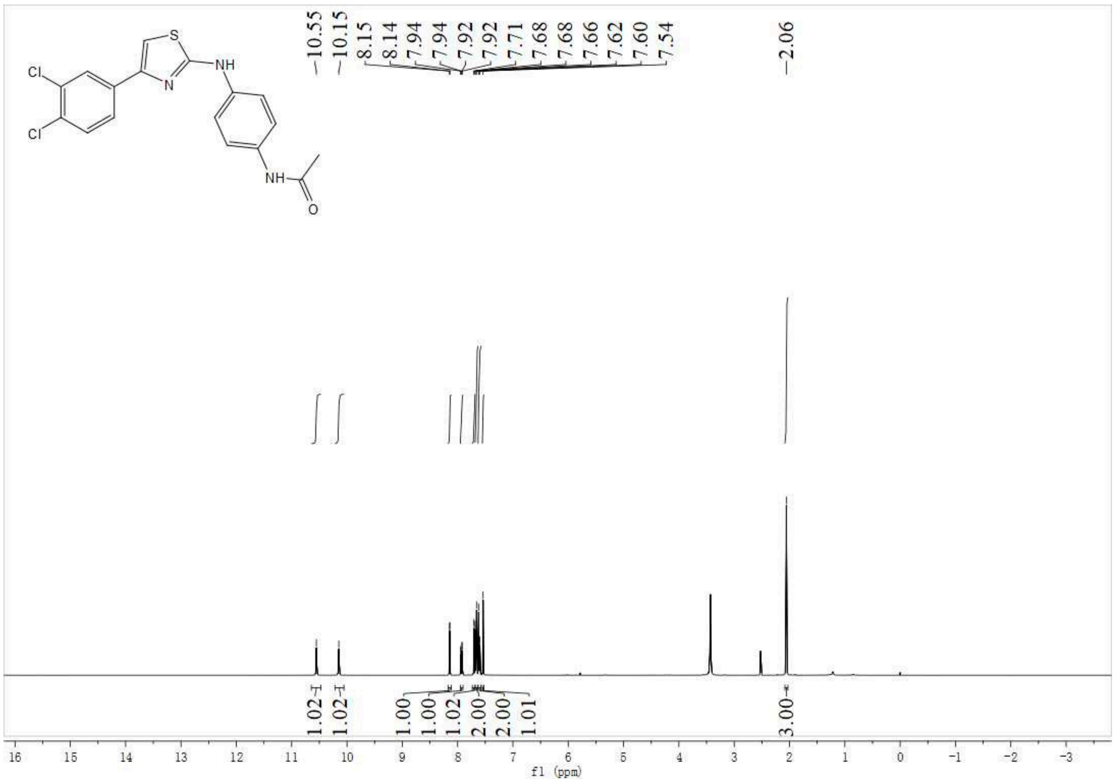

^{1}H NMR spectrum of compound **A**$_6$

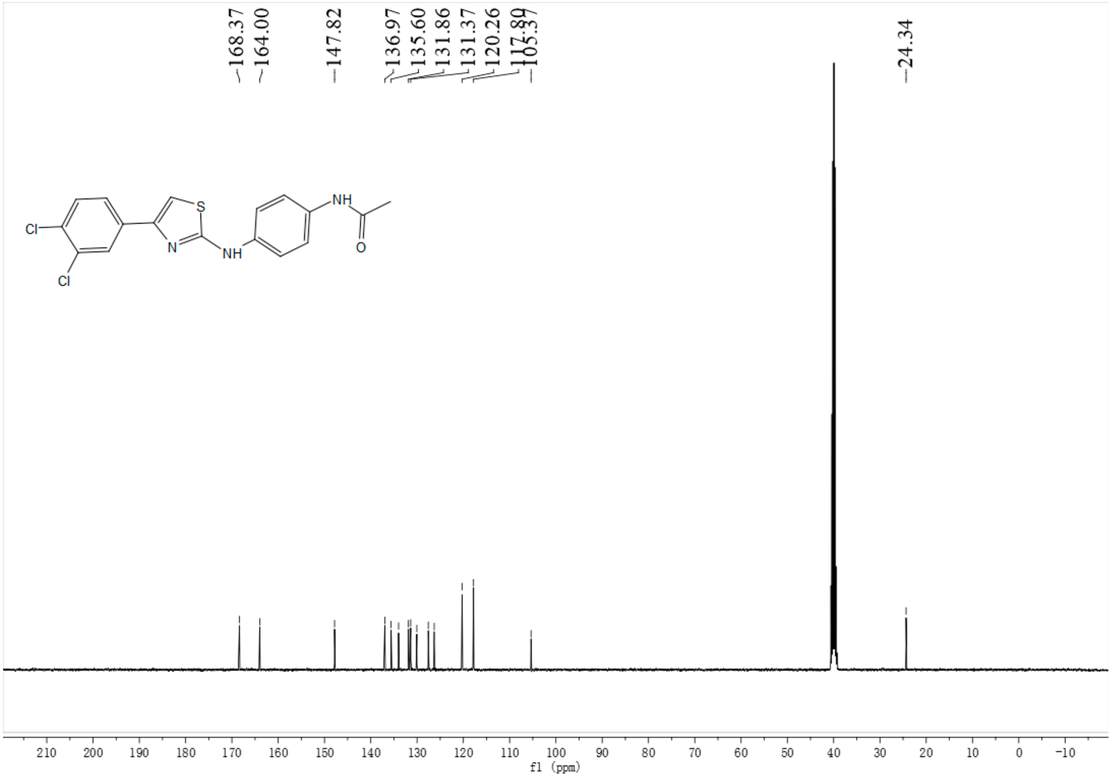

^{13}C NMR spectrum of compound **A**$_6$

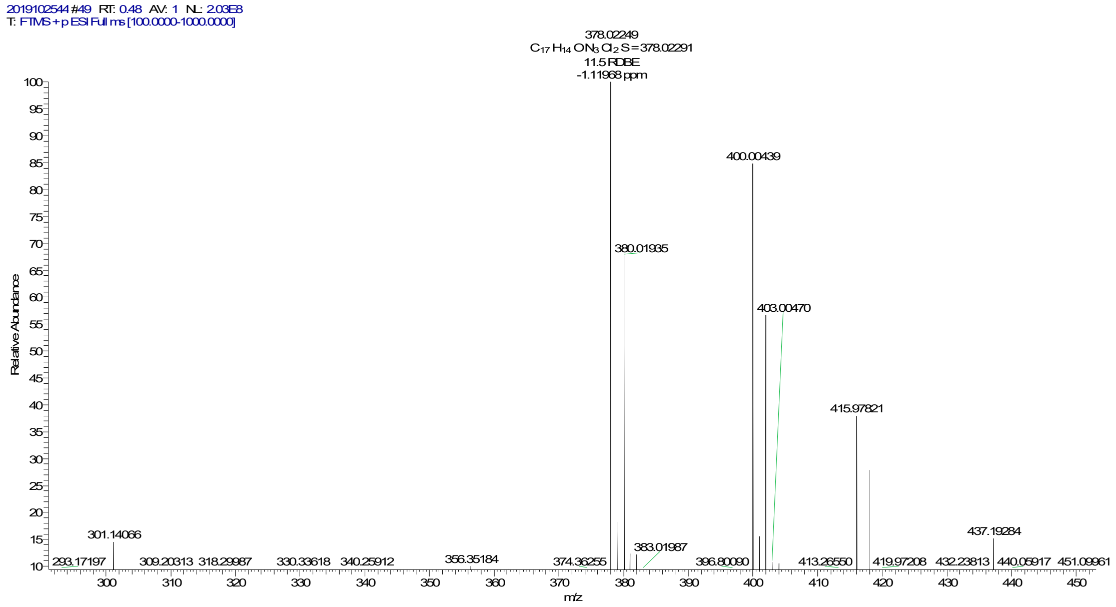

HRMS(ESI) of compound **A**$_6$

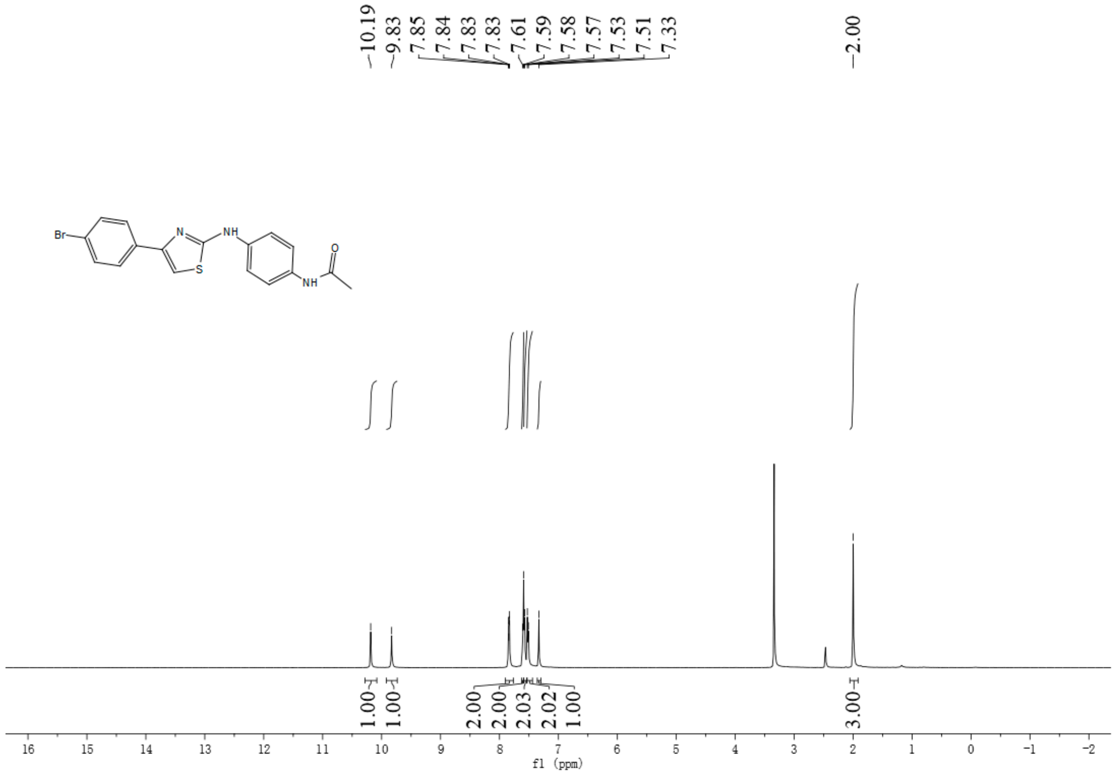

^{1}H NMR spectrum of compound **A**$_7$

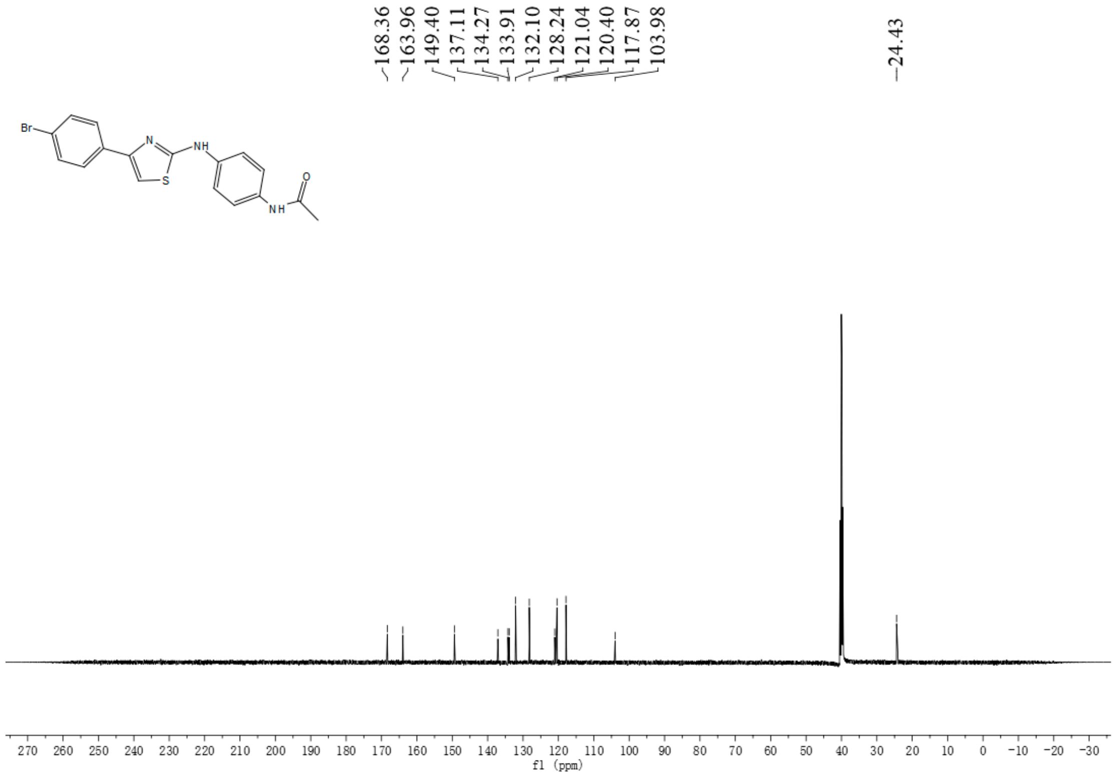

^{13}C NMR spectrum of compound **A**7

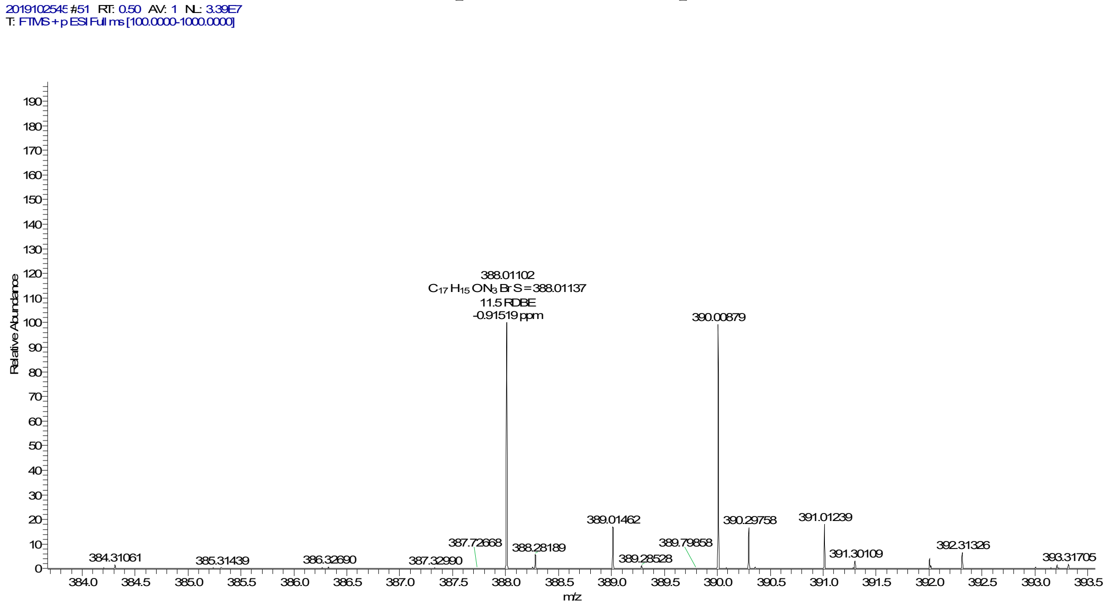

HRMS(ESI) of compound **A**7

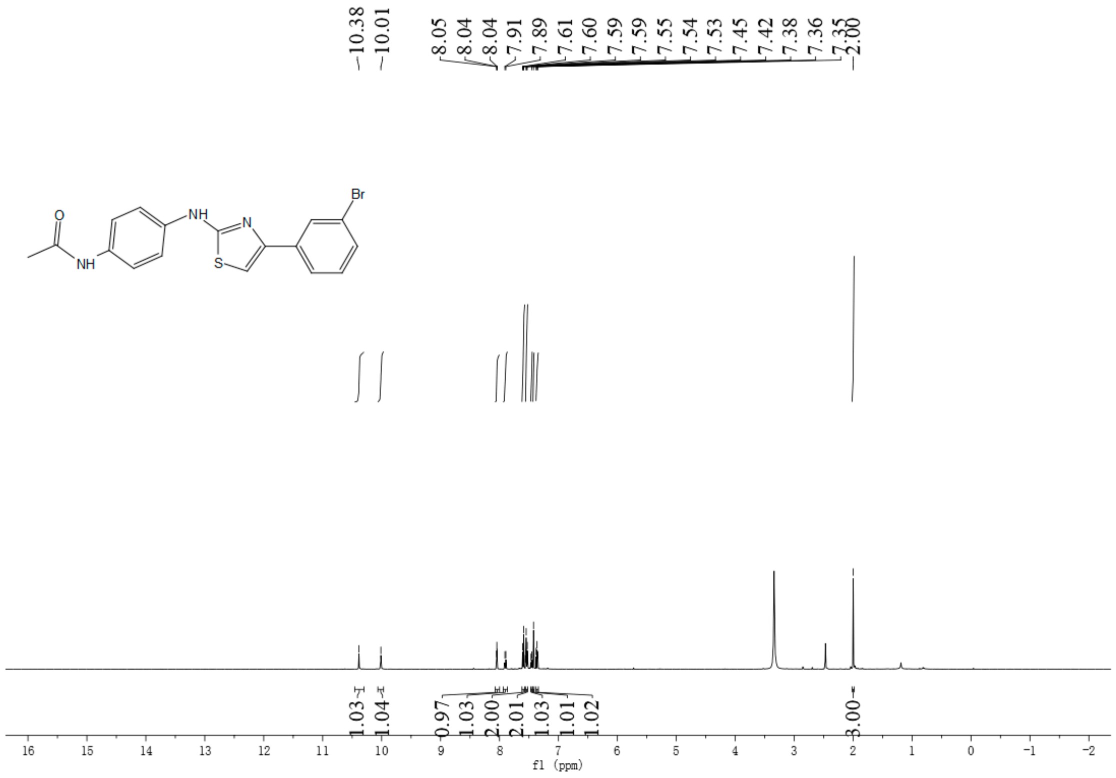

^{1}H NMR spectrum of compound **A**$_8$

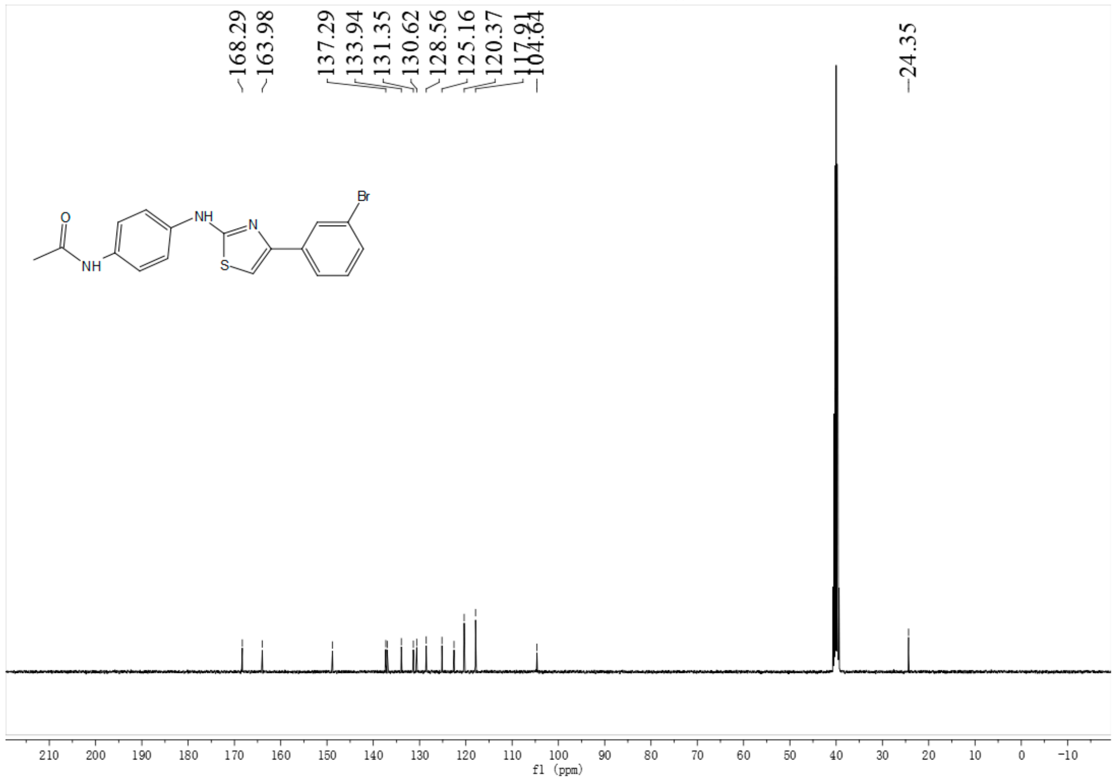

^{13}C NMR spectrum of compound **A**$_8$

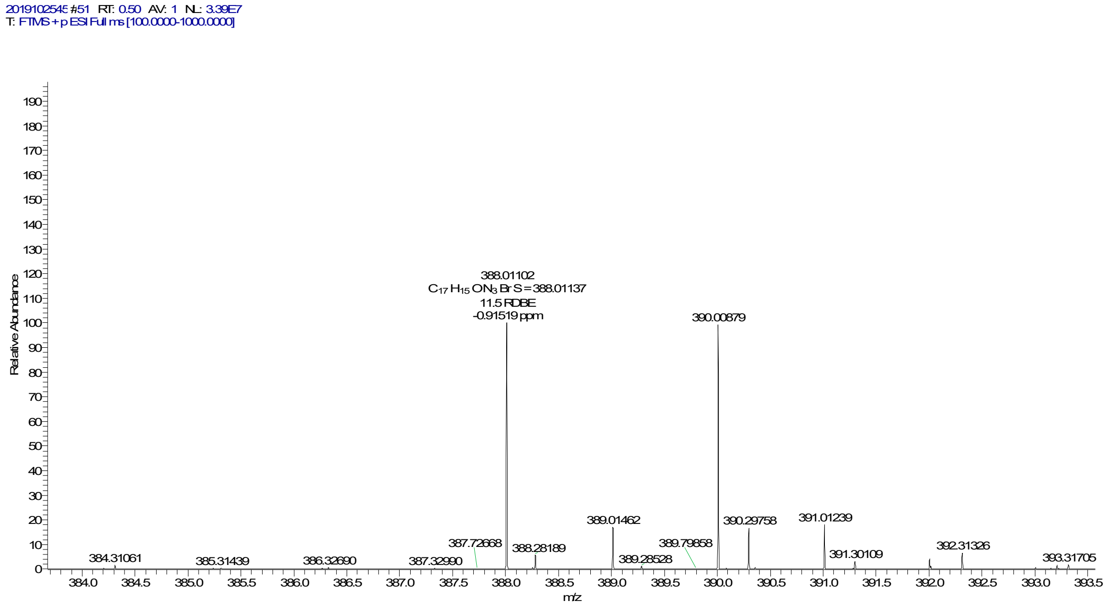

HRMS(ESI) of compound **A**8

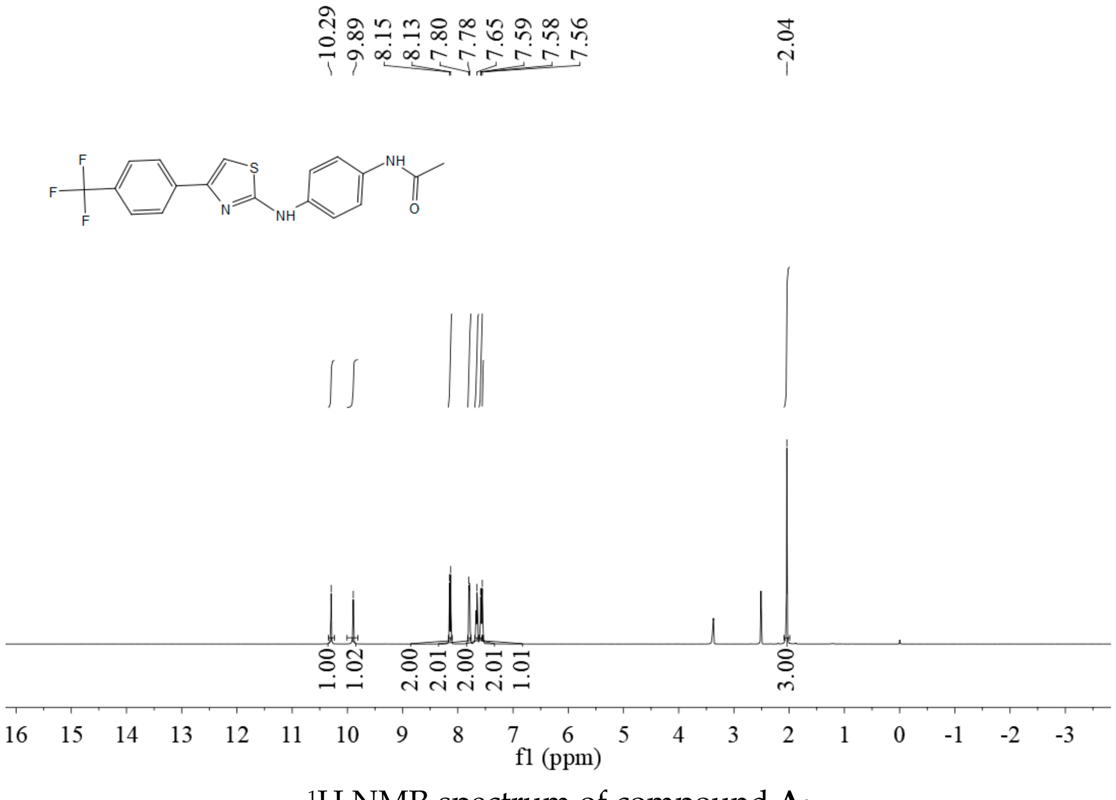

^{1}H NMR spectrum of compound **A**9

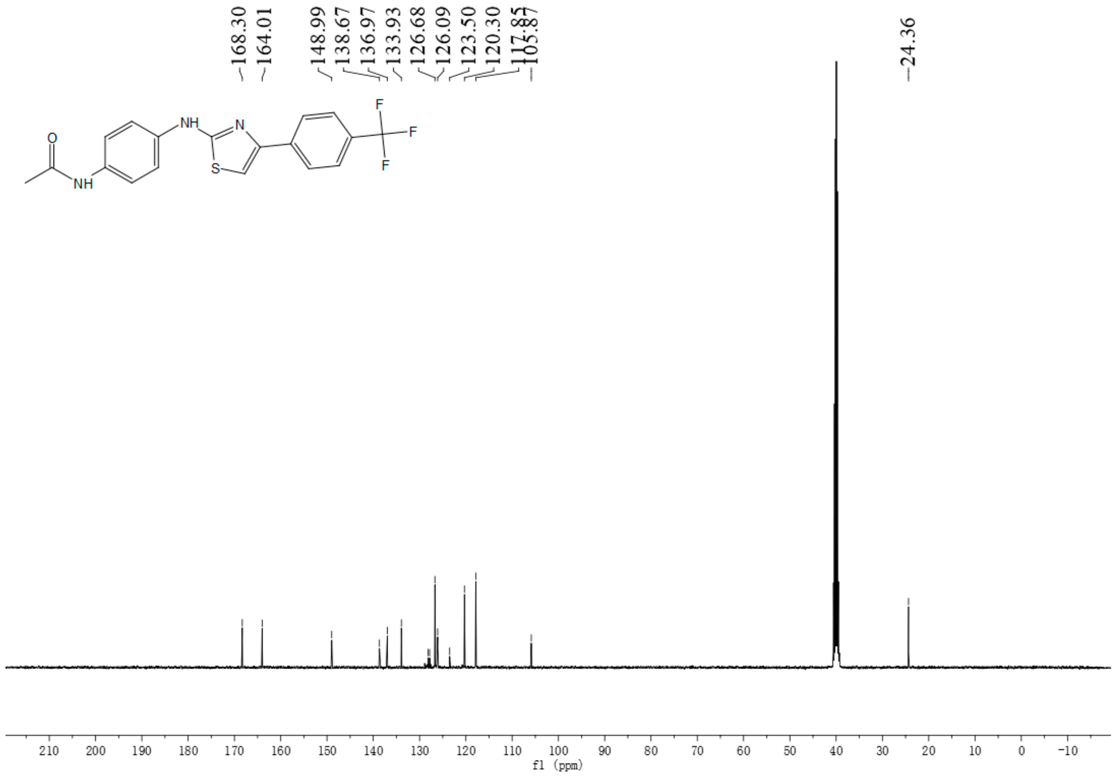

^{13}C NMR spectrum of compound **A**9

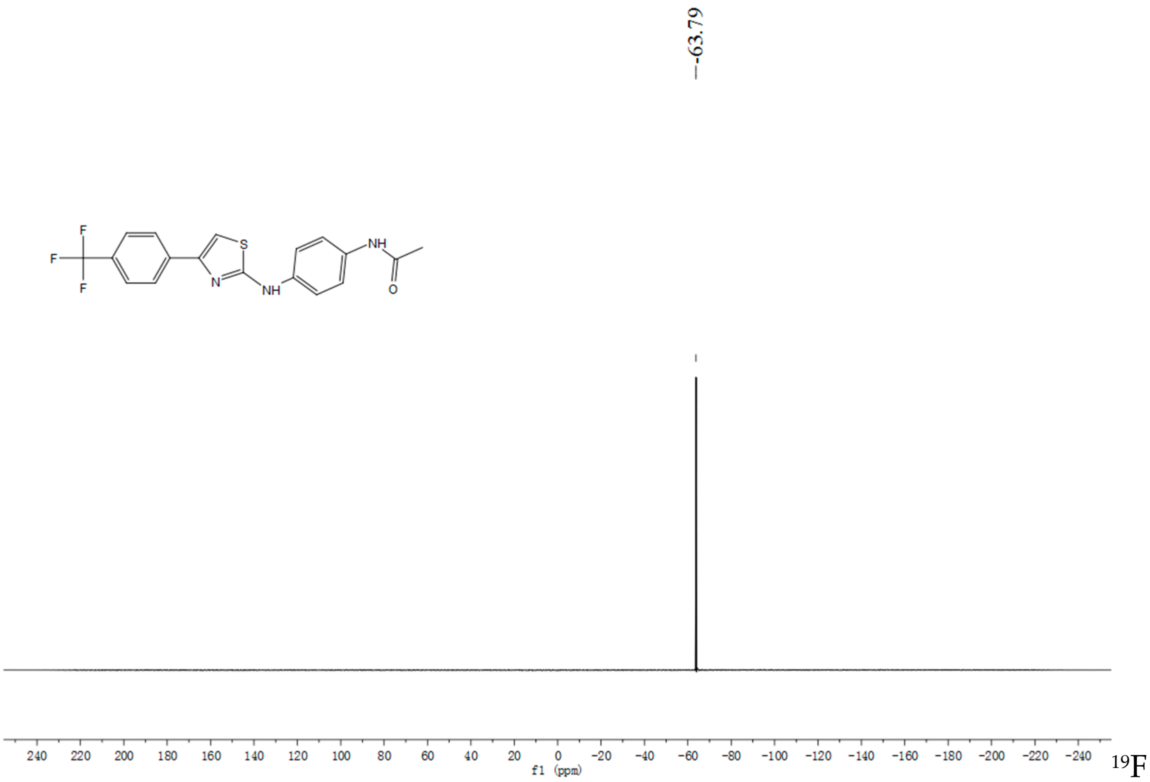

^{19}F NMR spectrum of compound **A**9

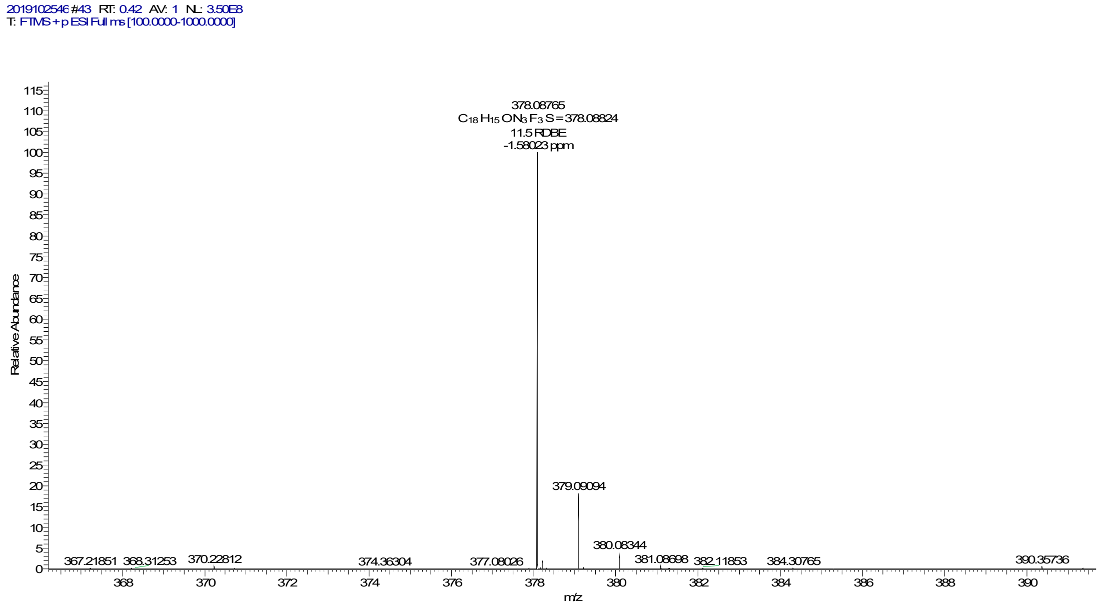

HRMS of compound **A**9

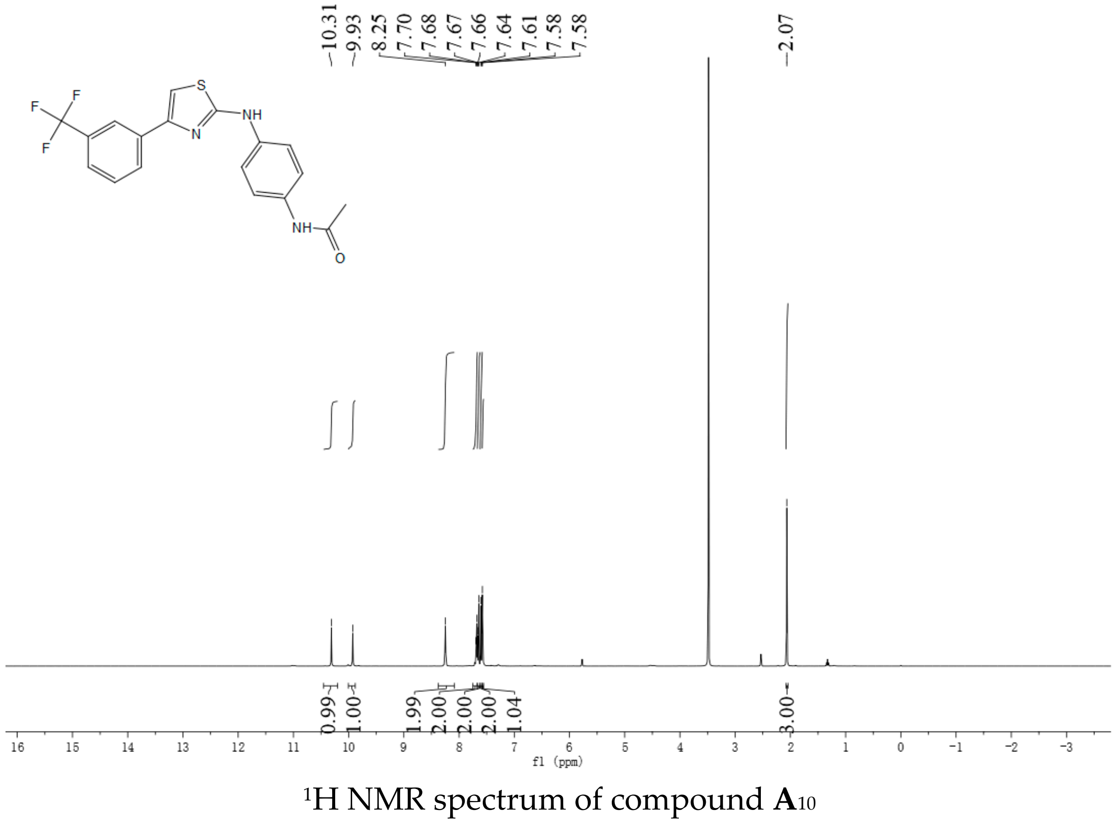

^{1}H NMR spectrum of compound **A**10

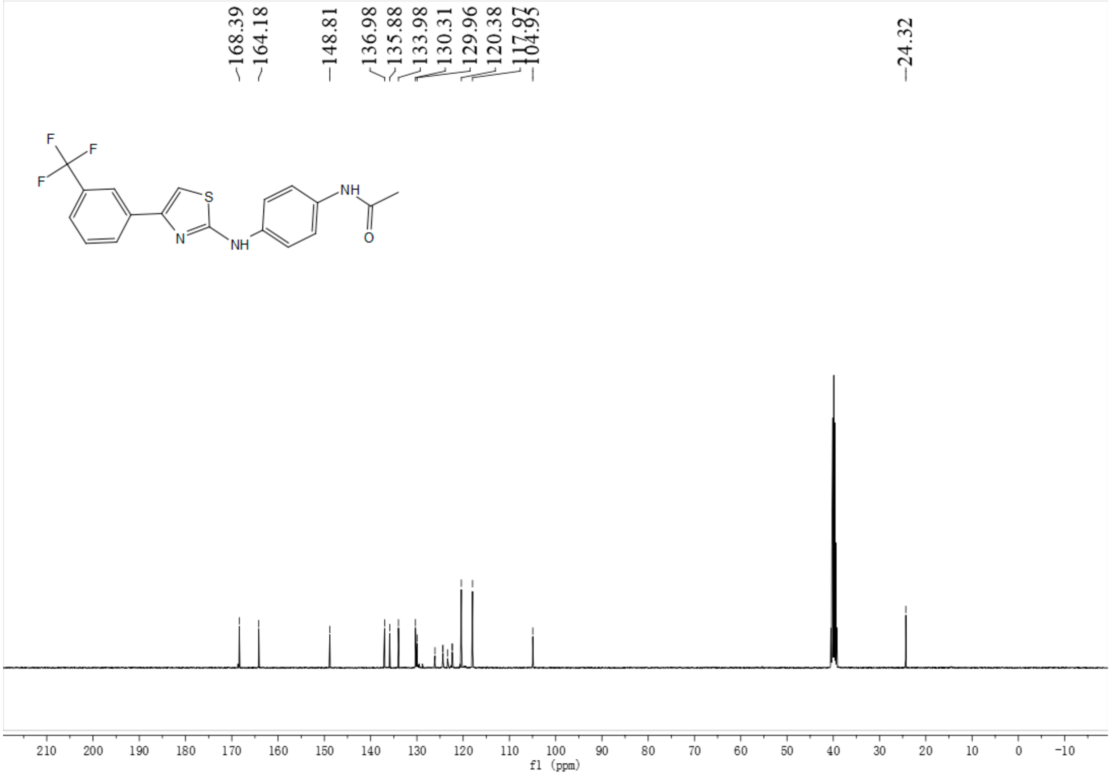

^{13}C NMR spectrum of compound **A**10

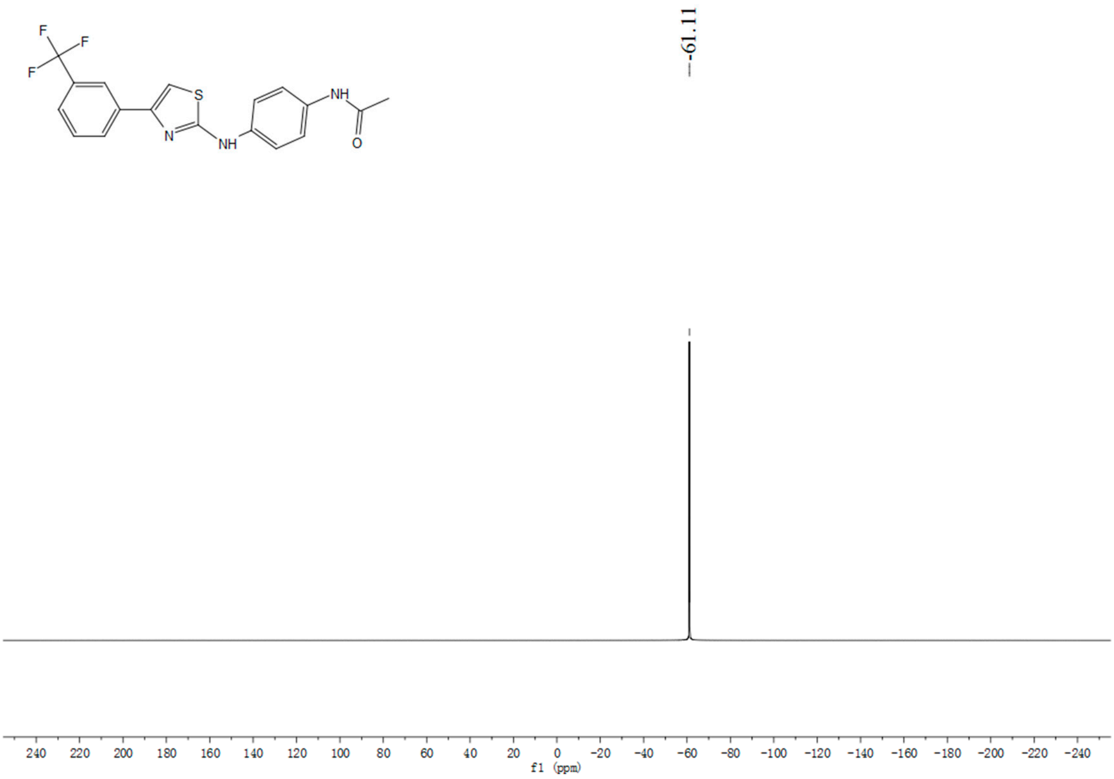

^{19}F NMR spectrum of compound **A**10

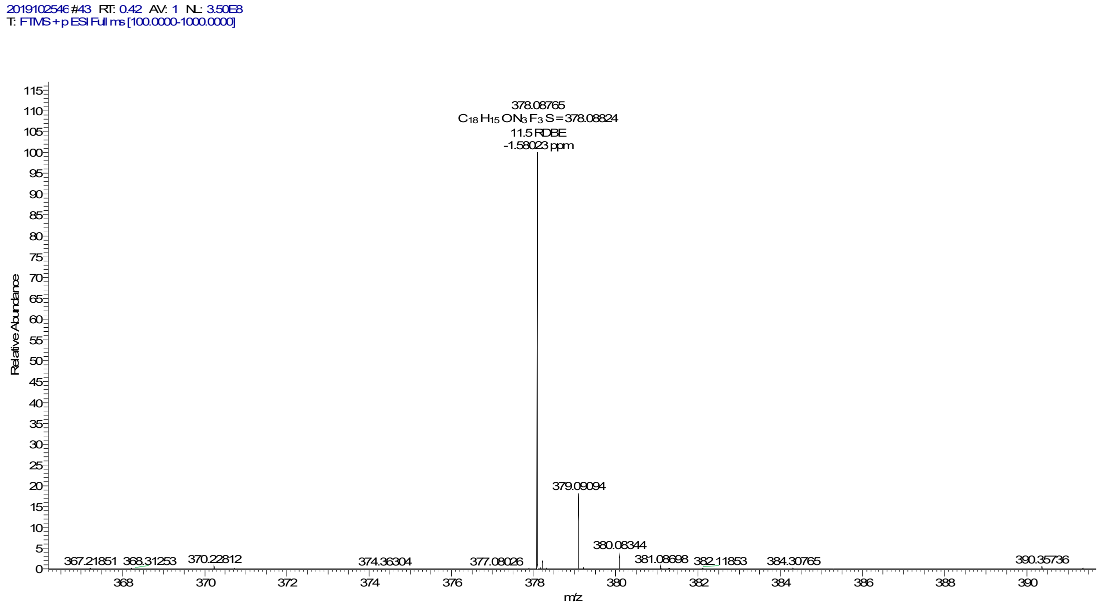

HRMS of compound **A**10

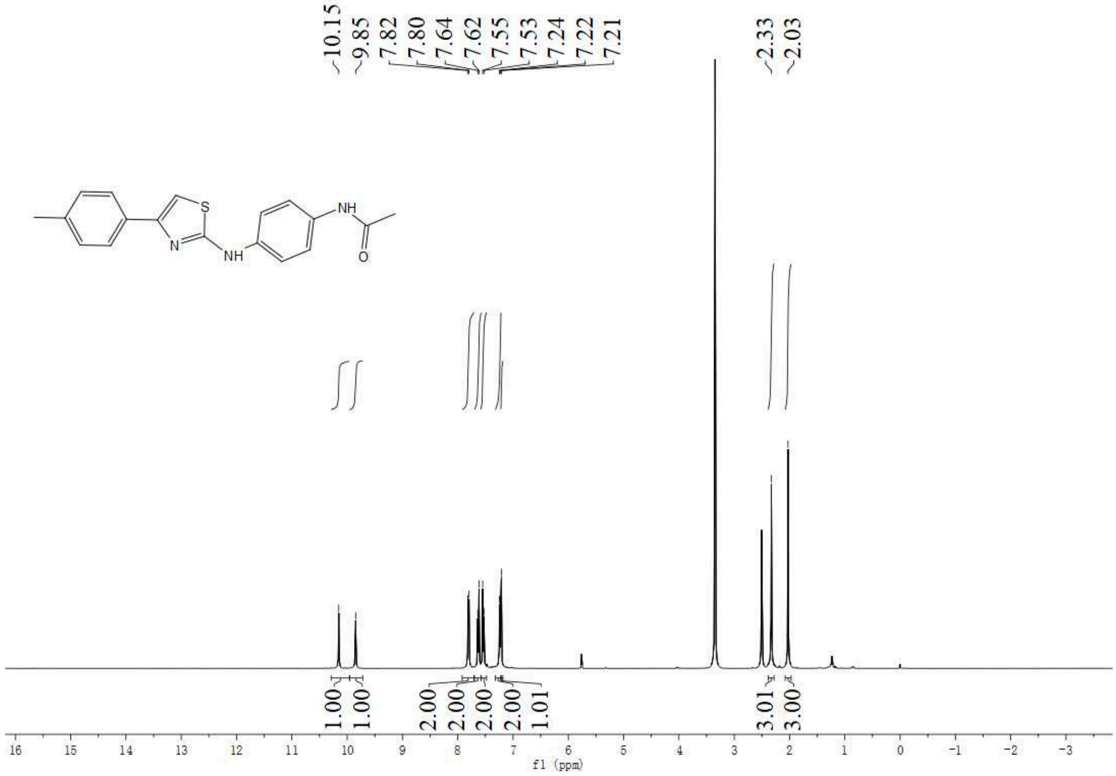

^{1}H NMR spectrum of compound **A**11

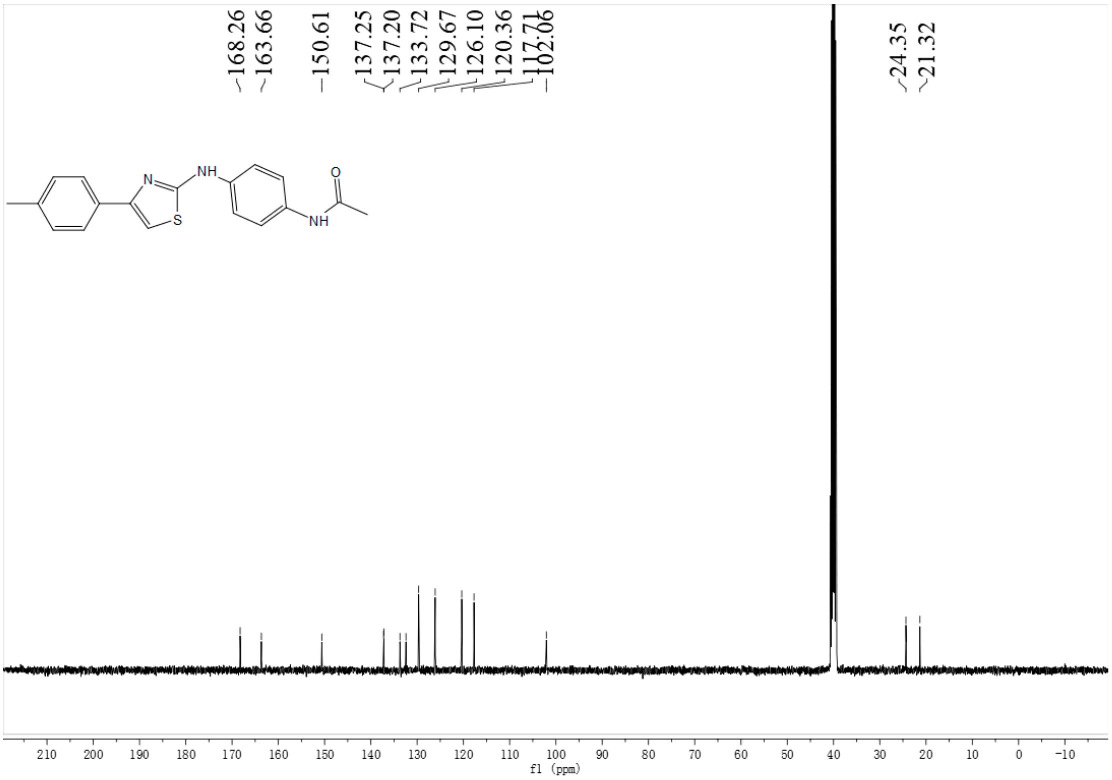

^{13}C NMR spectrum of compound **A**11

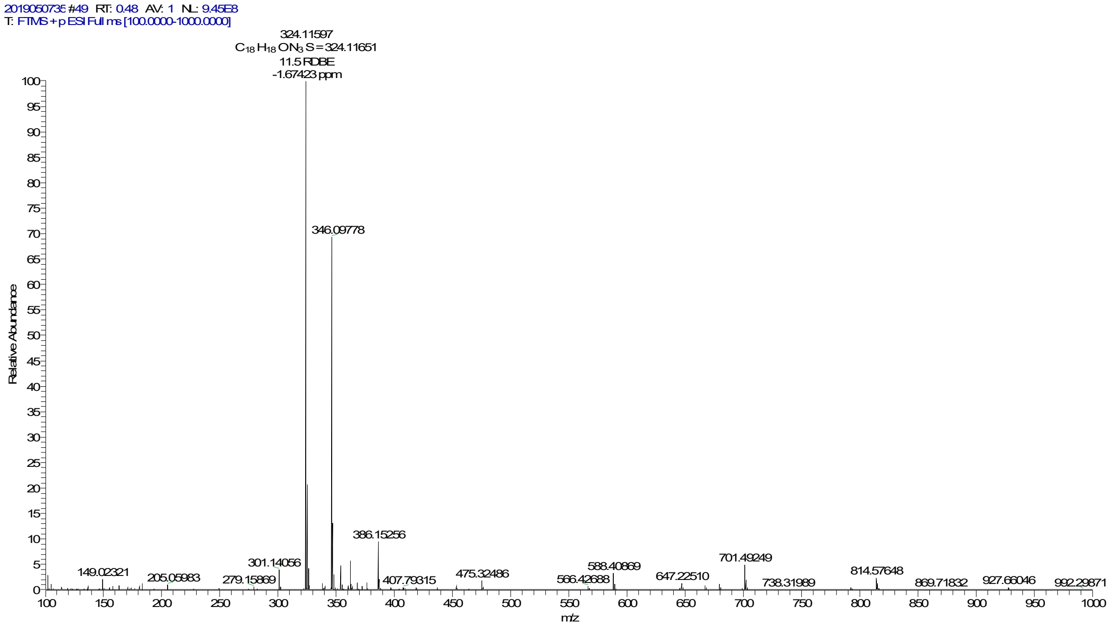

HRMS(ESI) of compound **A**11

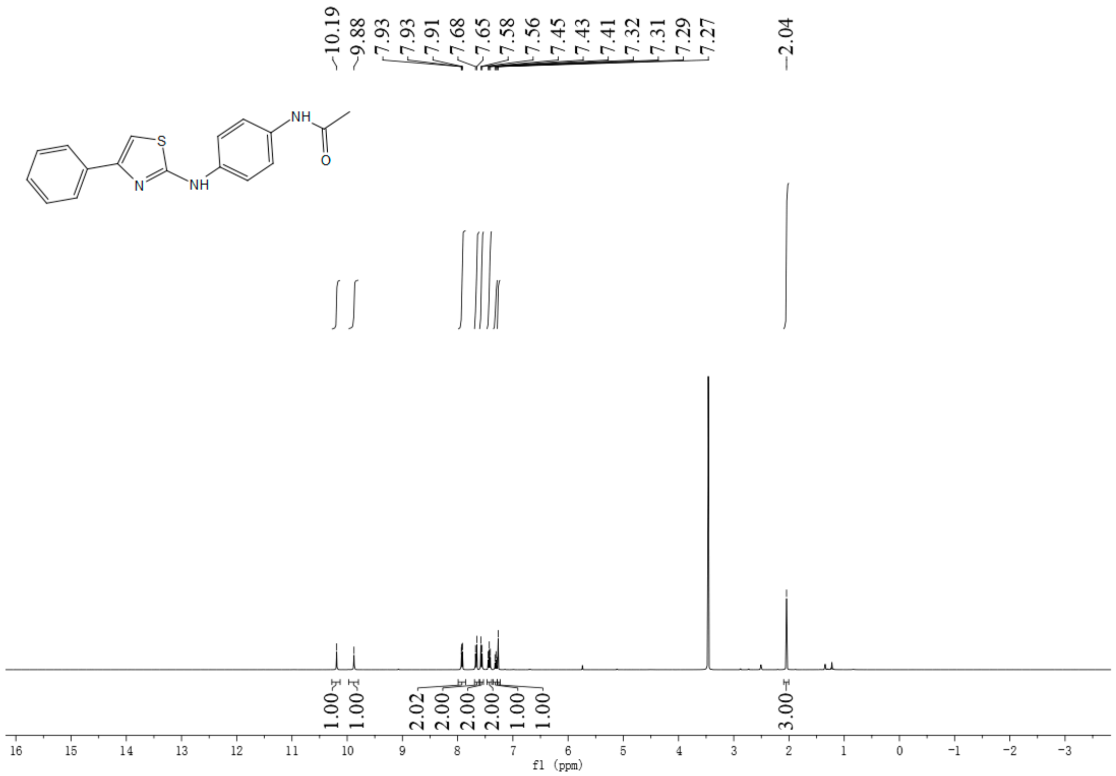

^{1}H NMR spectrum of compound **A**12

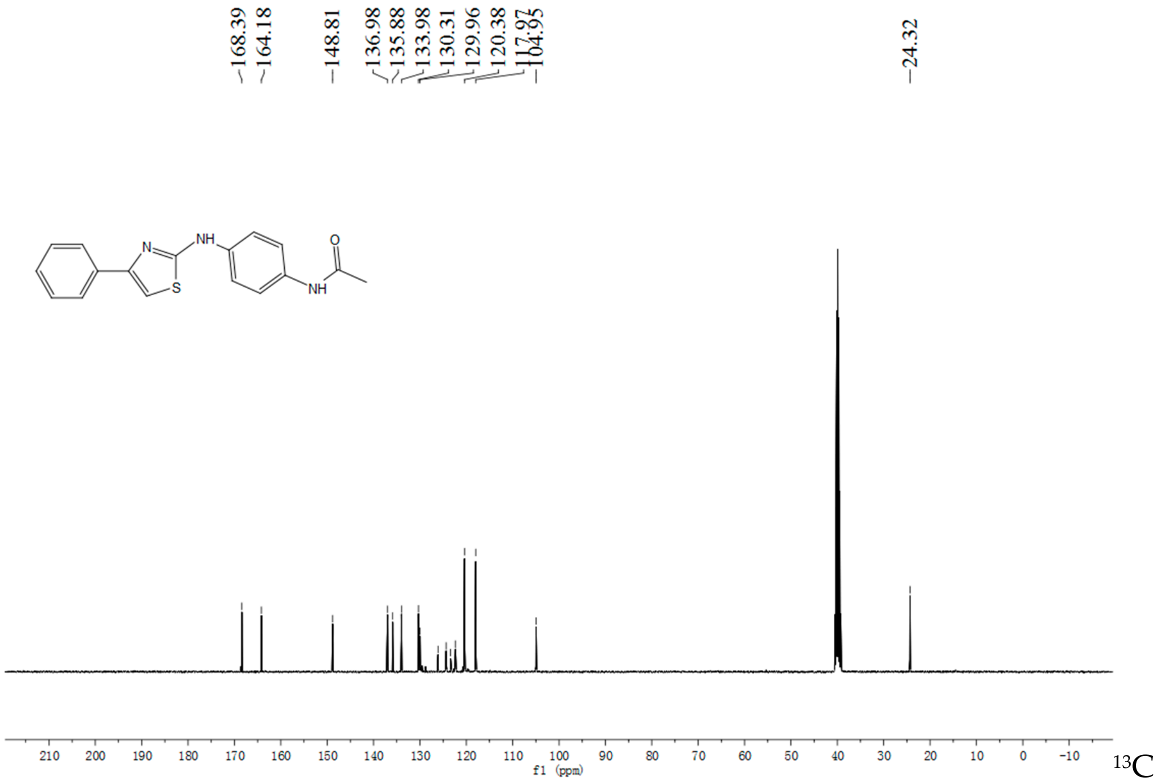

^{13}C NMR spectrum of compound **A**12

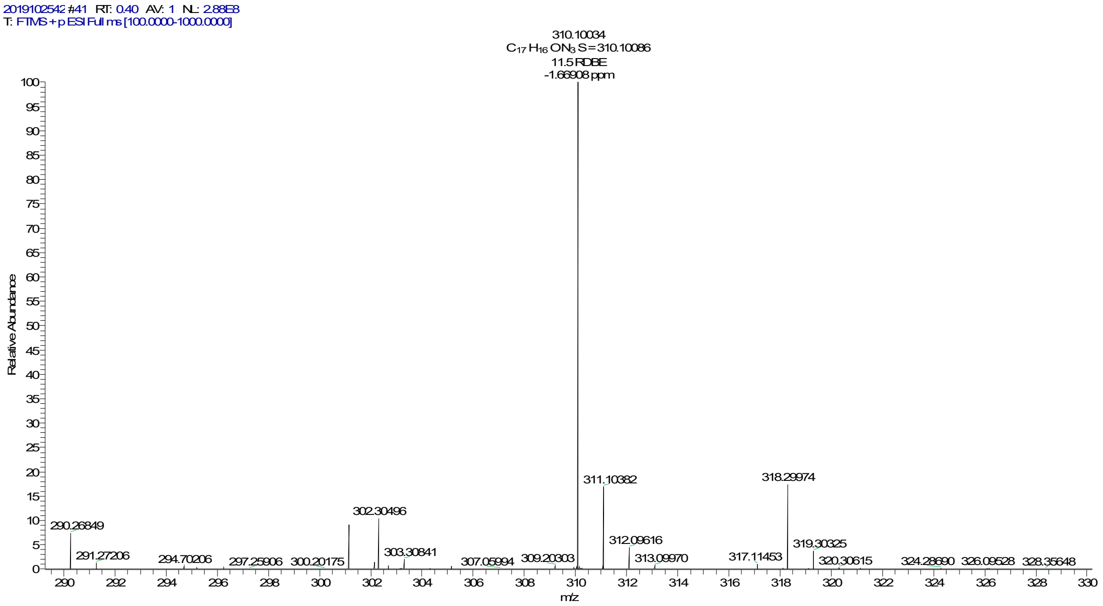

HRMS(ESI) of compound **A**12

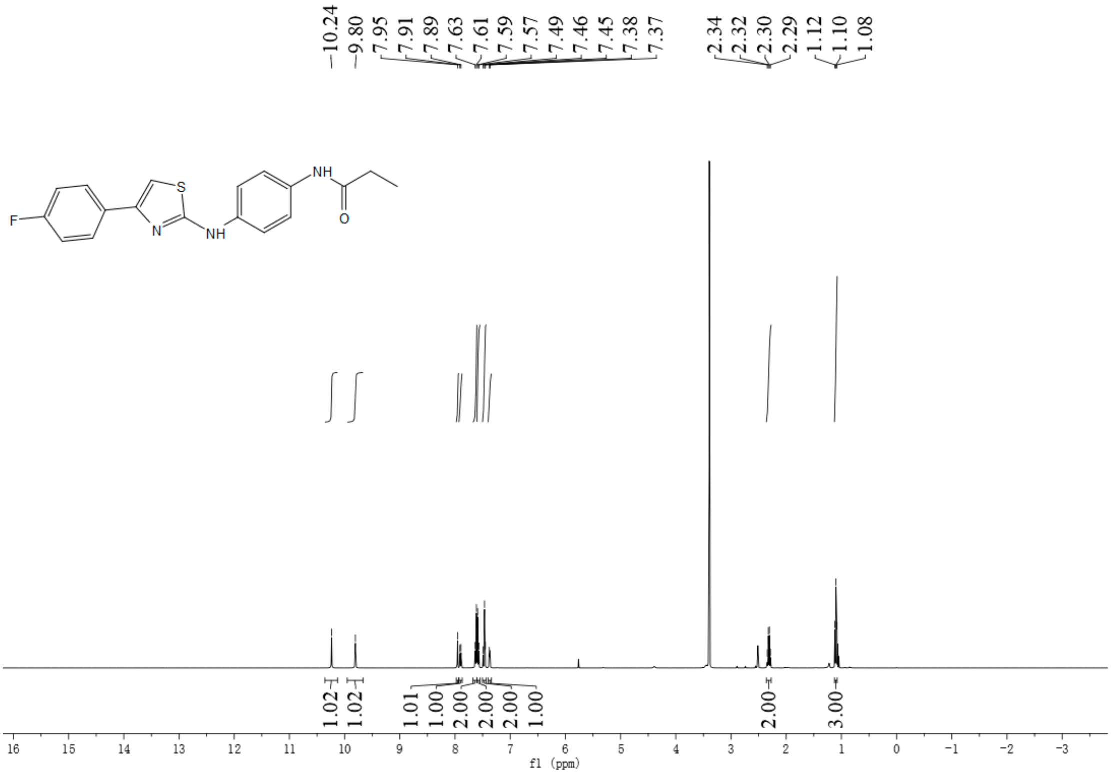

^{1}H NMR spectrum of compound **A**13

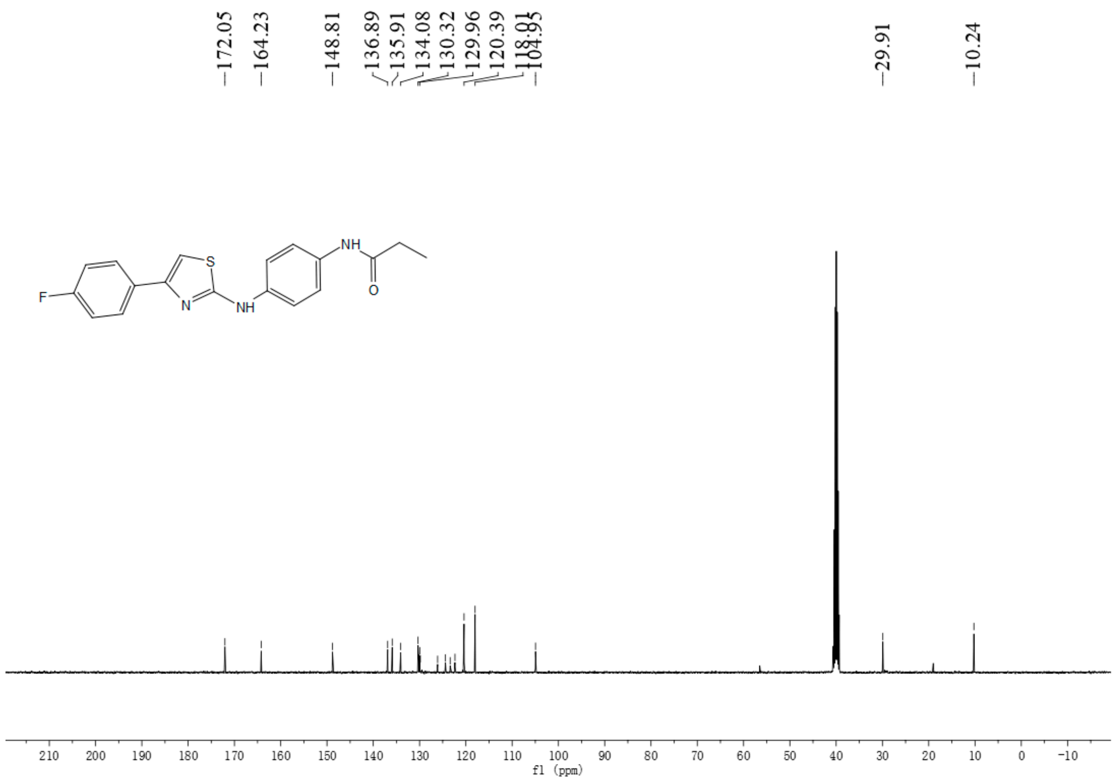

^{13}C NMR spectrum of compound **A**13

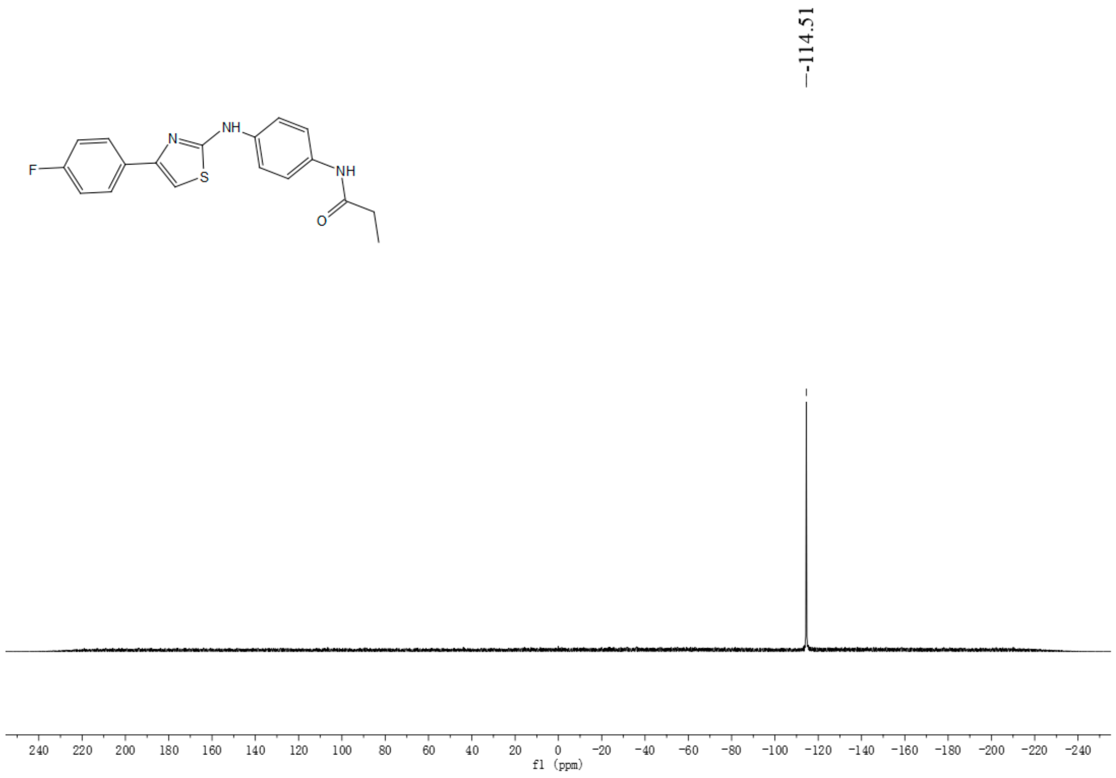

^{19}F NMR spectrum of compound **A**13

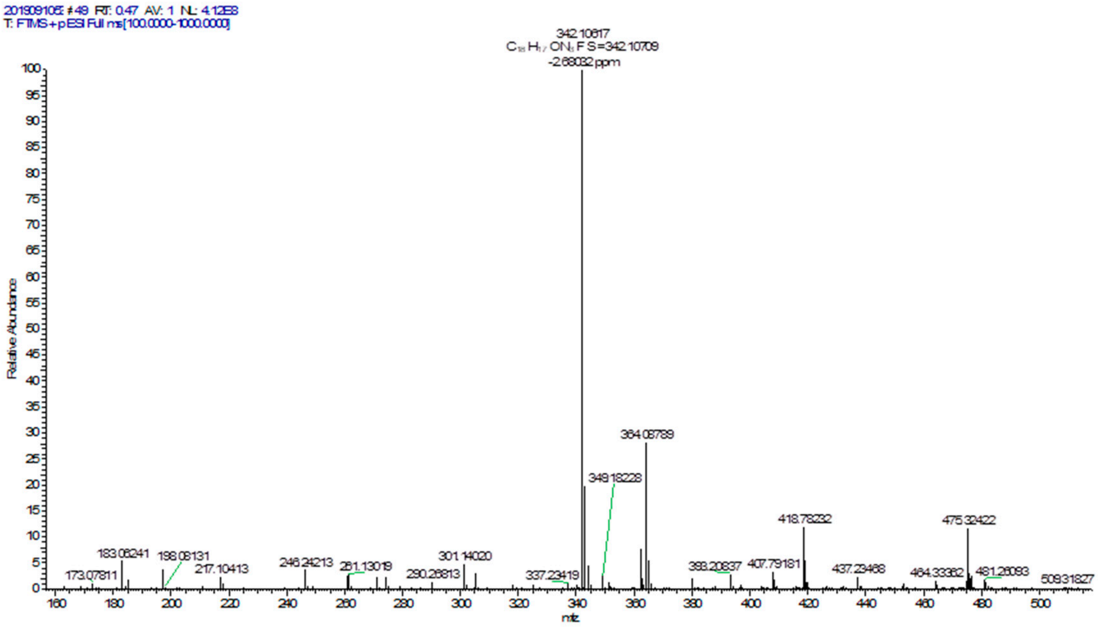

HRMS(ESI) of compound **A**13

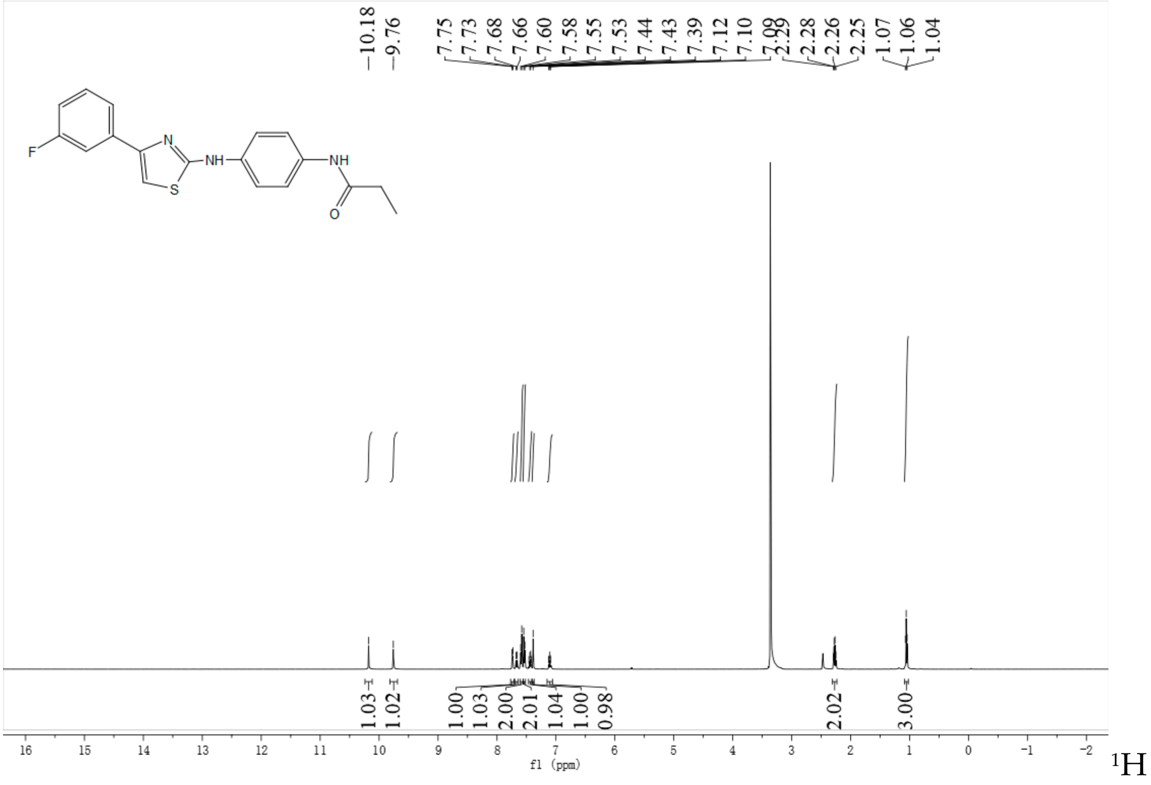

NMR spectrum of compound **A**14

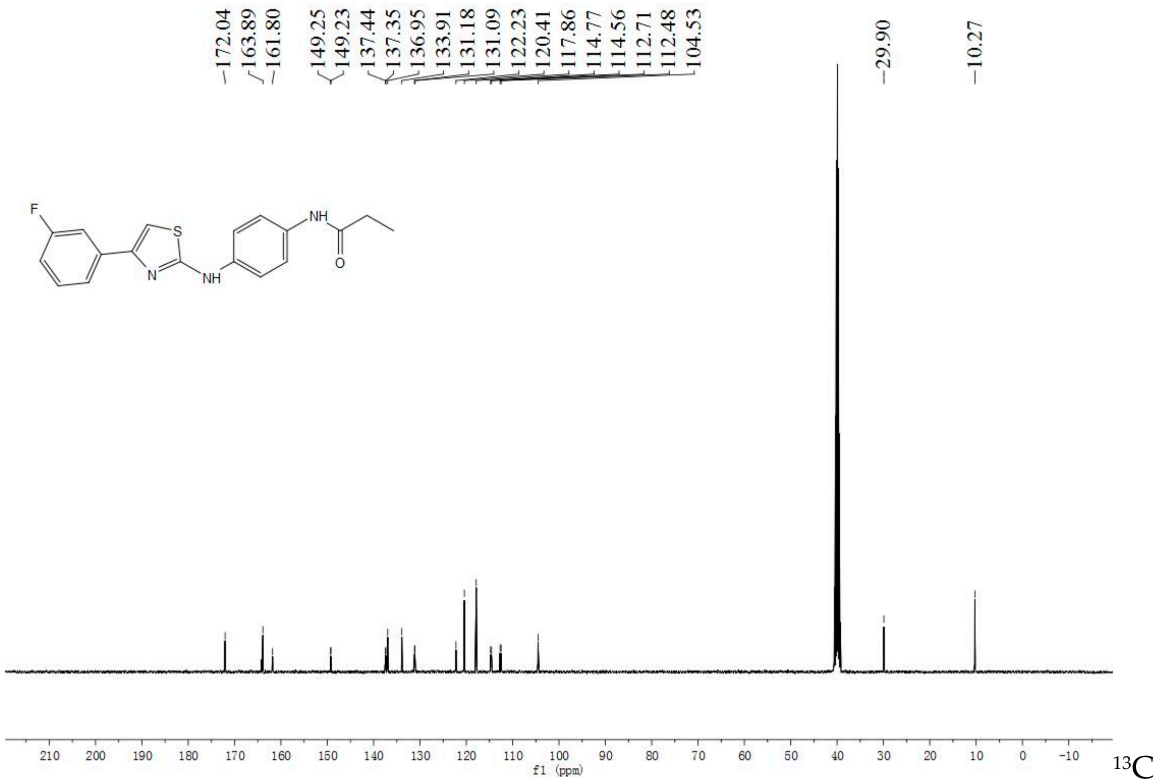

^{13}C NMR spectrum of compound **A**$_{14}$

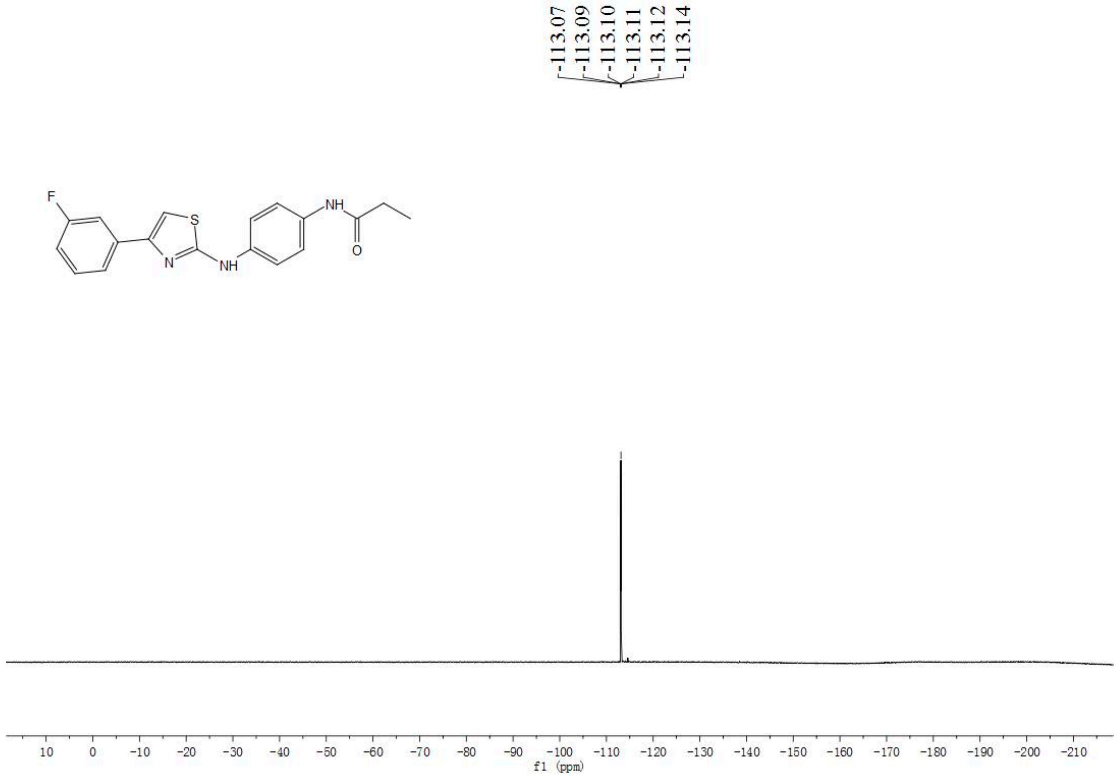

^{19}F NMR spectrum of compound **A**$_{14}$

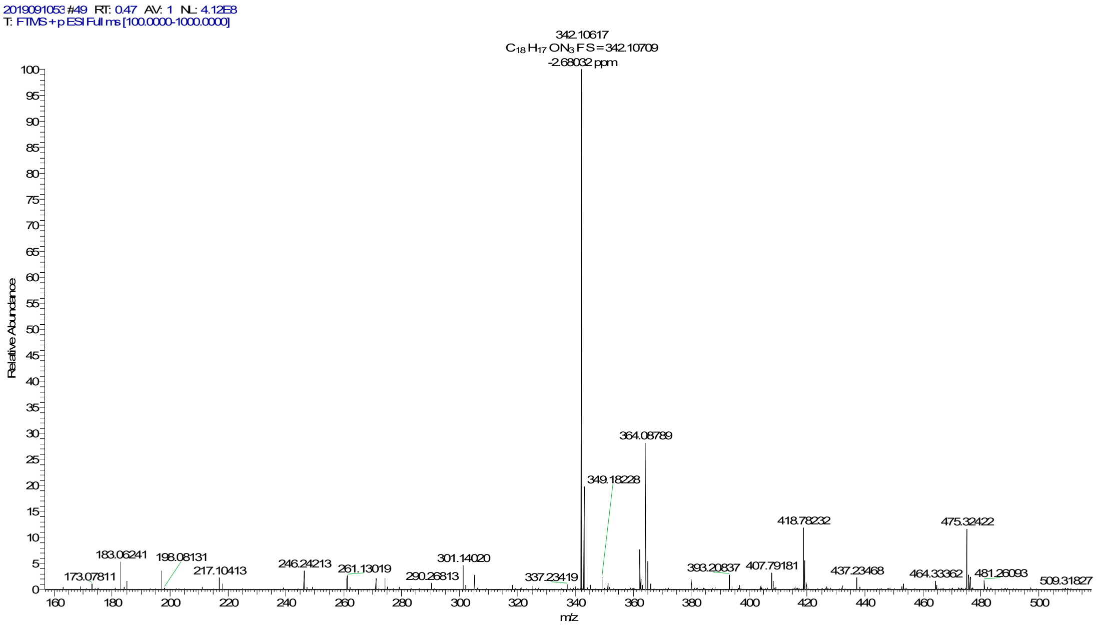

HRMS(ESI) of compound **A**14

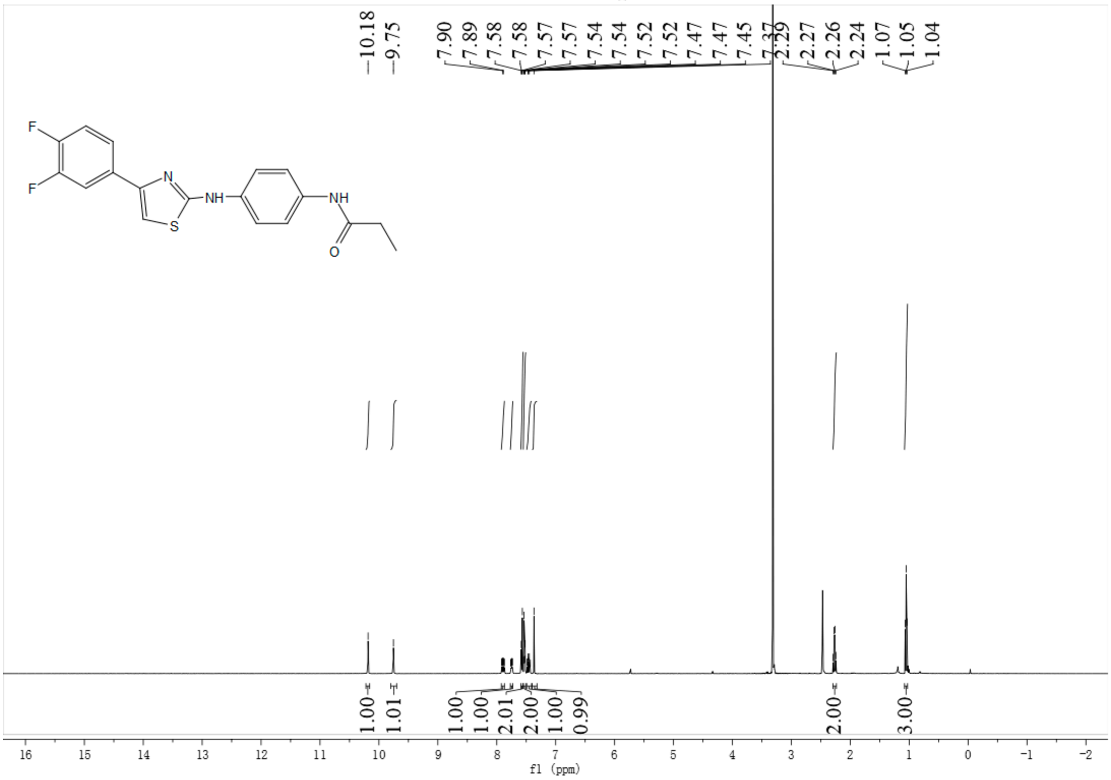

^{1}H NMR spectrum of compound **A**15

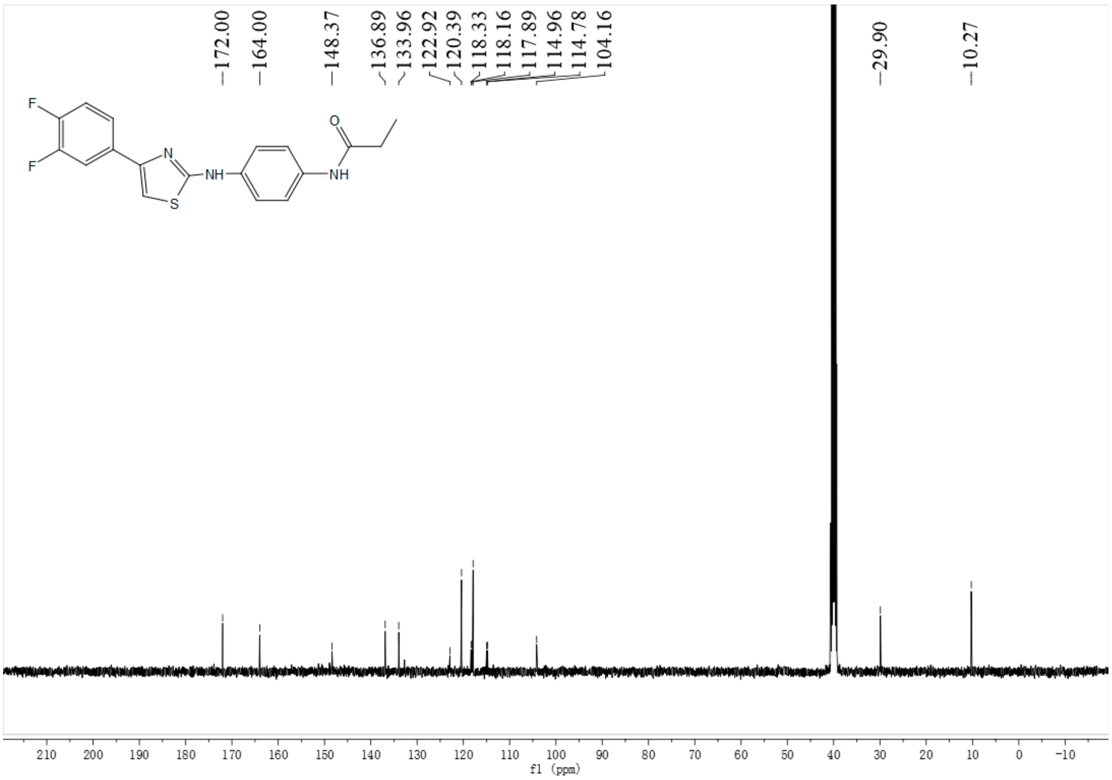

^{13}C NMR spectrum of compound **A**15

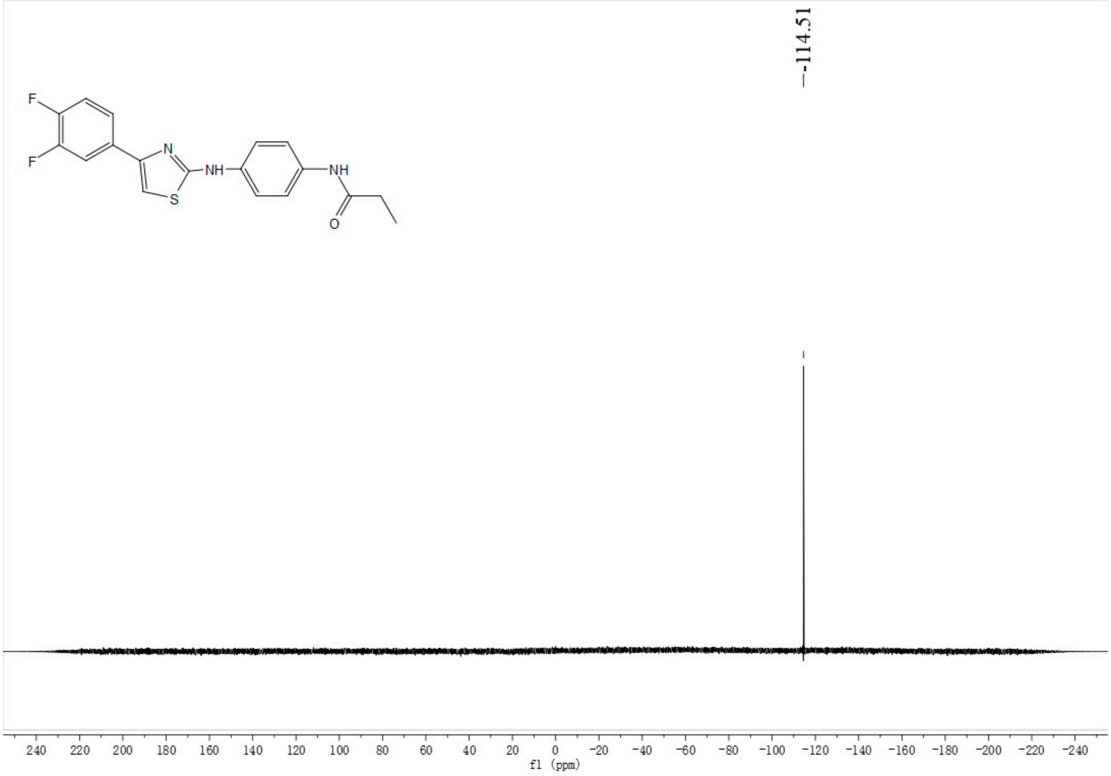

^{19}F NMR spectrum of compound **A**15

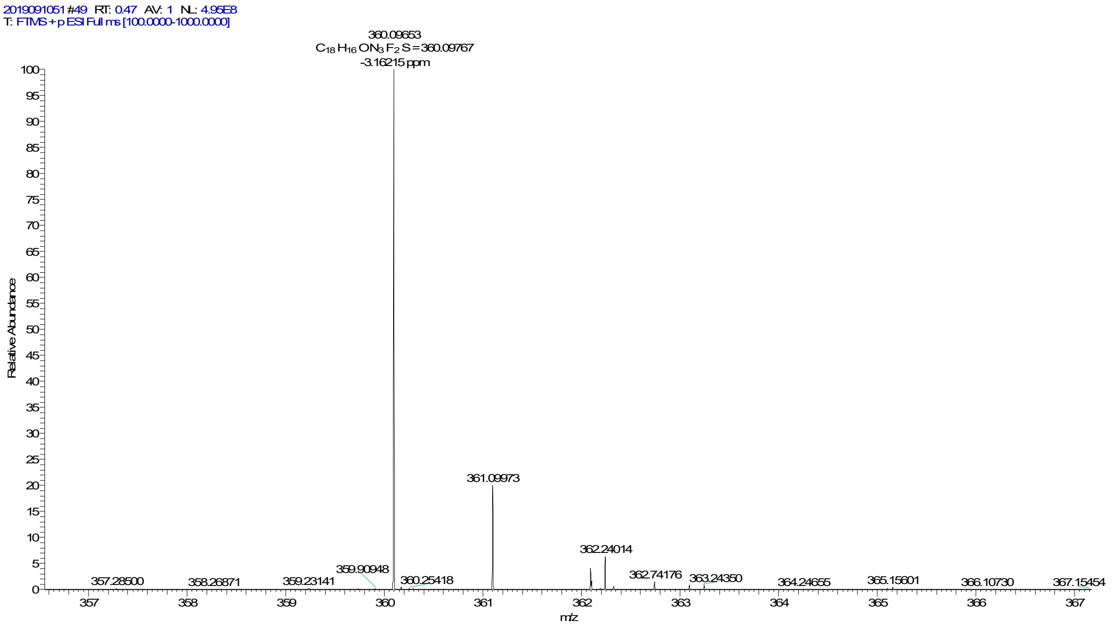

HRMS(ESI) of compound **A**15

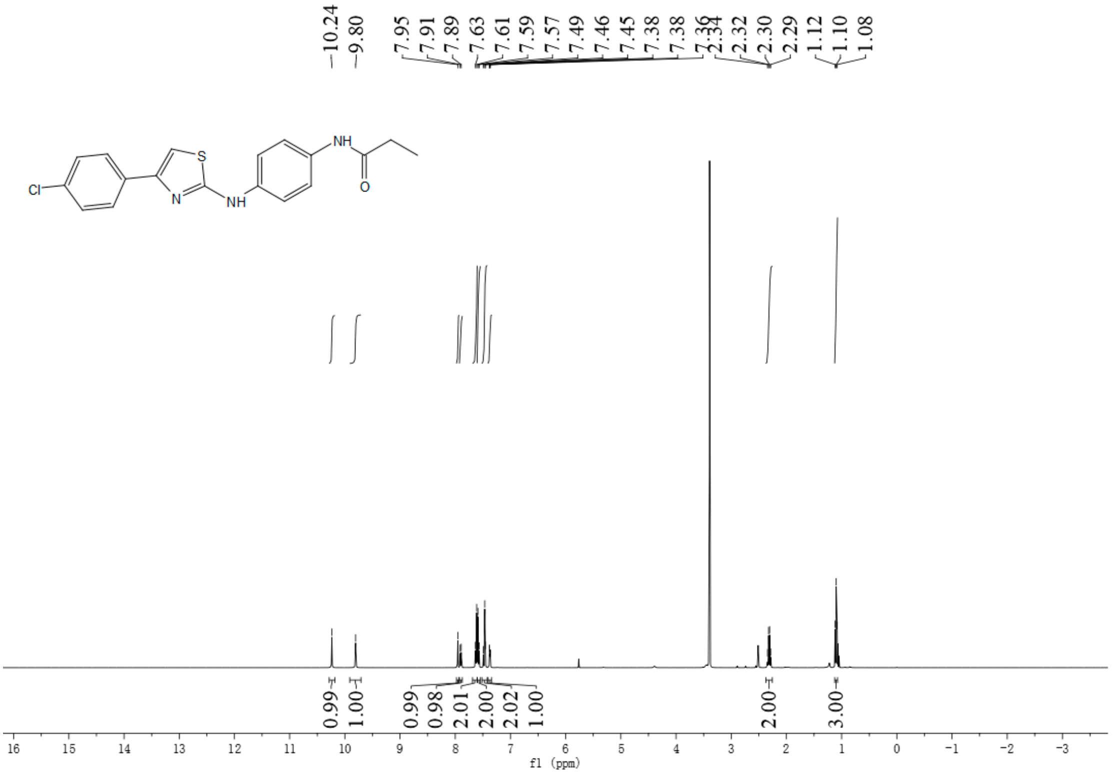

^{1}H NMR spectrum of compound A16

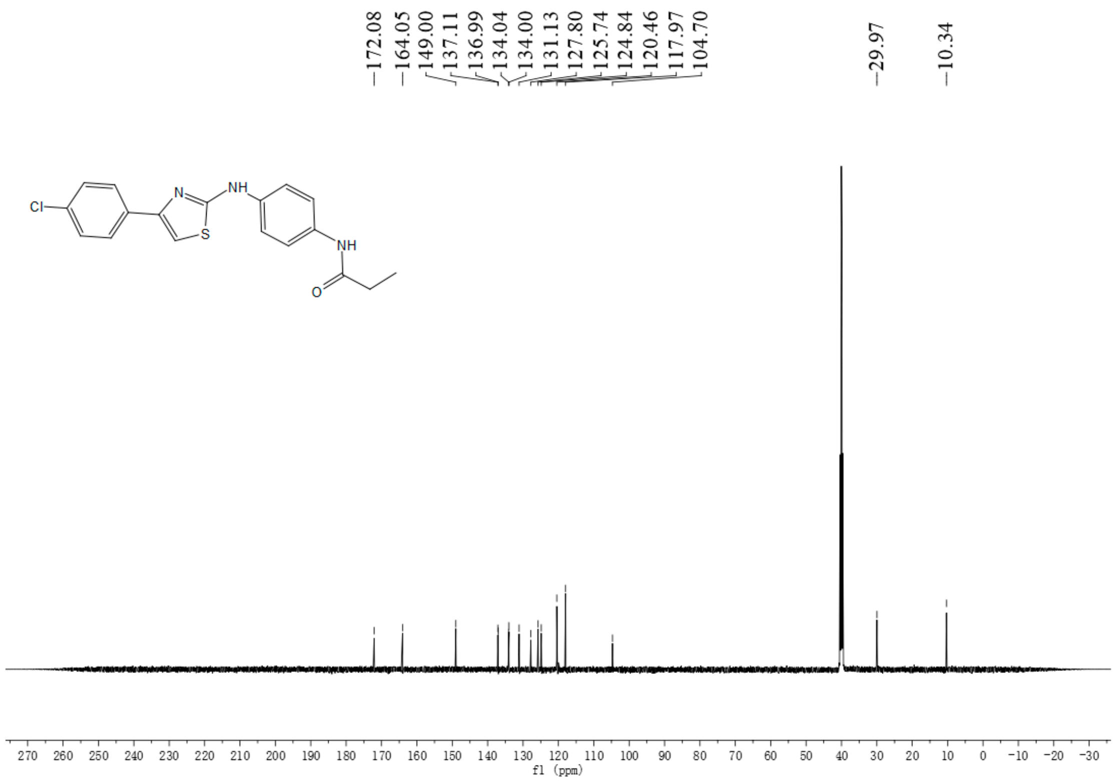

^{13}C NMR spectrum of compound **A**16

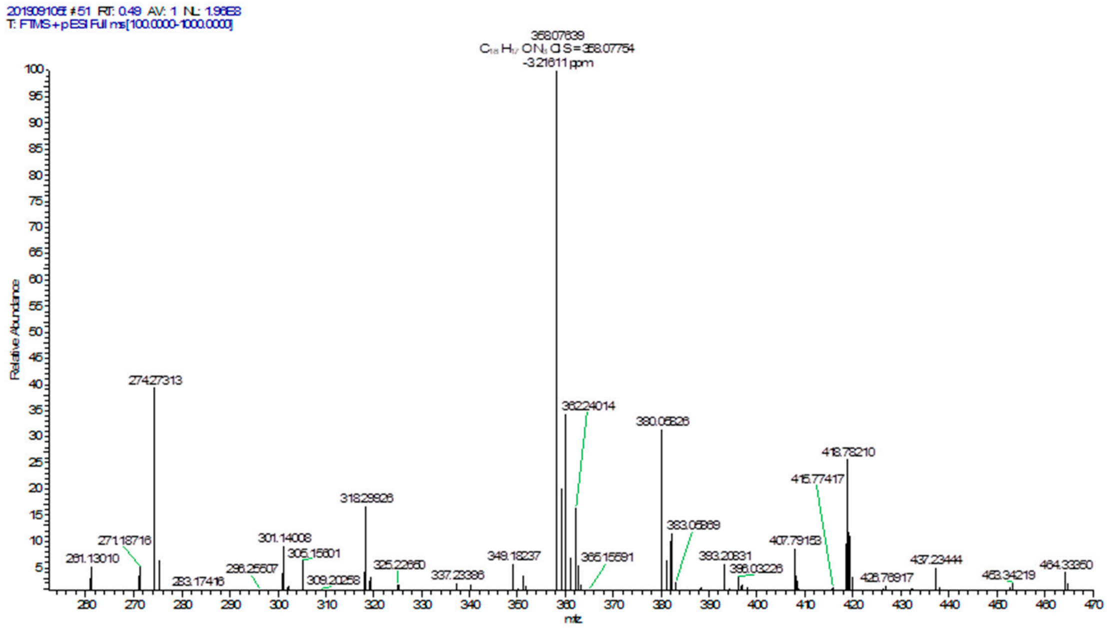

HRMS(ESI) of compound **A**16

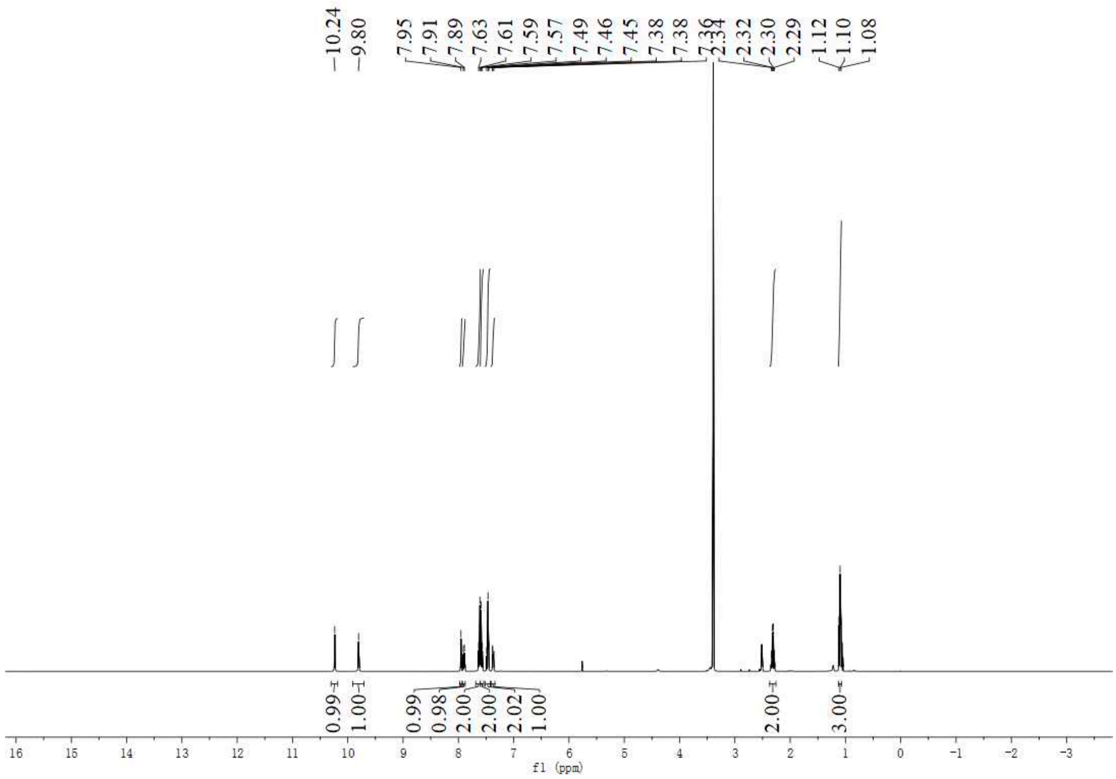

^{1}H NMR spectrum of compound **A**17

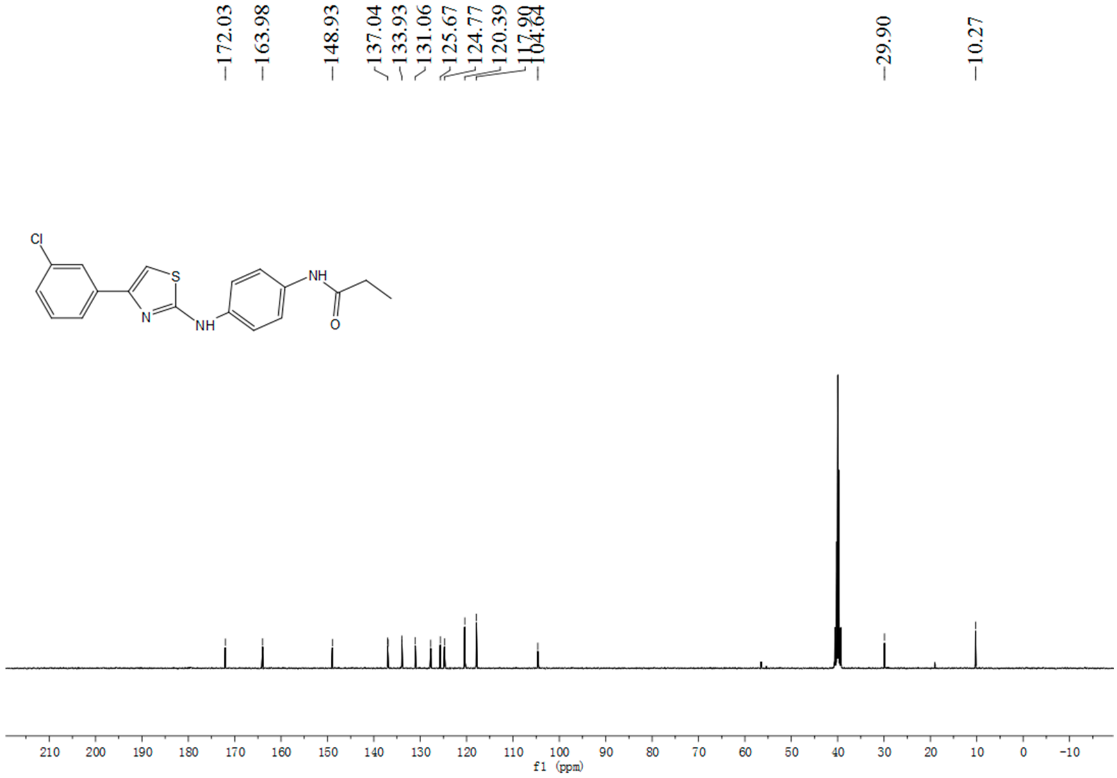

^{13}C NMR spectrum of compound **A**17

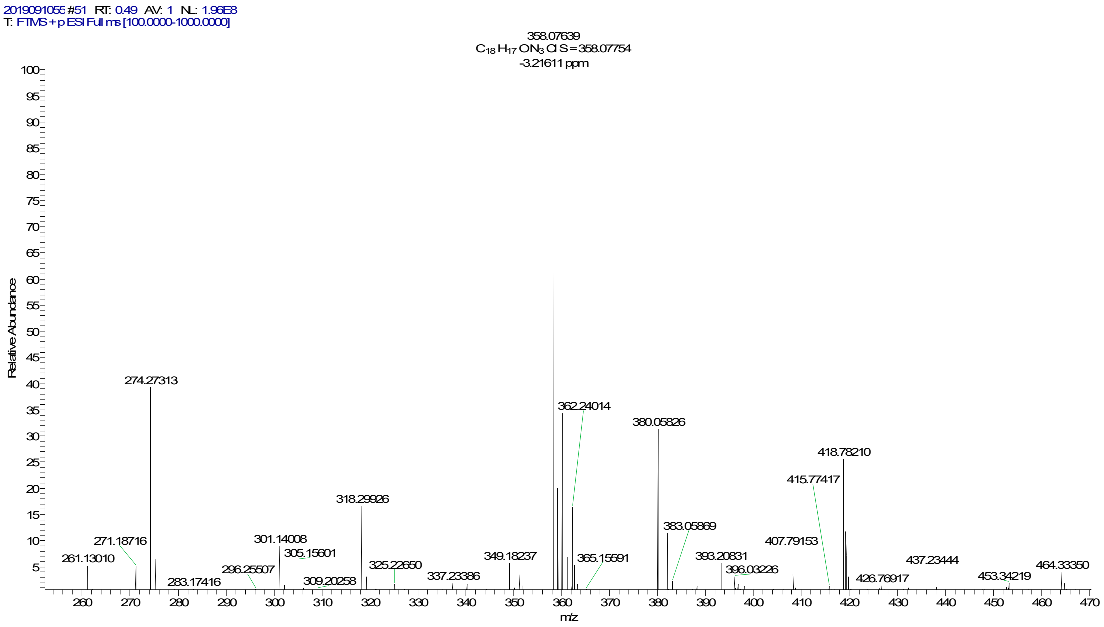

HRMS(ESI) of compound **A**17

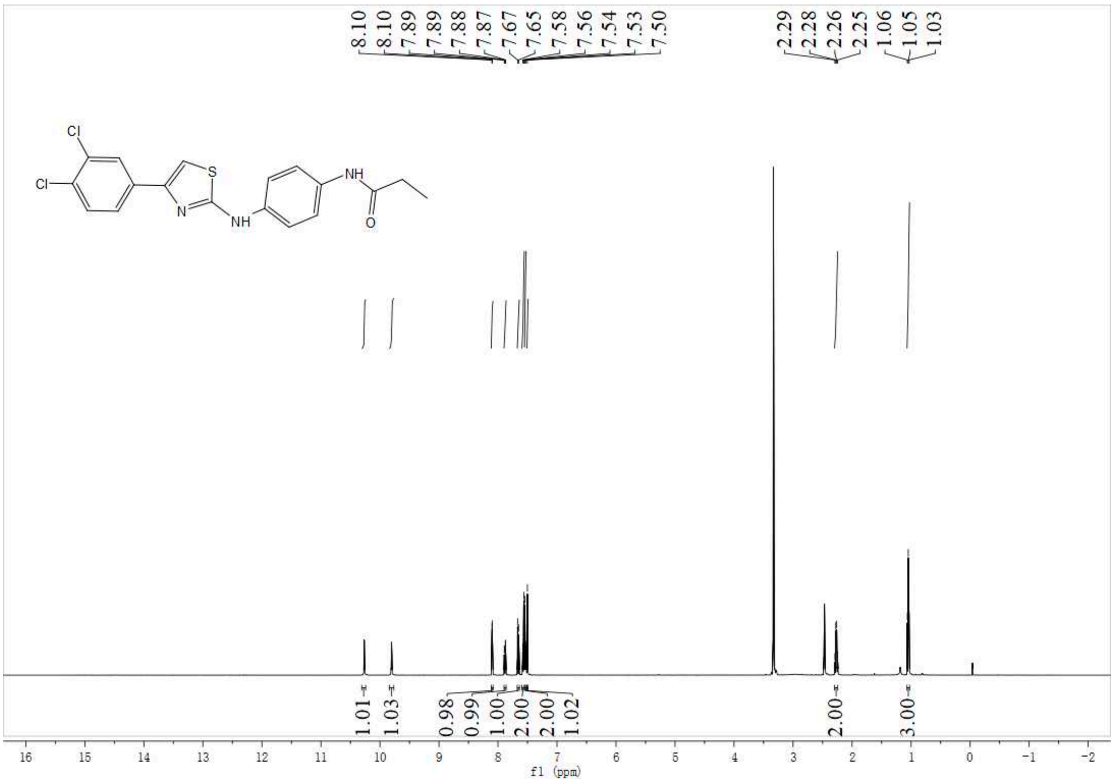

^{1}H NMR spectrum of compound **A**18

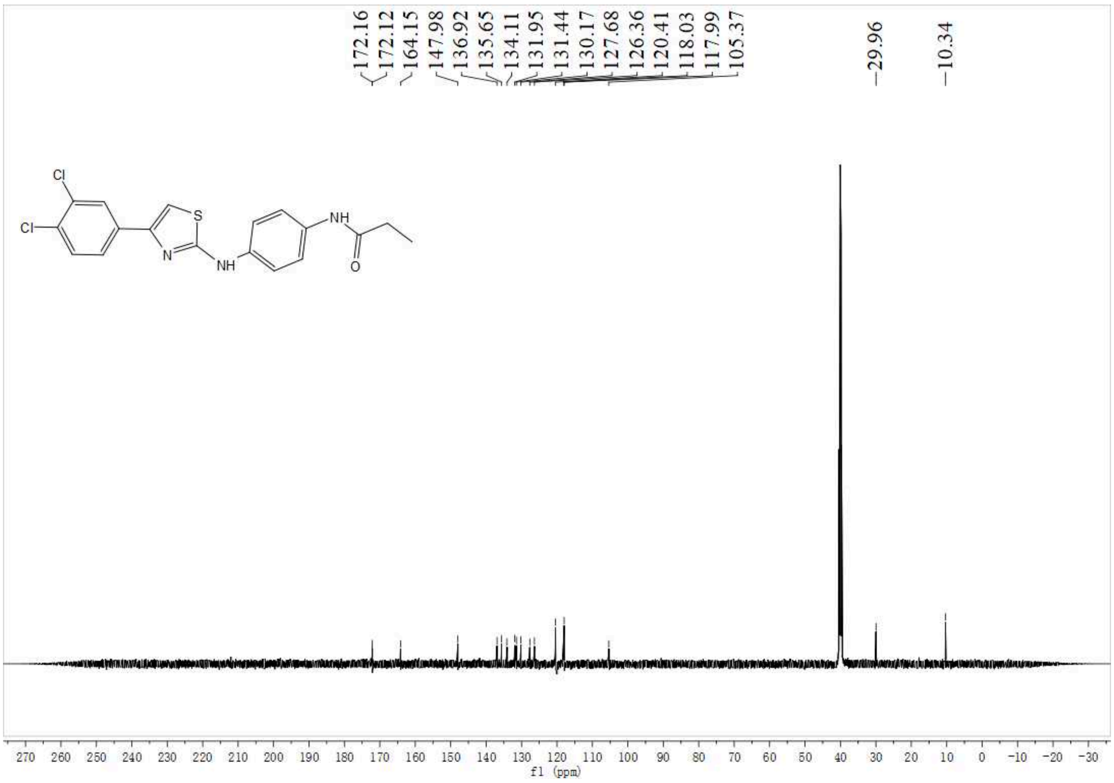

^{13}C NMR spectrum of compound **A**$_{18}$

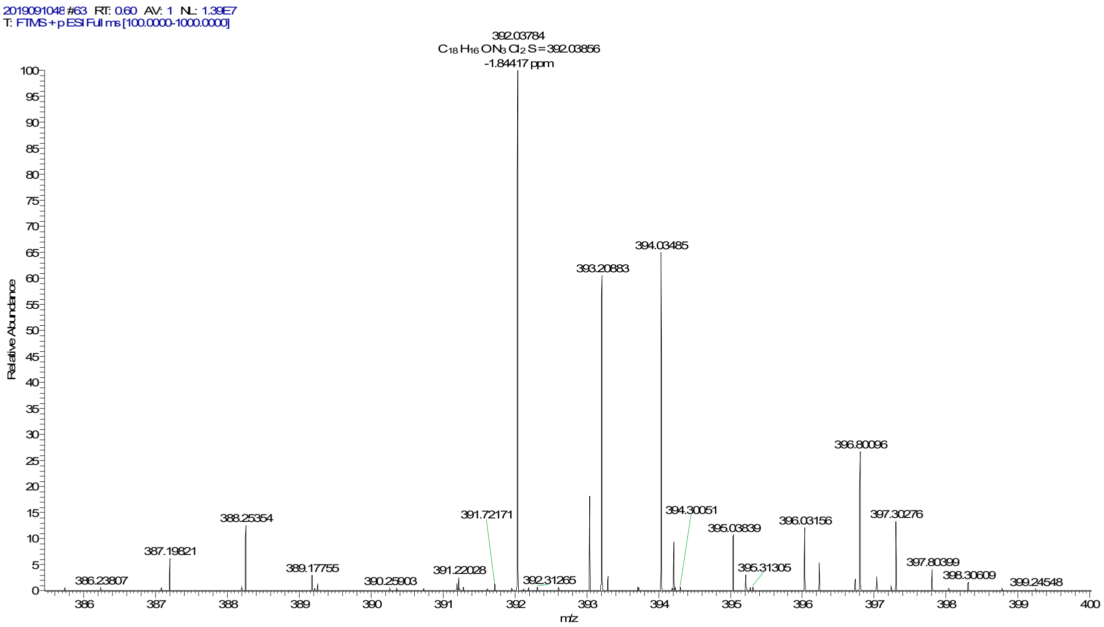

HRMS(ESI) of compound **A**$_{18}$

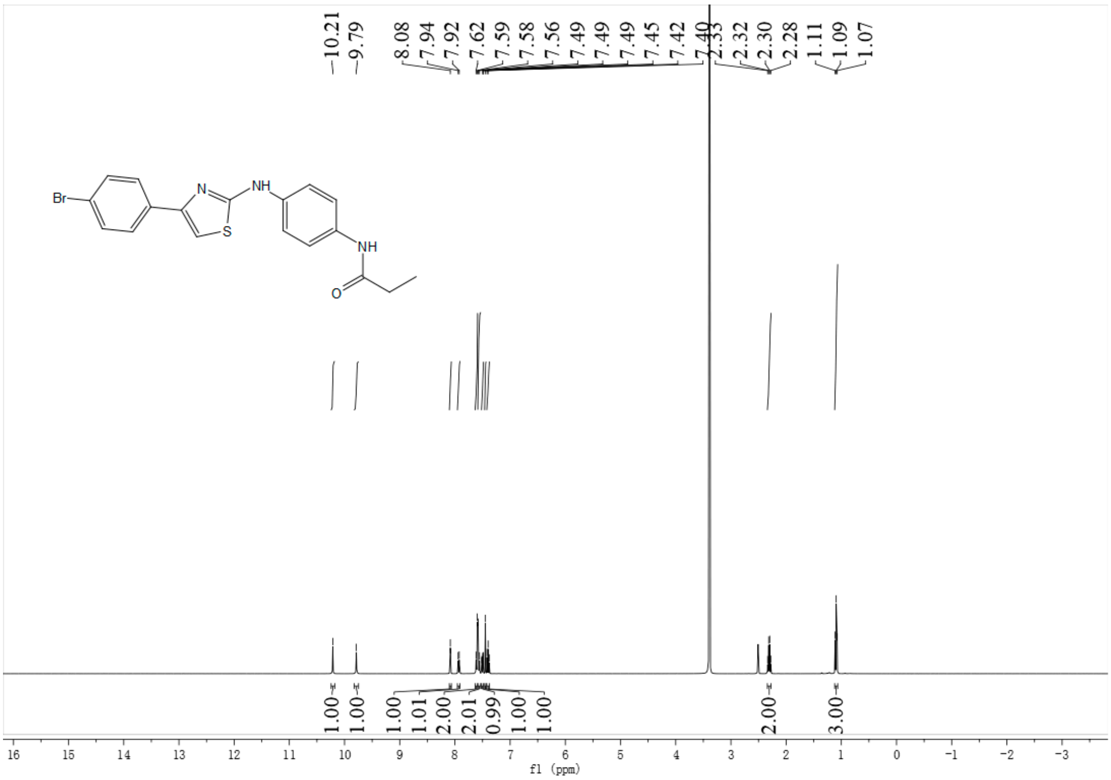

^{1}H NMR spectrum of compound **A**19

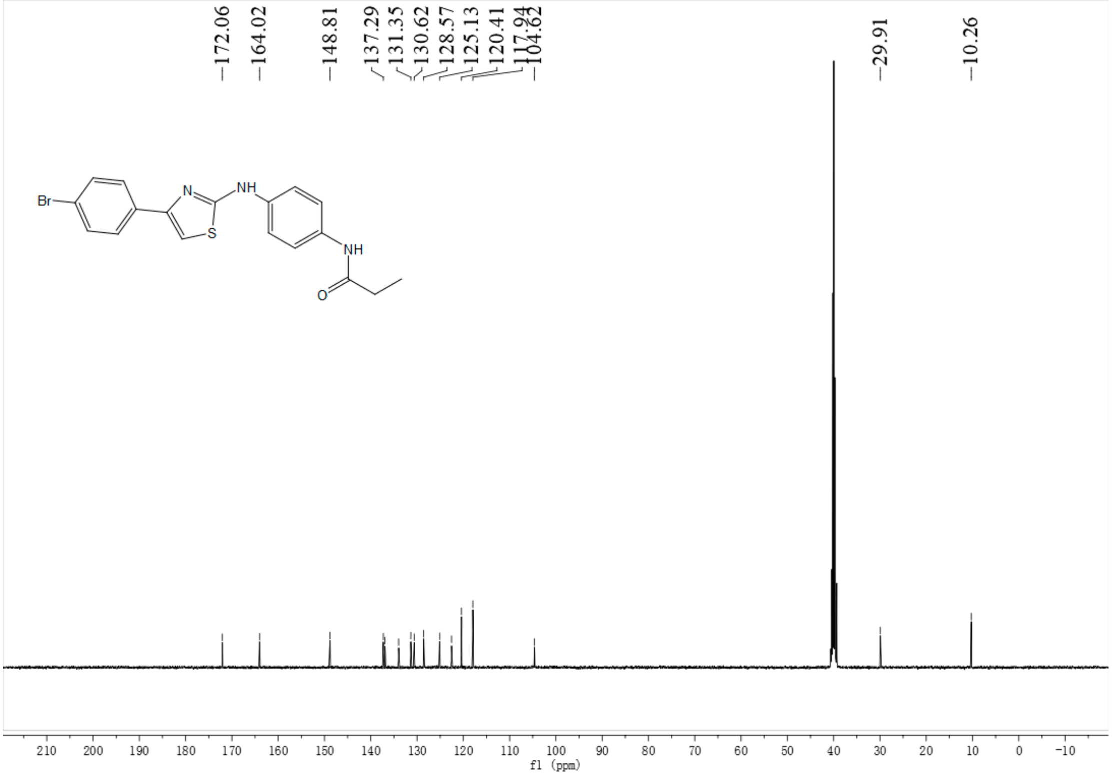

^{13}C NMR spectrum of compound **A**19

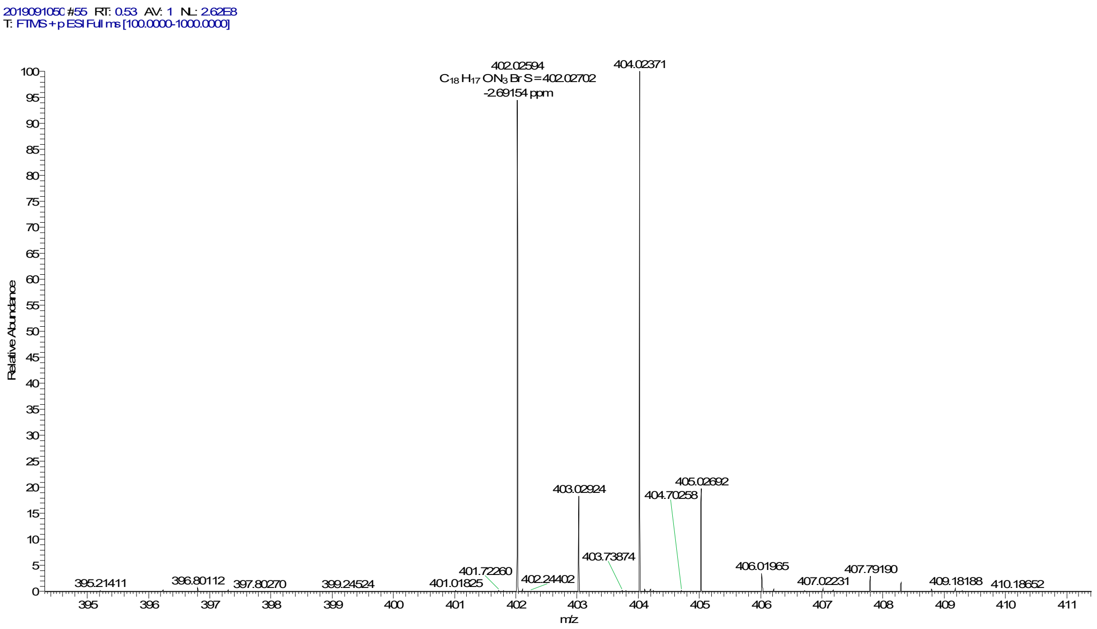

HRMS(ESI) of compound **A**19

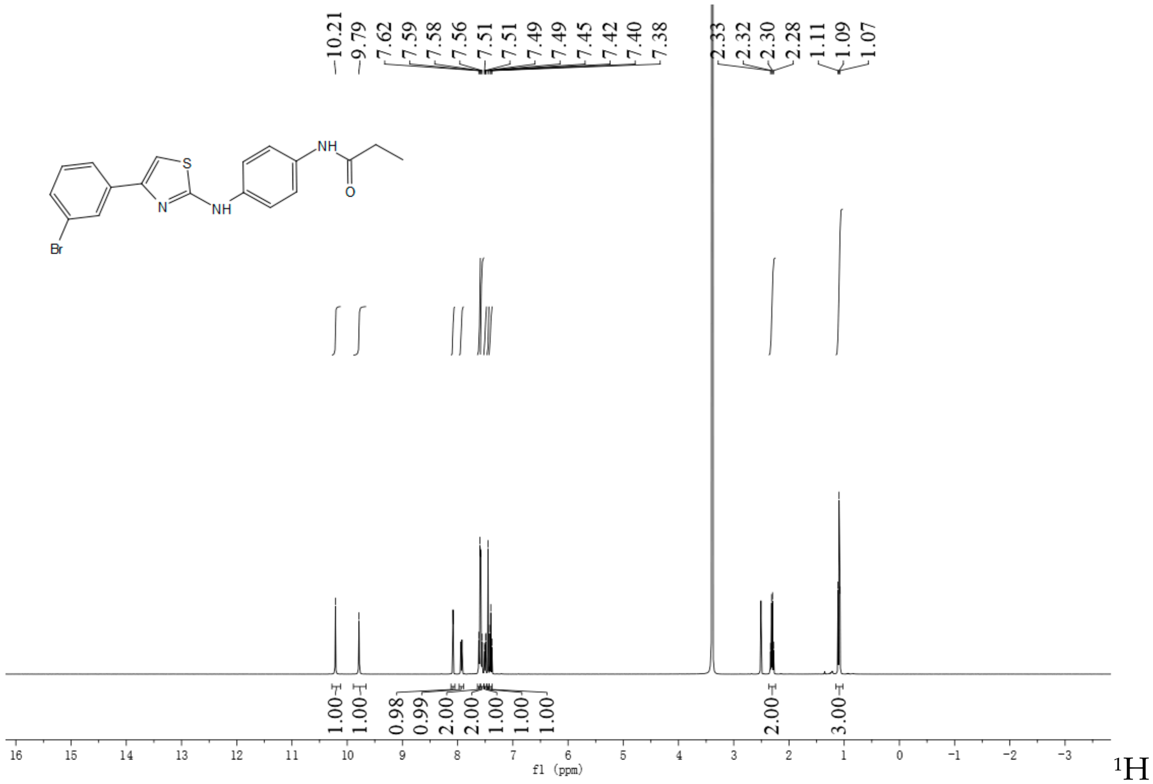

^{1}H NMR spectrum of compound **A**20

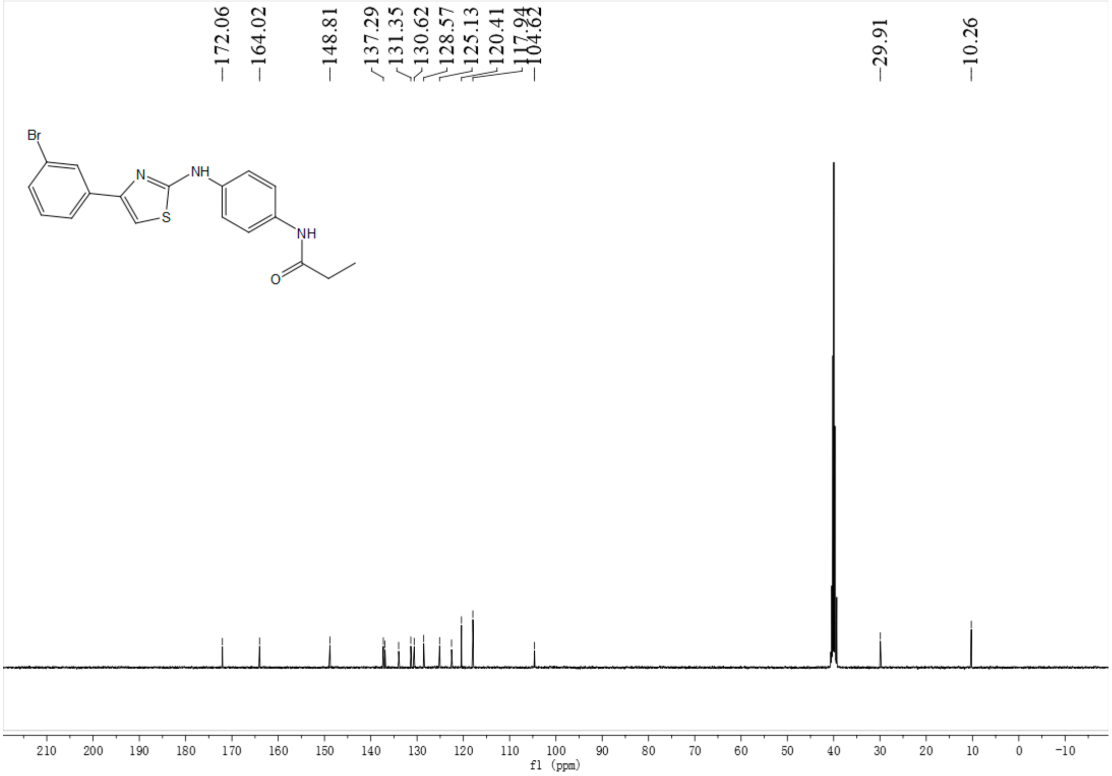

^{13}C NMR spectrum of compound **A**20

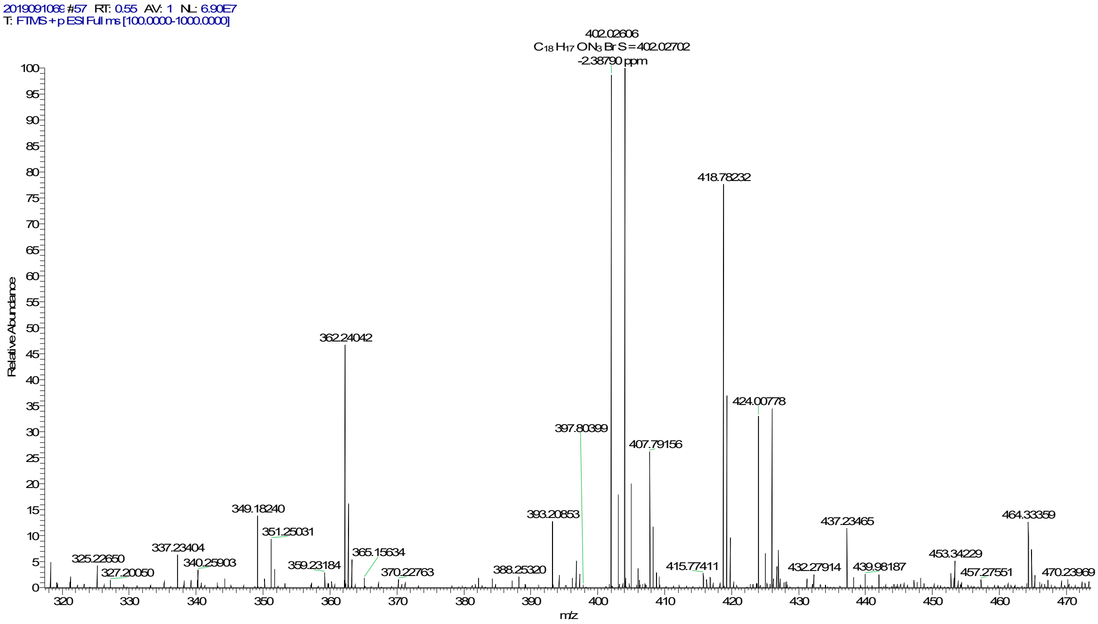

HRMS(ESI) of compound **A**20

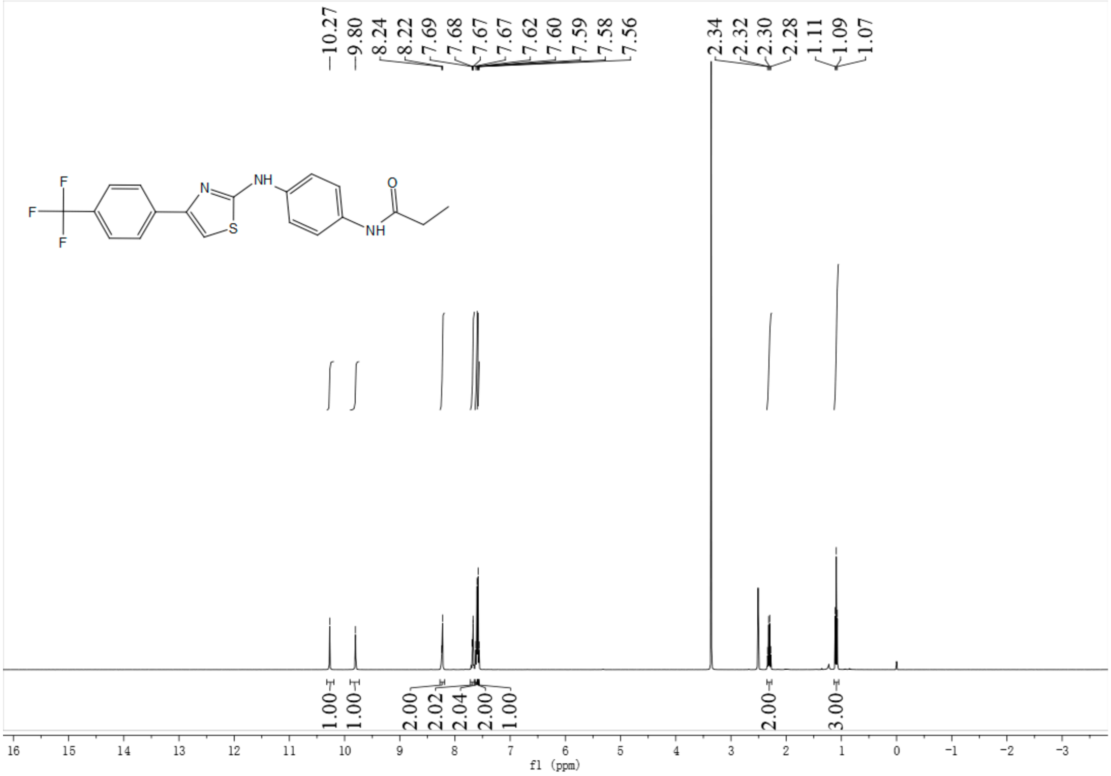

^{1}H NMR spectrum of compound **A**21

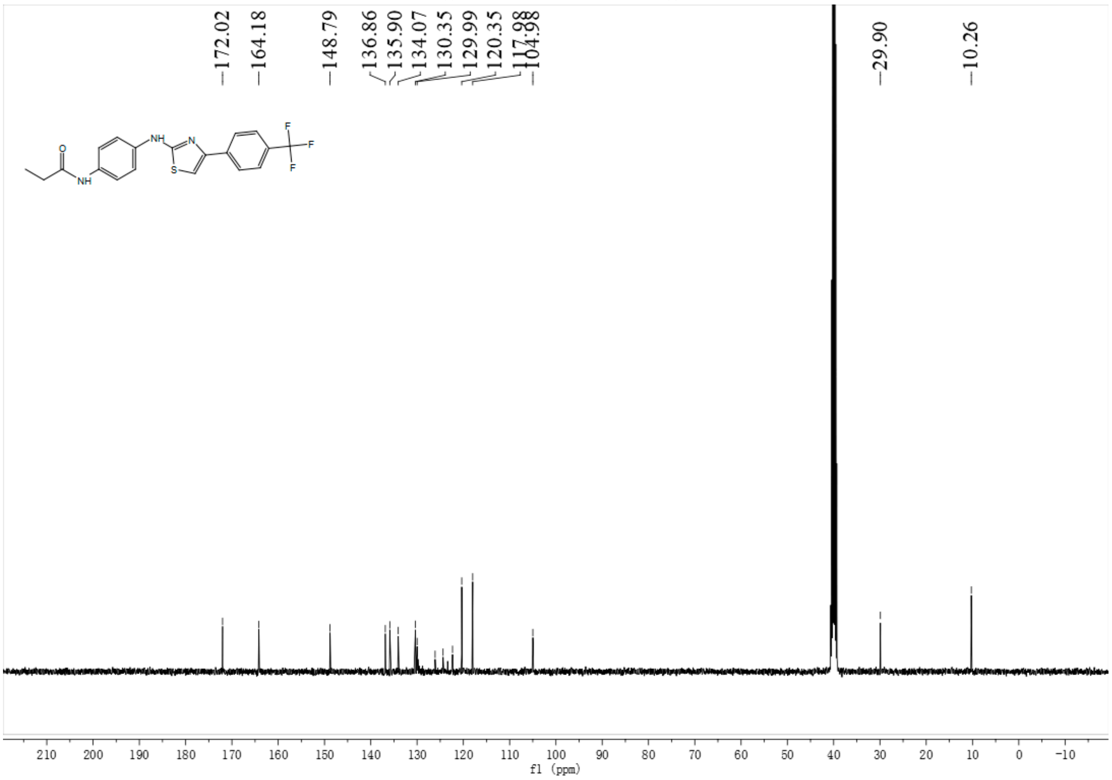

^{13}C NMR spectrum of compound **A**21

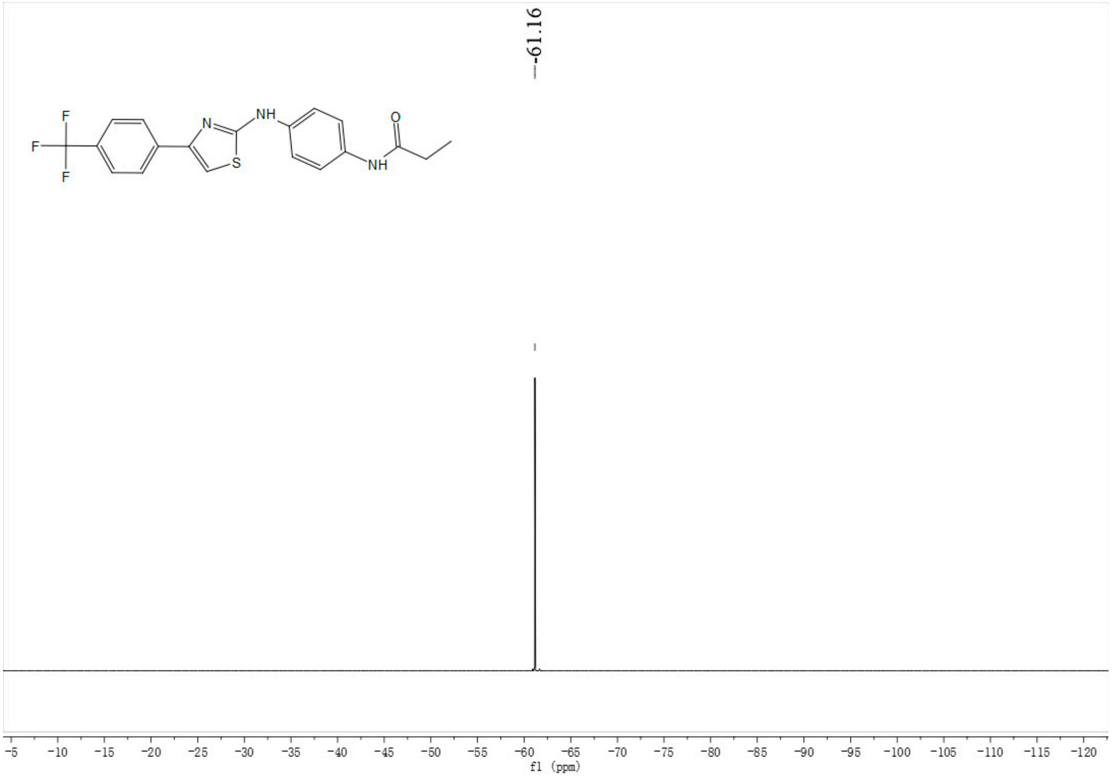

^{19}F NMR spectrum of compound **A**21

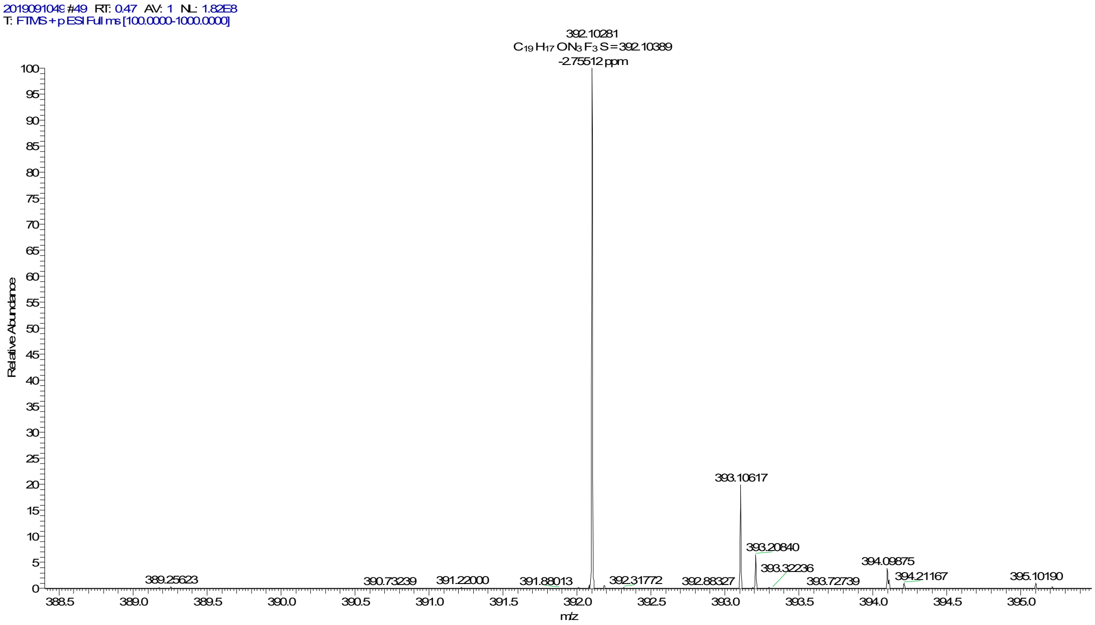

HRMS(ESI) of compound **A**21

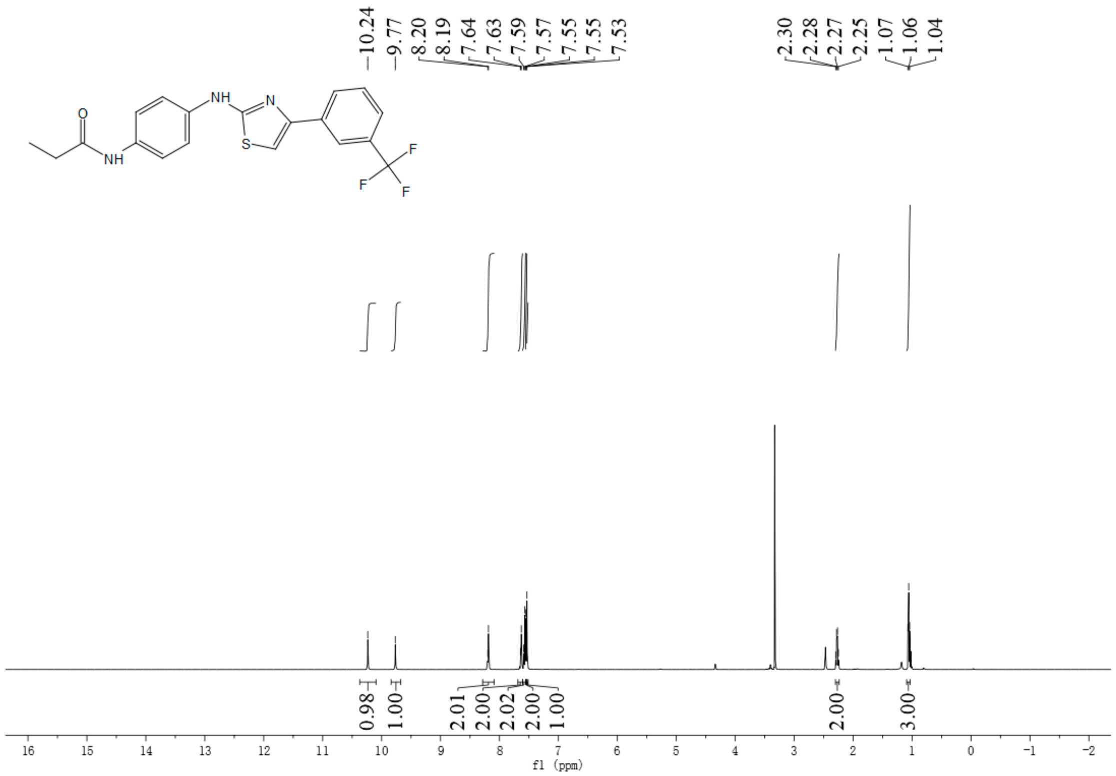

^{1}H NMR spectrum of compound **A**$_{22}$

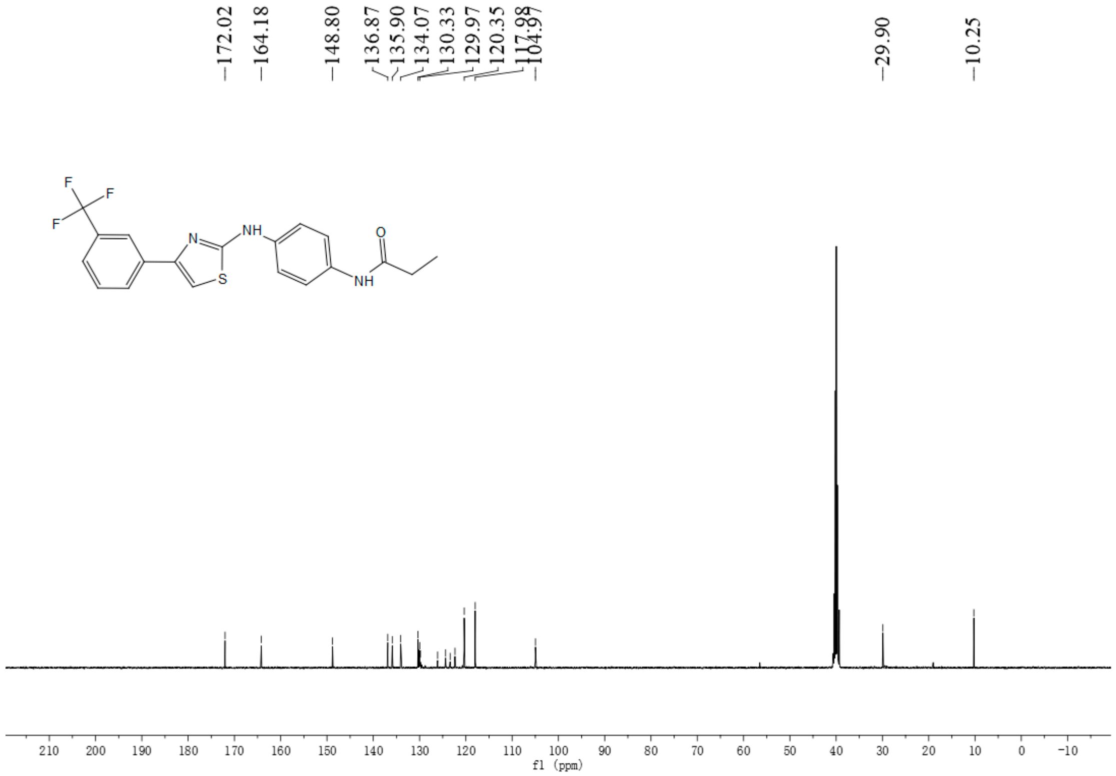

^{13}C NMR spectrum of compound **A**$_{22}$

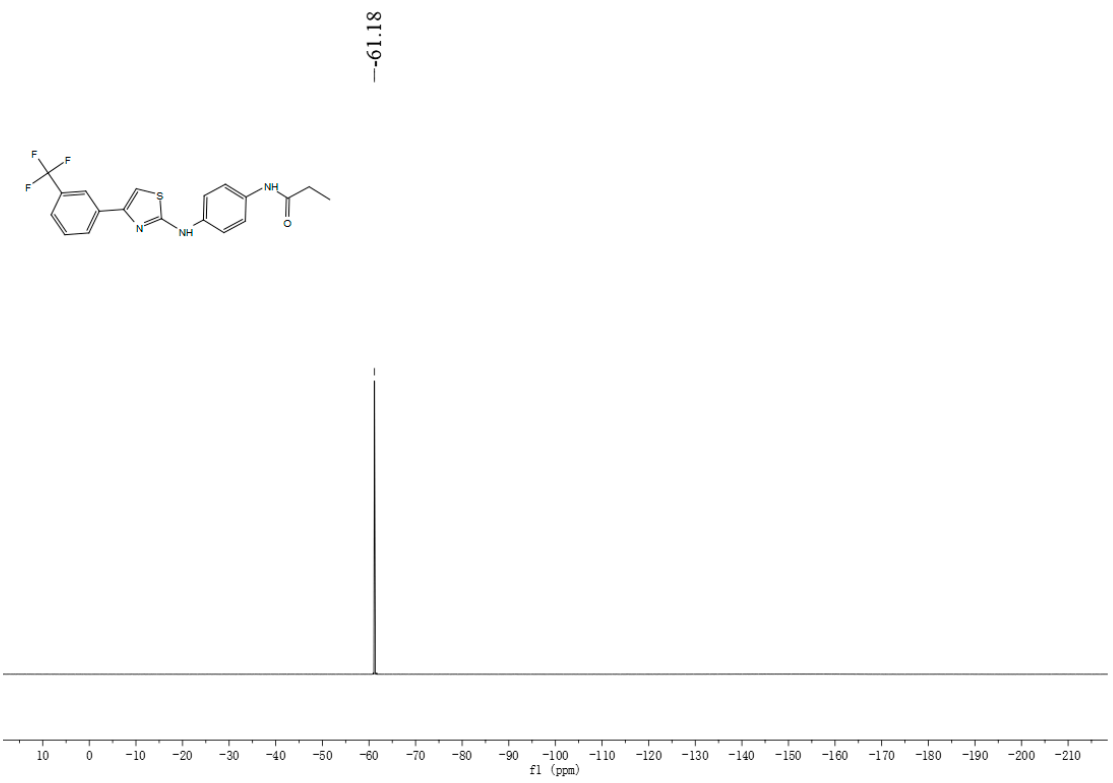

^{19}F NMR spectrum of compound **A**$_{22}$

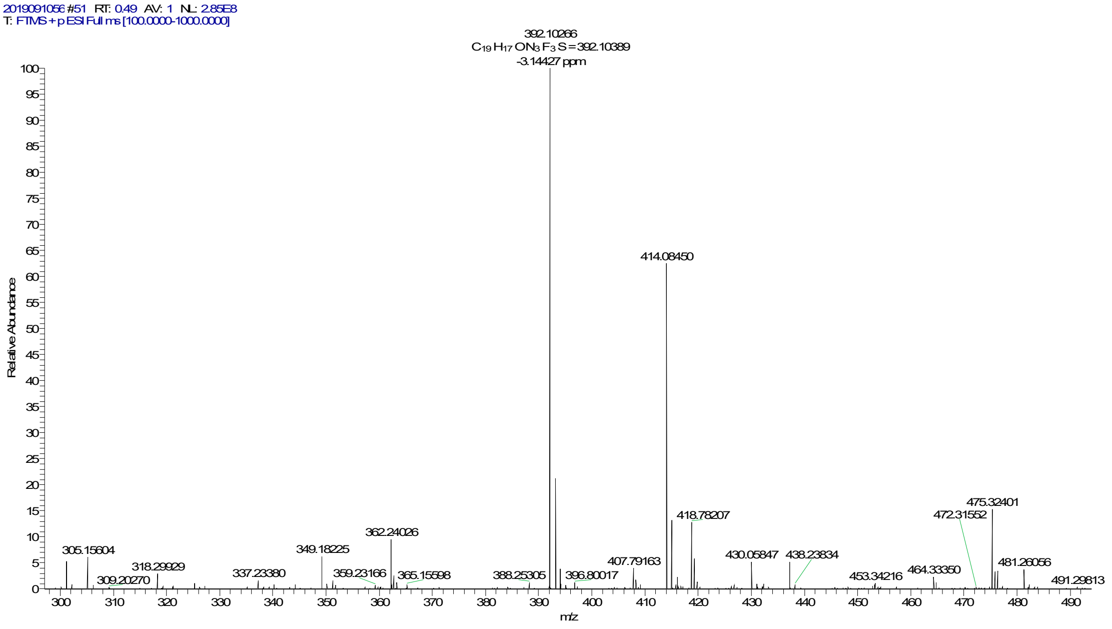

HRMS(ESI) of compound **A**$_{22}$

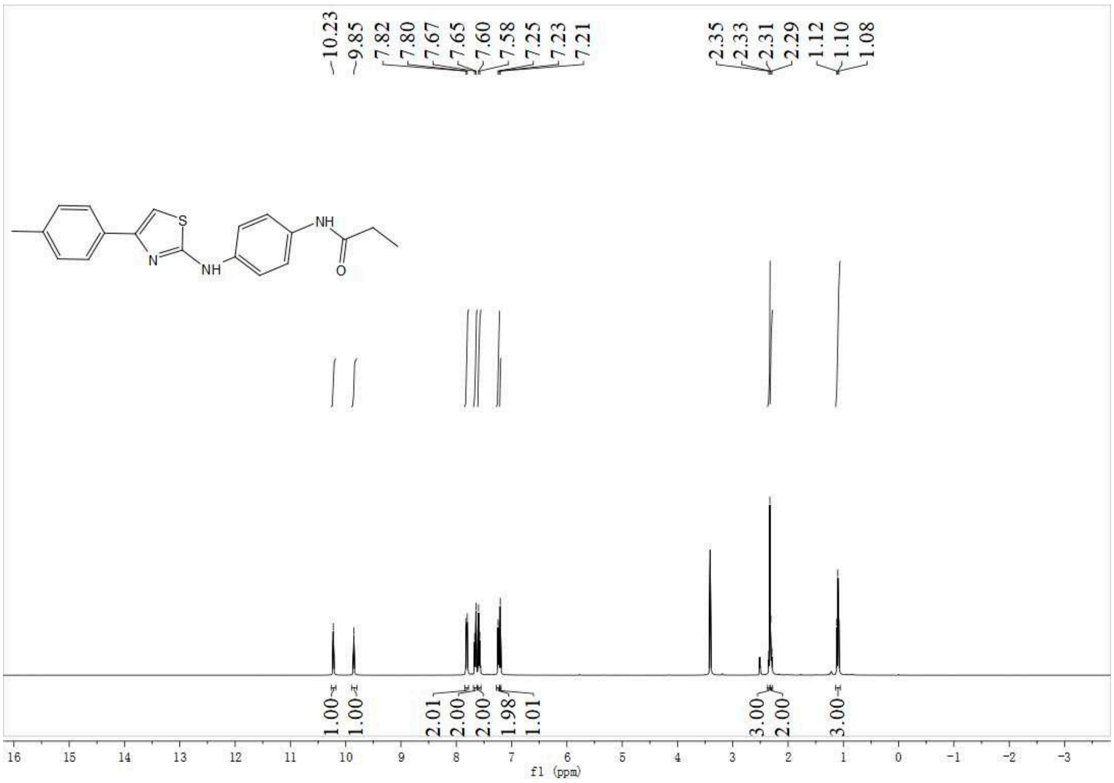

^{1}H NMR spectrum of compound **A**23

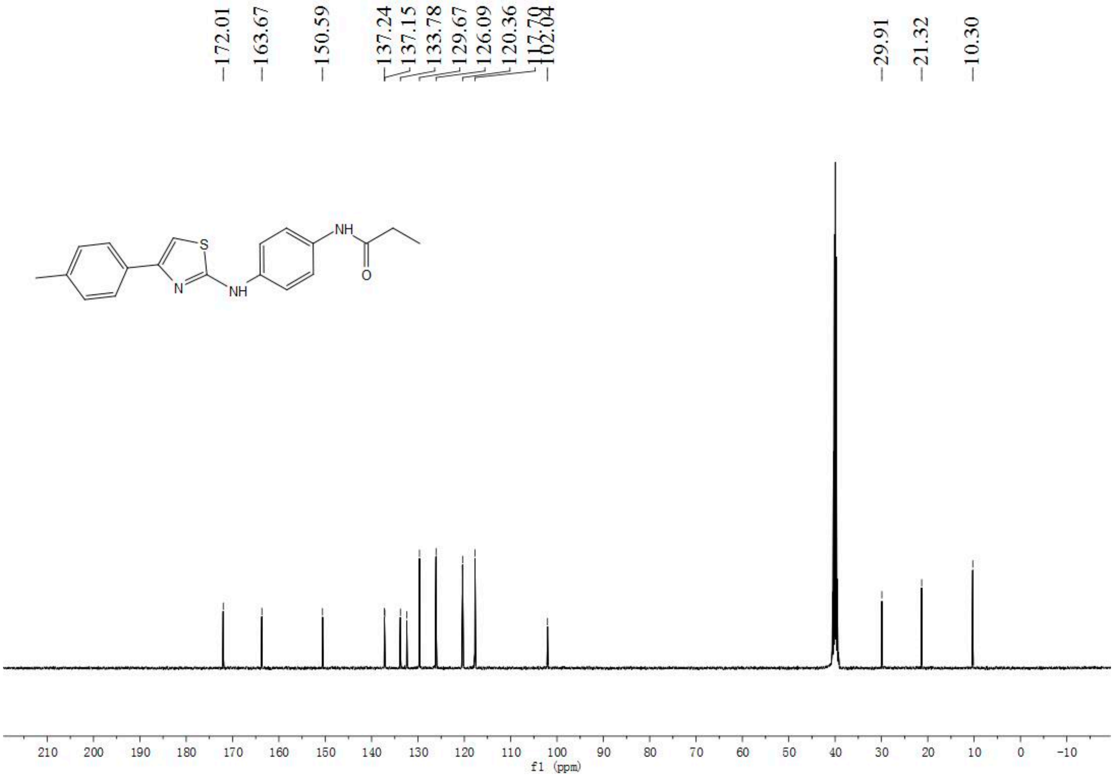

^{13}C NMR spectrum of compound **A**23

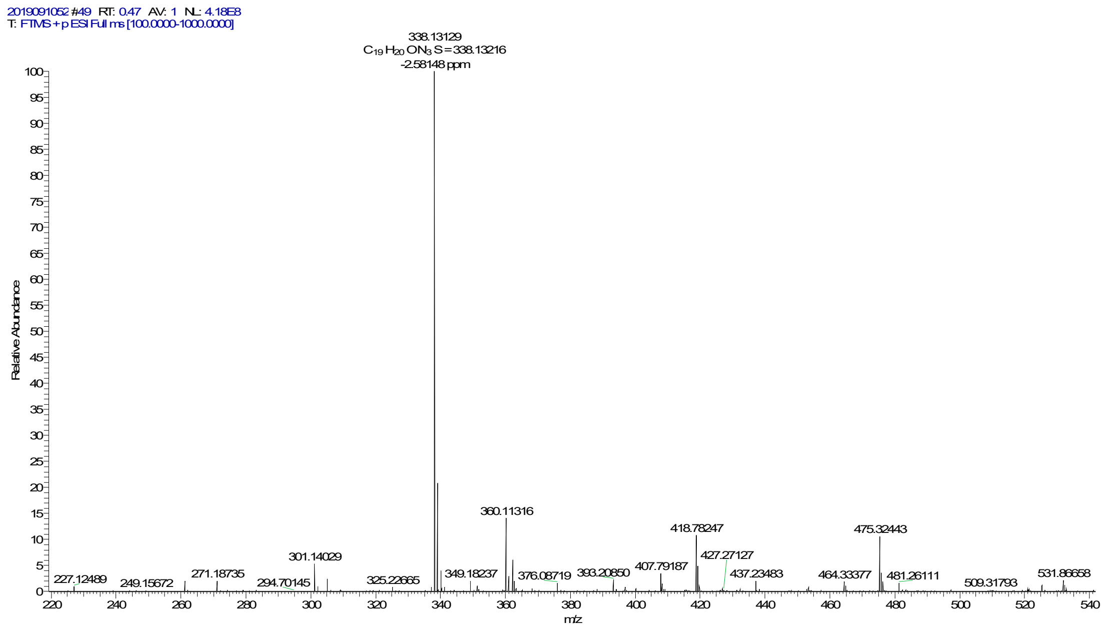

HRMS(ESI) of compound **A**23

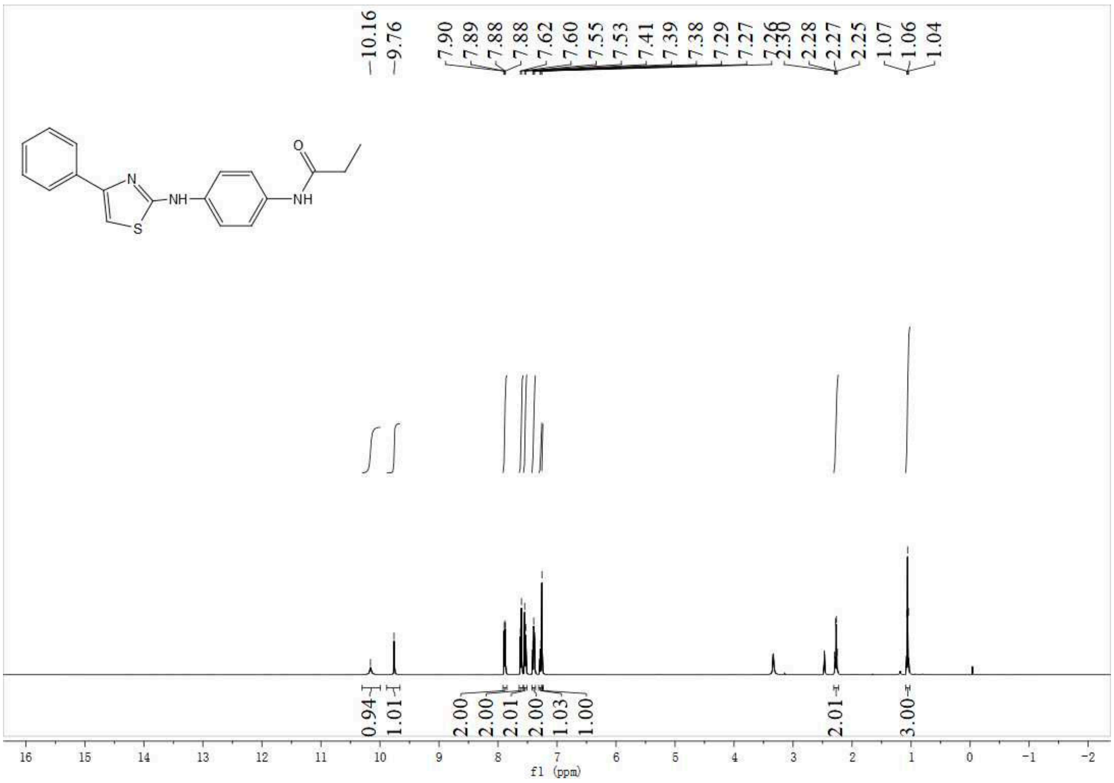

^{1}H NMR spectrum of compound **A**24

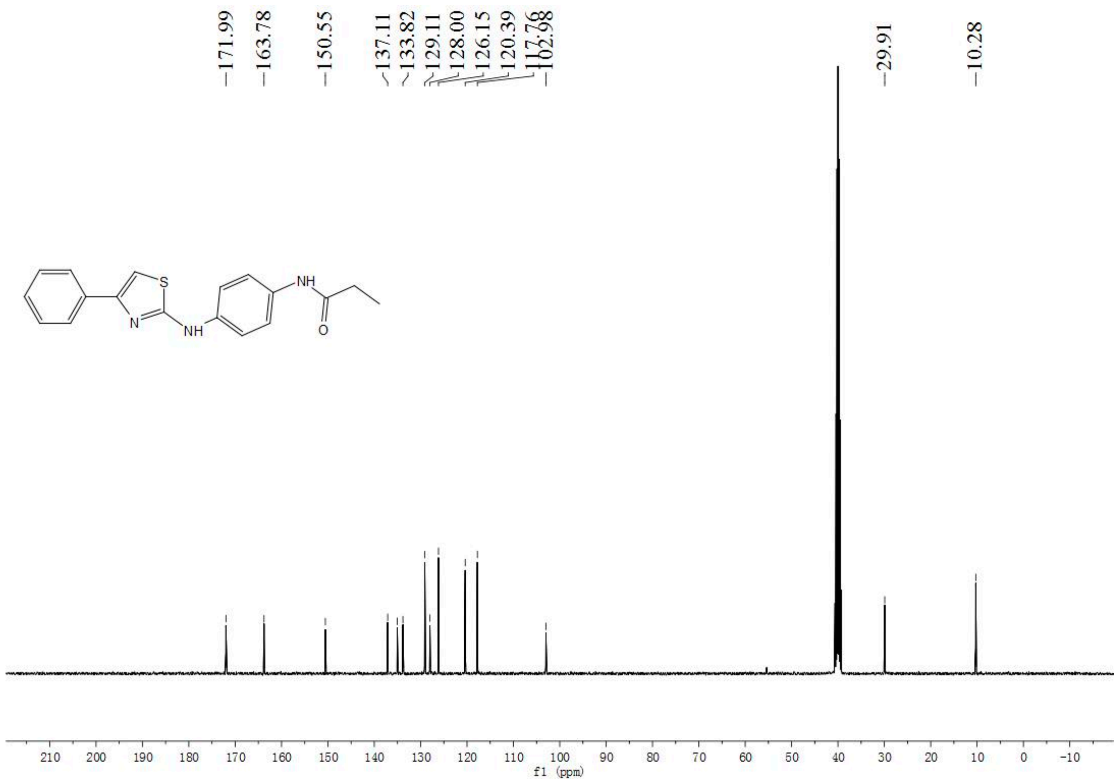

^{13}C NMR spectrum of compound **A**24

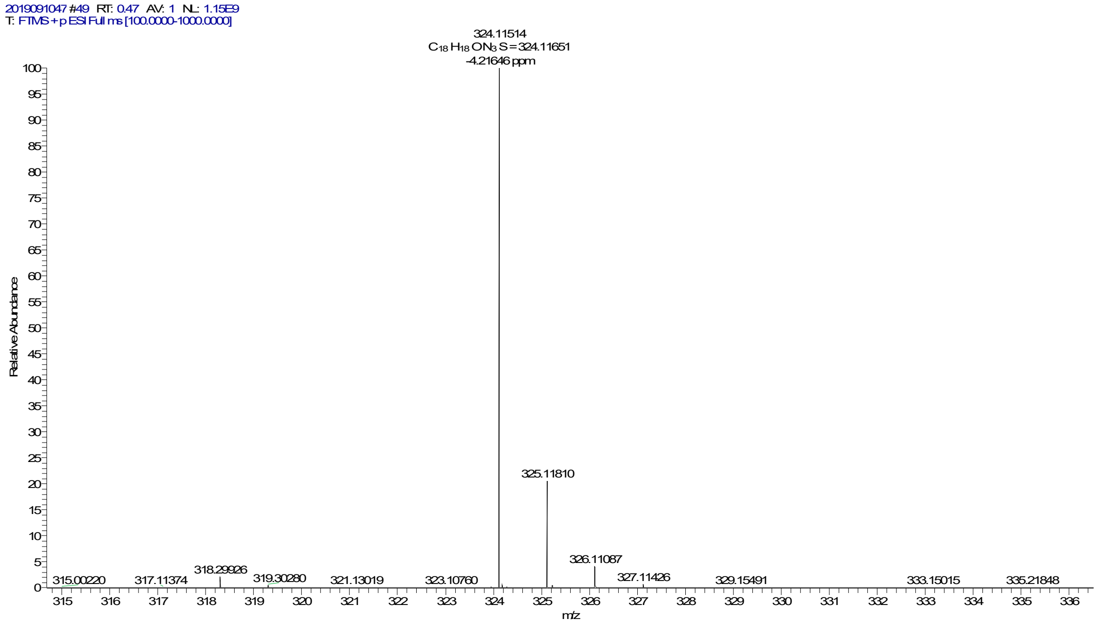

HRMS(ESI) of compound **A**24

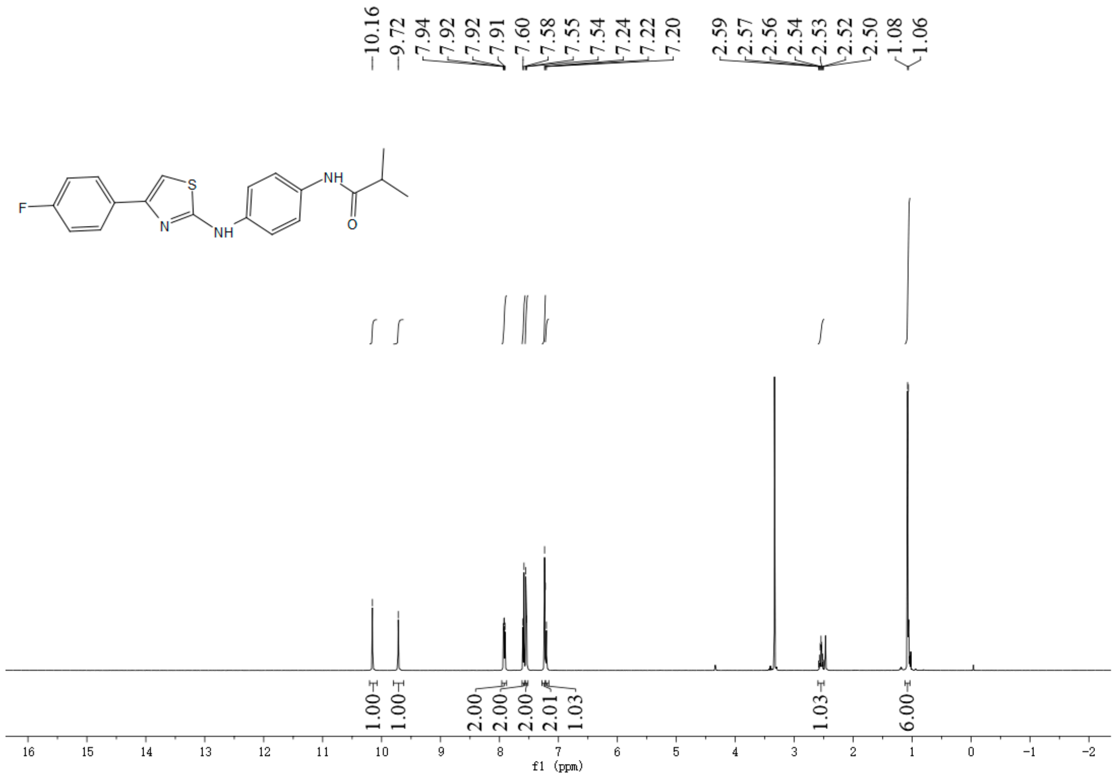

^{1}H NMR spectrum of compound **A**$_{25}$

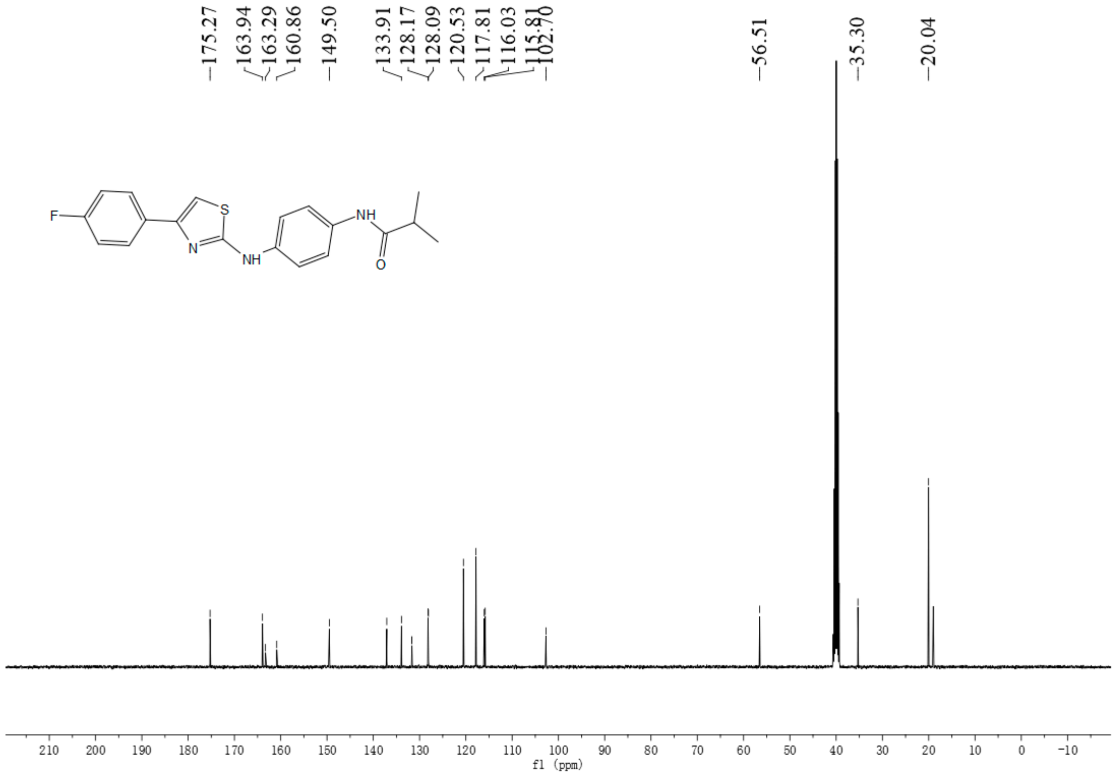

^{13}C NMR spectrum of compound **A**$_{25}$

（δ 56 (CH_2), δ 19(CH_3) in ^{13}C NMR spectrum of compound A$_{25}$ indicating a signal of impurity CH_3CH_2OH which should have been wiped out)

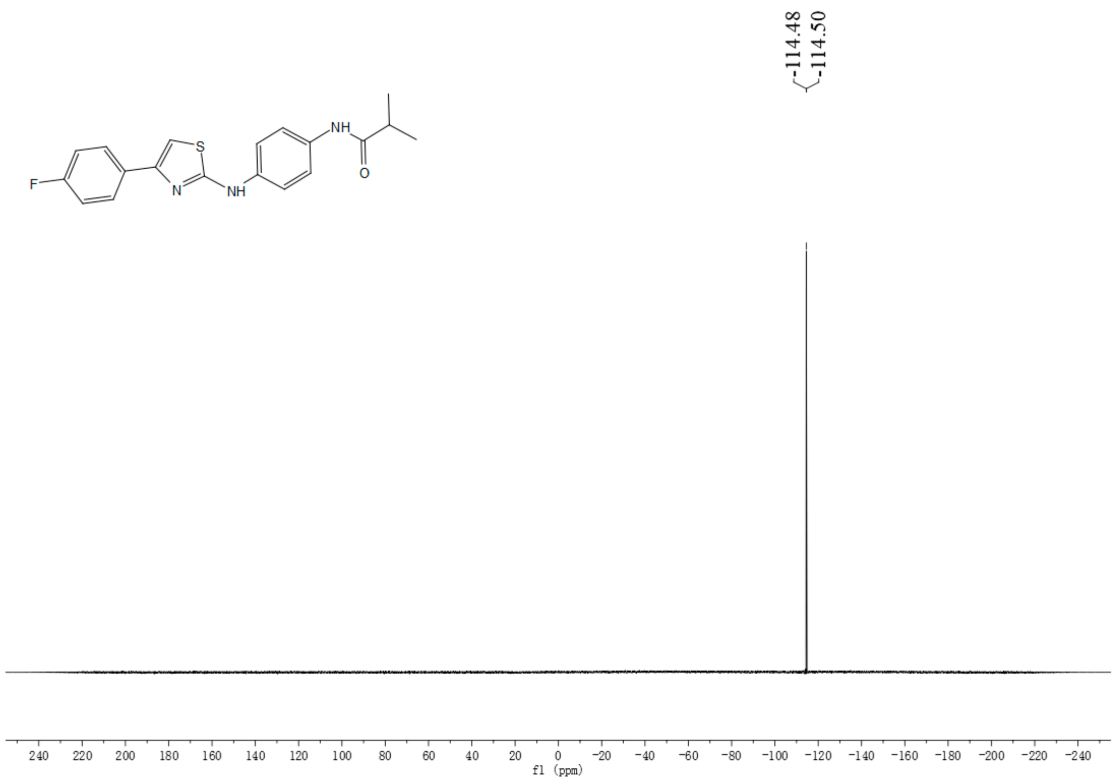

^{19}F NMR spectrum of compound **A**$_{25}$

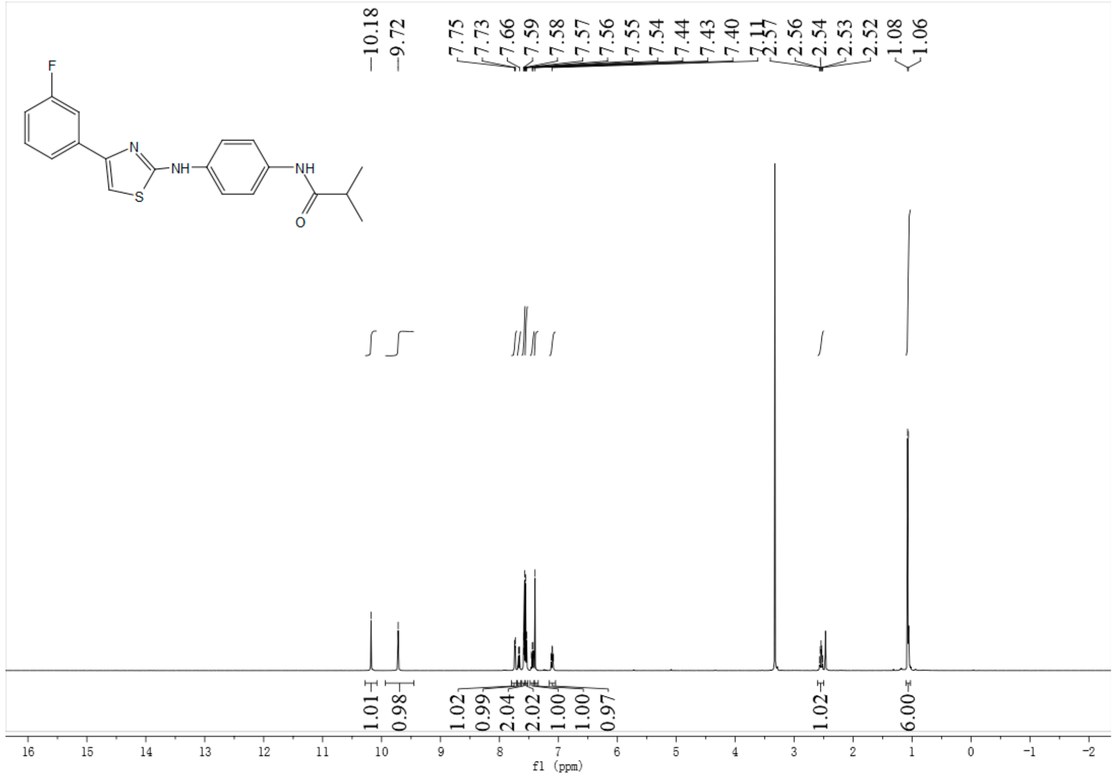

^{1}H NMR spectrum of compound **A**$_{26}$

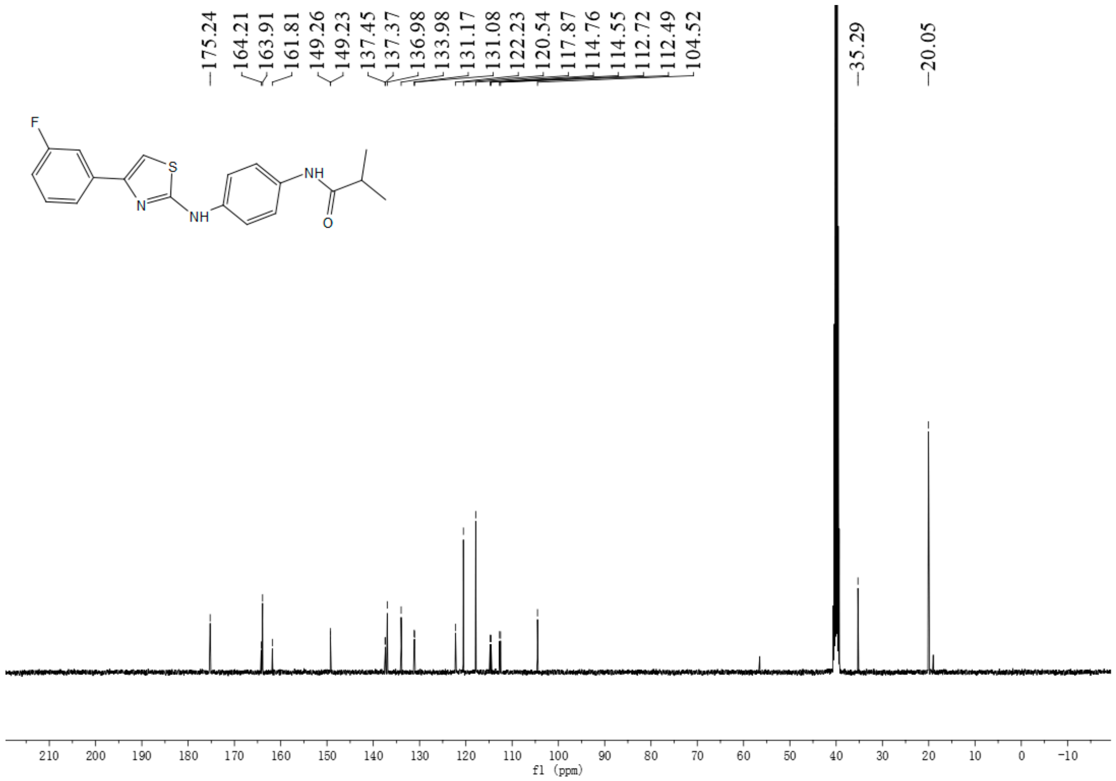

^{13}C NMR spectrum of compound **A**26

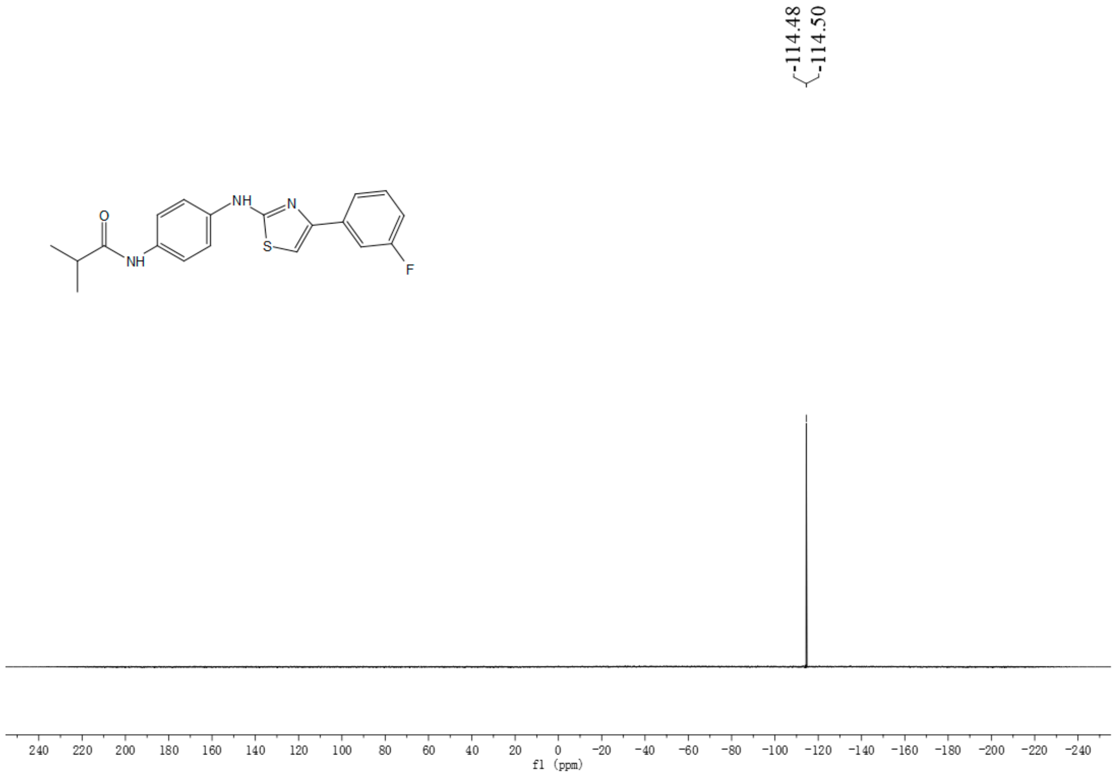

^{19}F NMR spectrum of compound **A**26

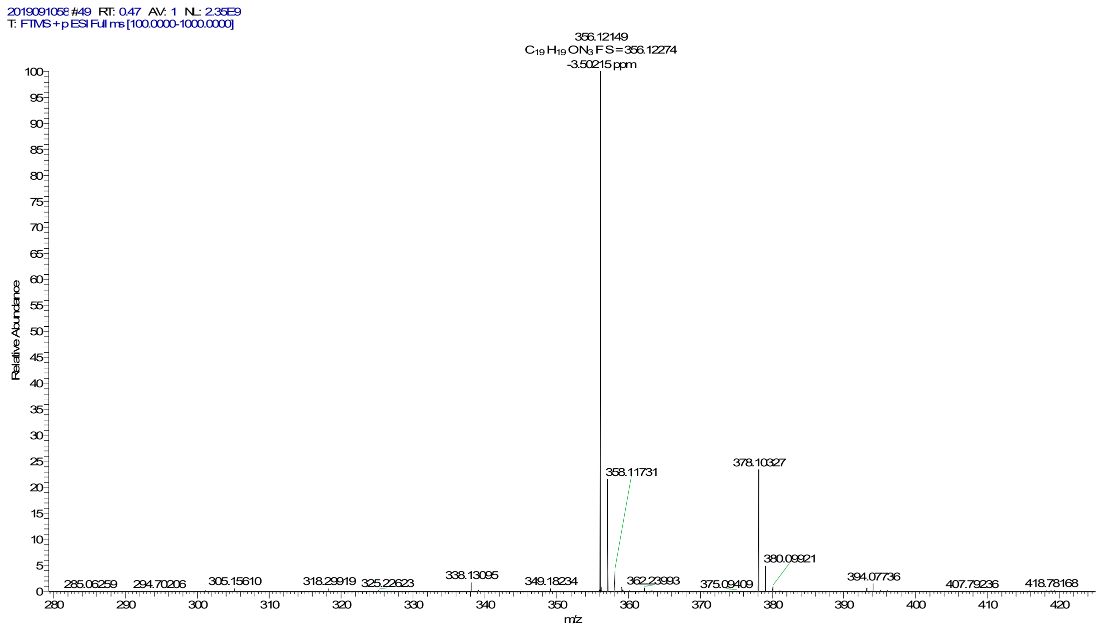

HRMS(ESI) of compound **A**26

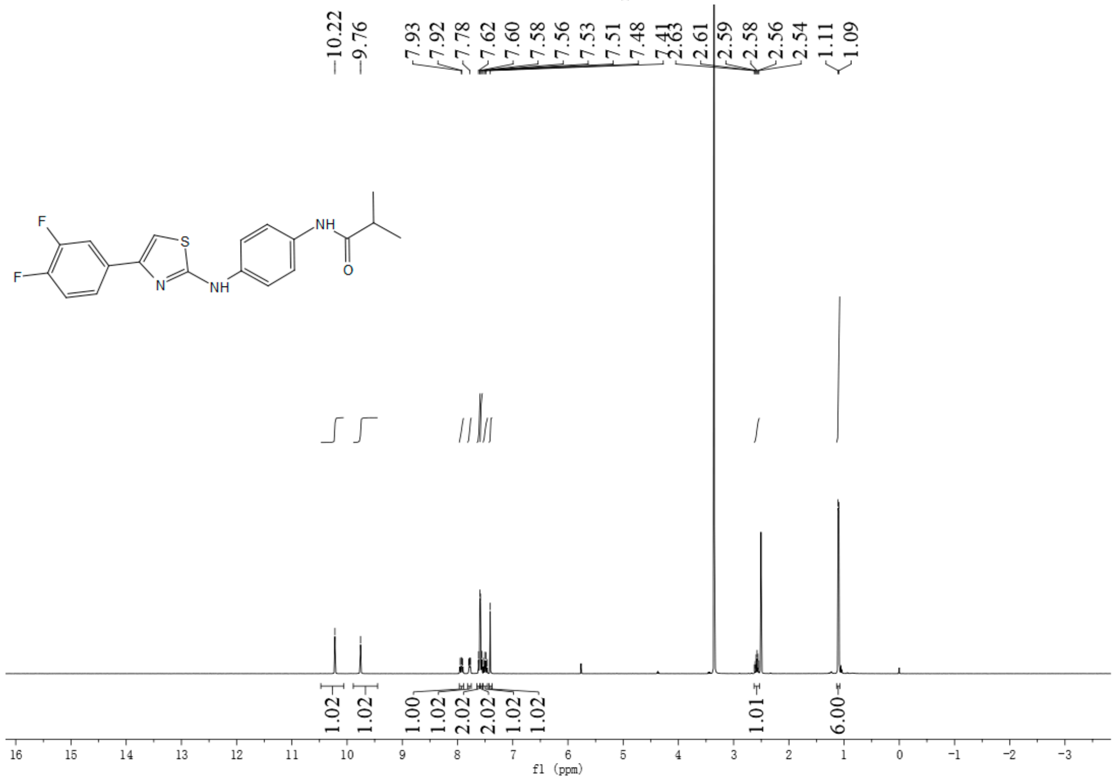

^{1}H NMR spectrum of compound **A**27

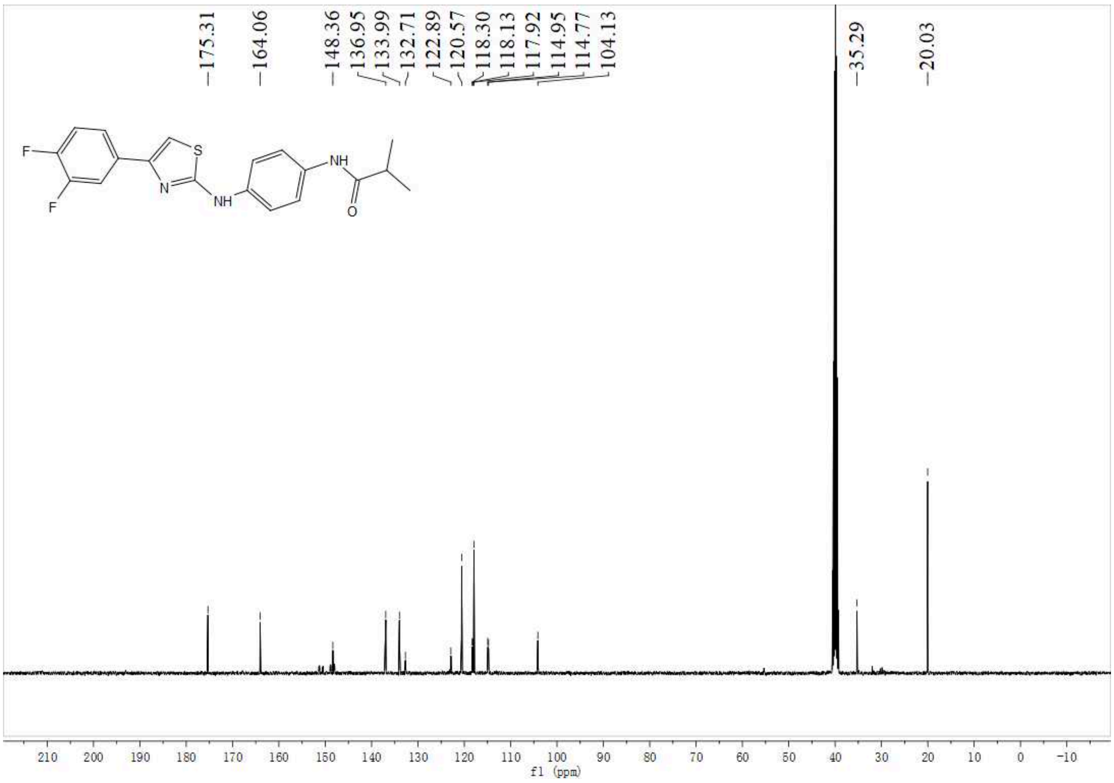

^{13}C NMR spectrum of compound **A**27

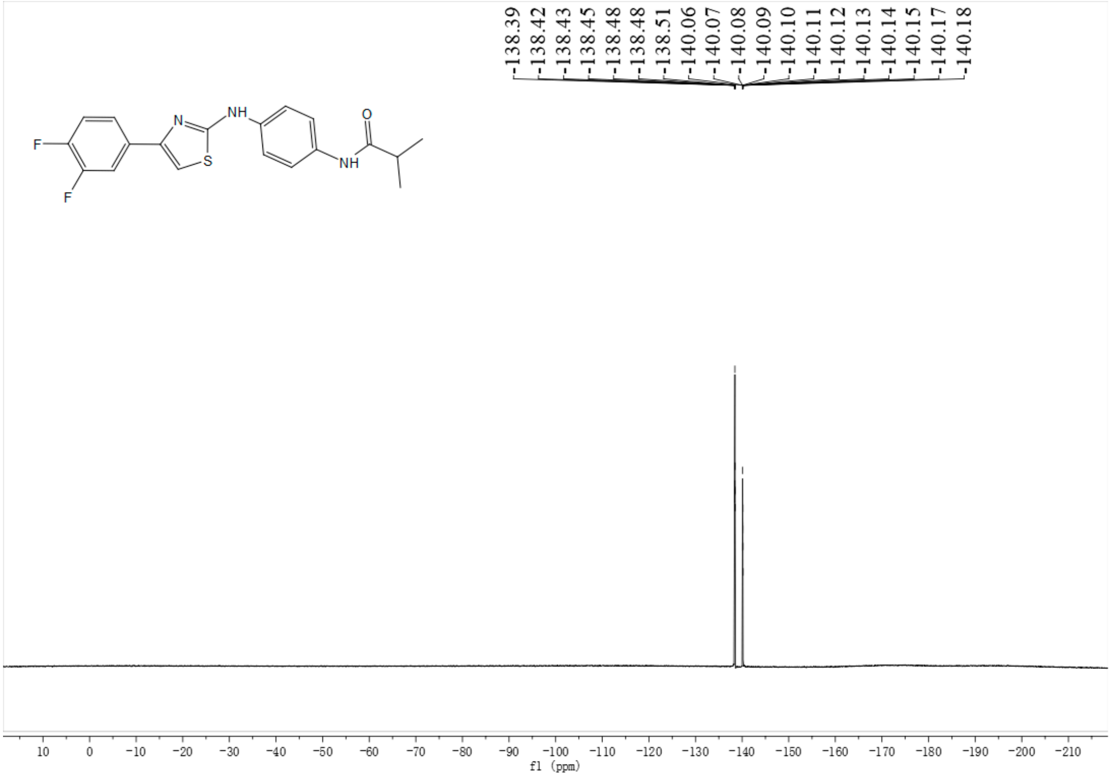

^{19}F NMR spectrum of compound **A**27

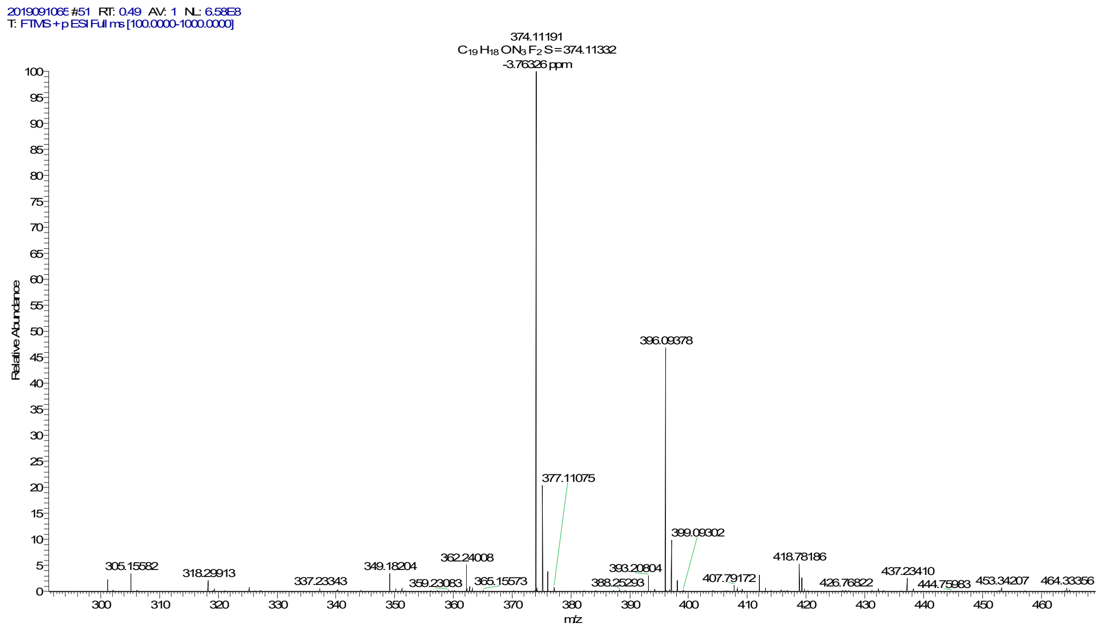

HRMS(ESI) of compound **A**27

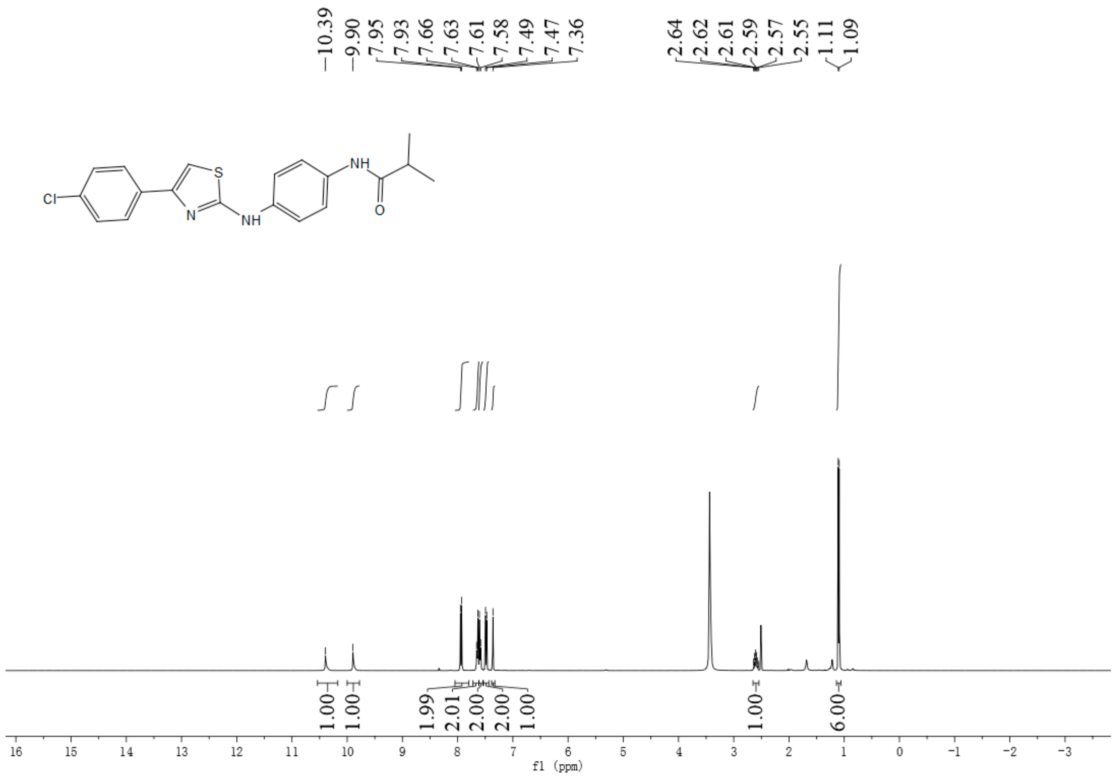

^{1}H NMR spectrum of compound **A**28

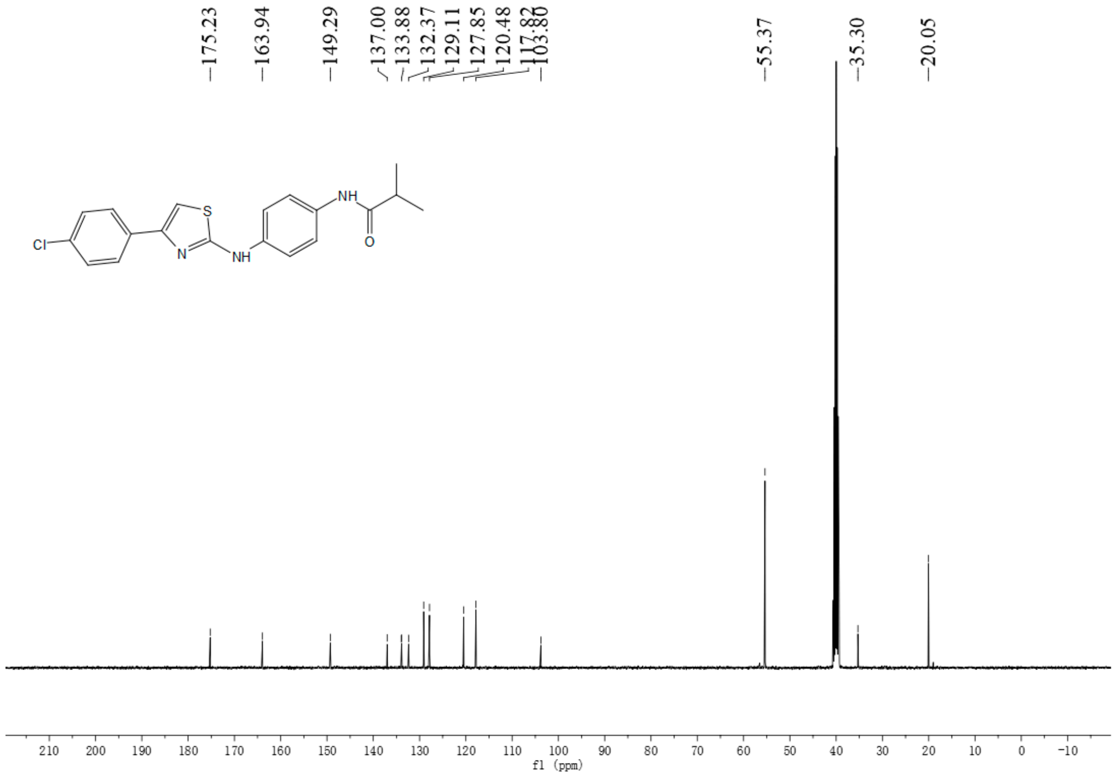

^{13}C NMR spectrum of compound **A**28

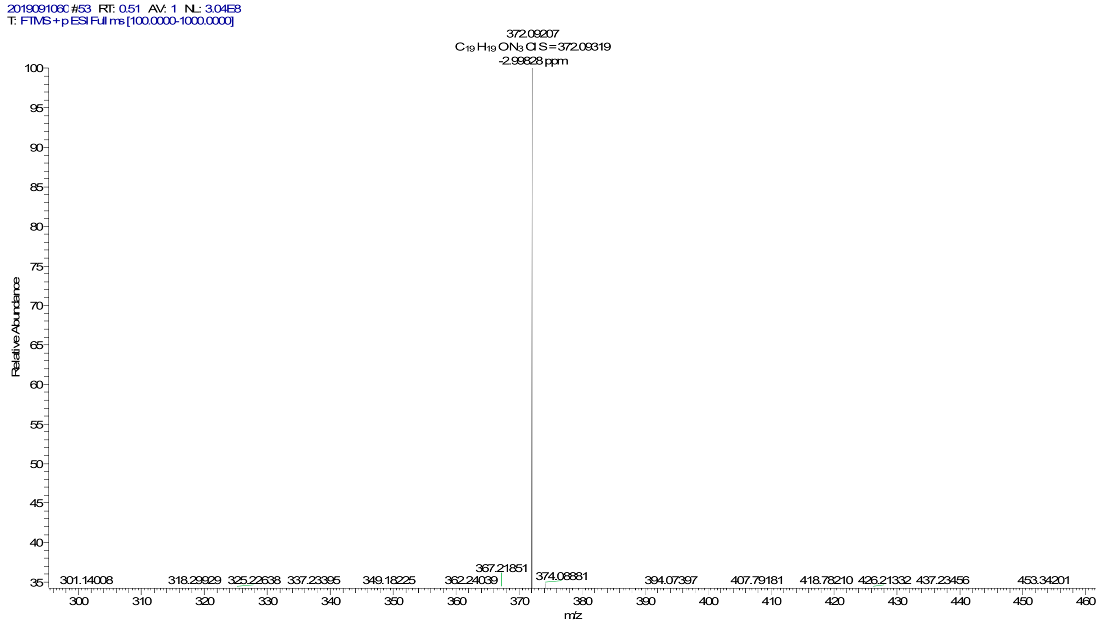

HRMS(ESI) of compound **A**28

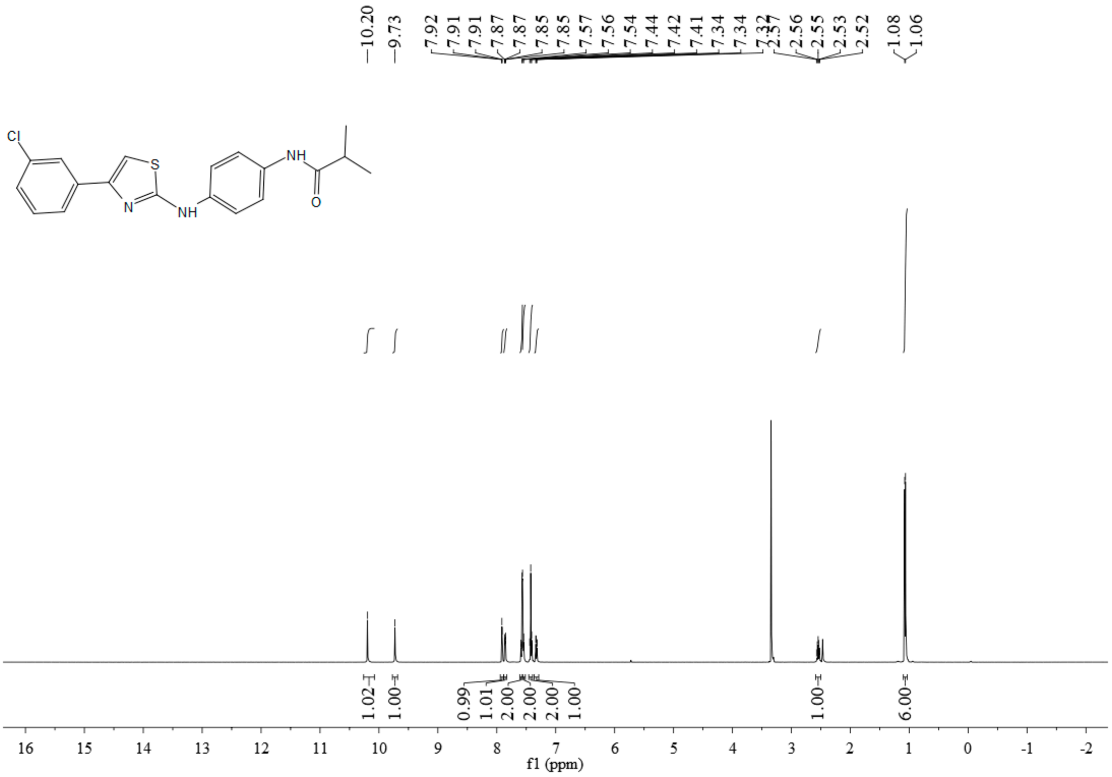

^{1}H NMR spectrum of compound **A**29

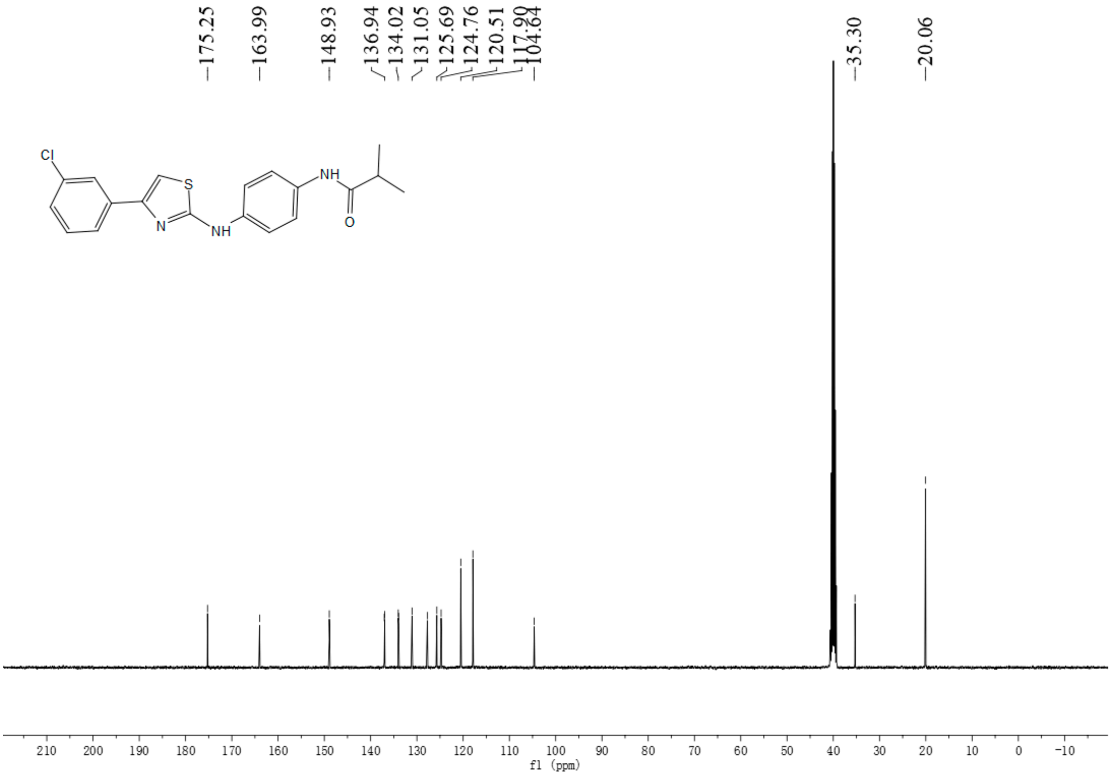

^{13}C NMR spectrum of compound **A**29

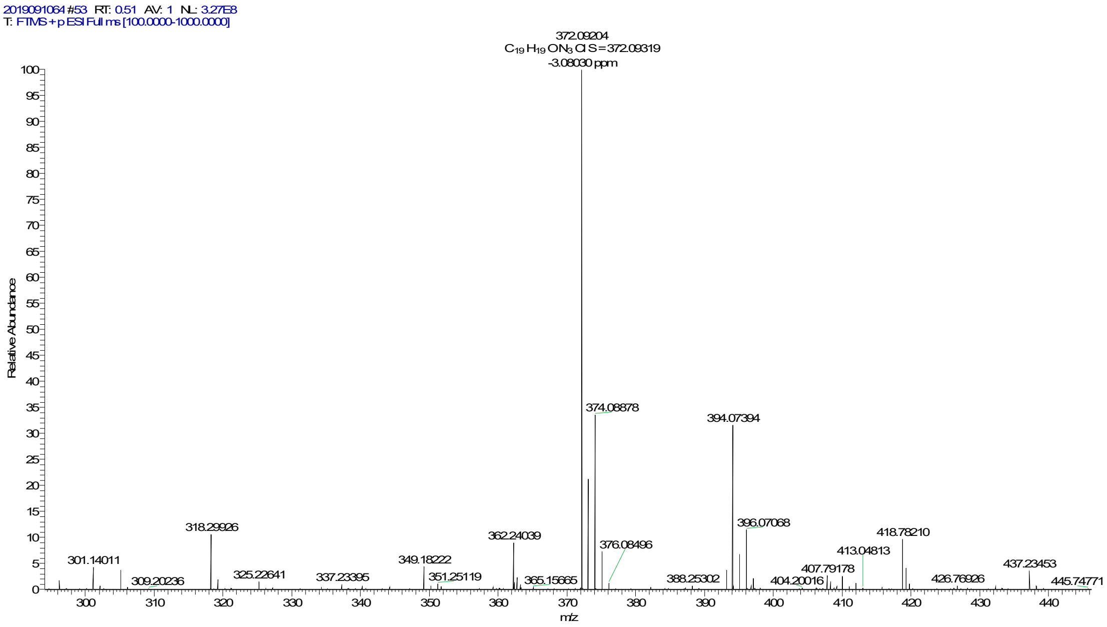

HRMS(ESI) of compound **A**29

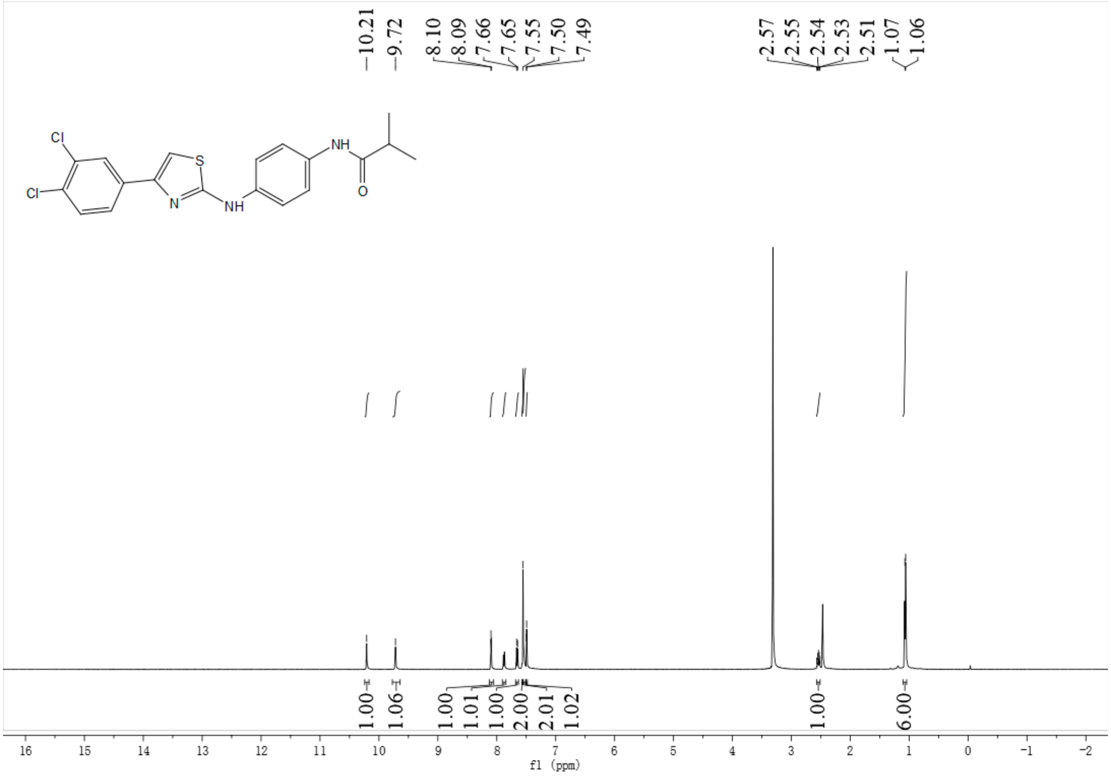

^{1}H NMR spectrum of compound **A**30

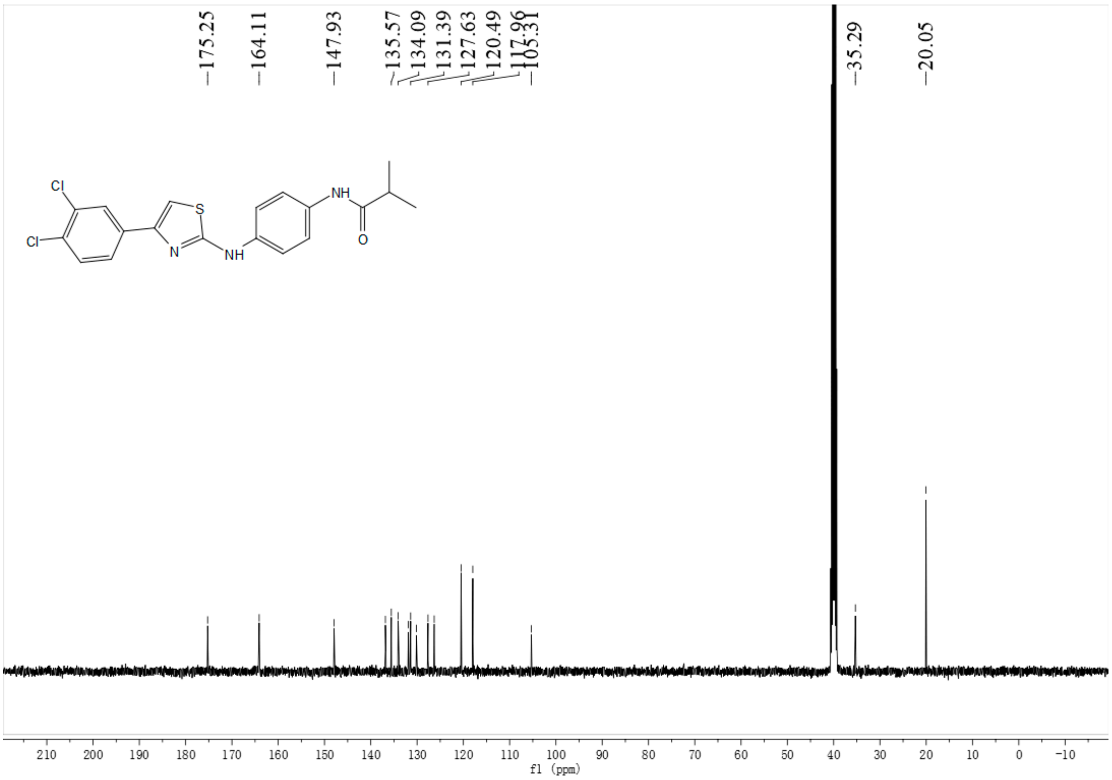

^{13}C NMR spectrum of compound **A**30

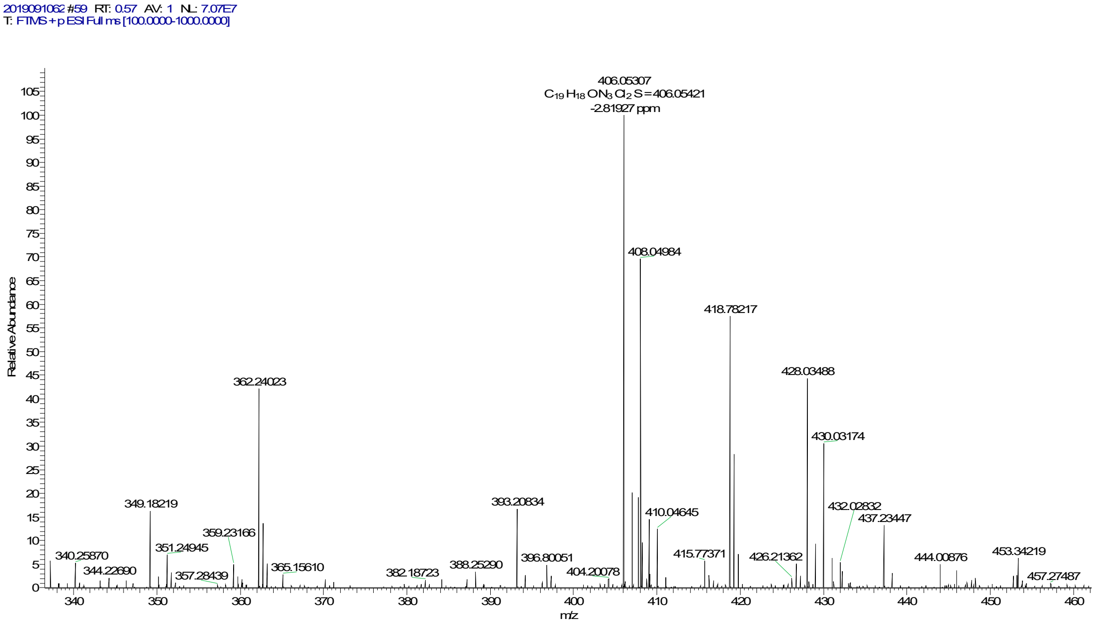

HRMS(ESI) of compound **A**30

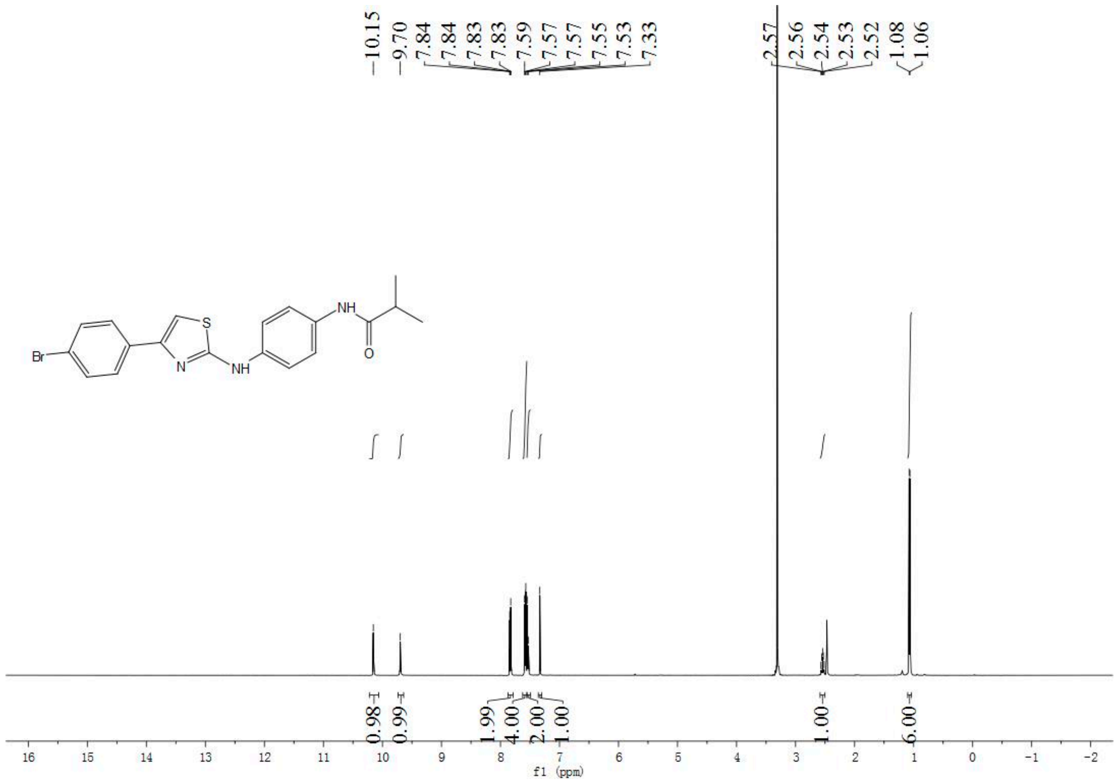

^{1}H NMR spectrum of compound **A**31

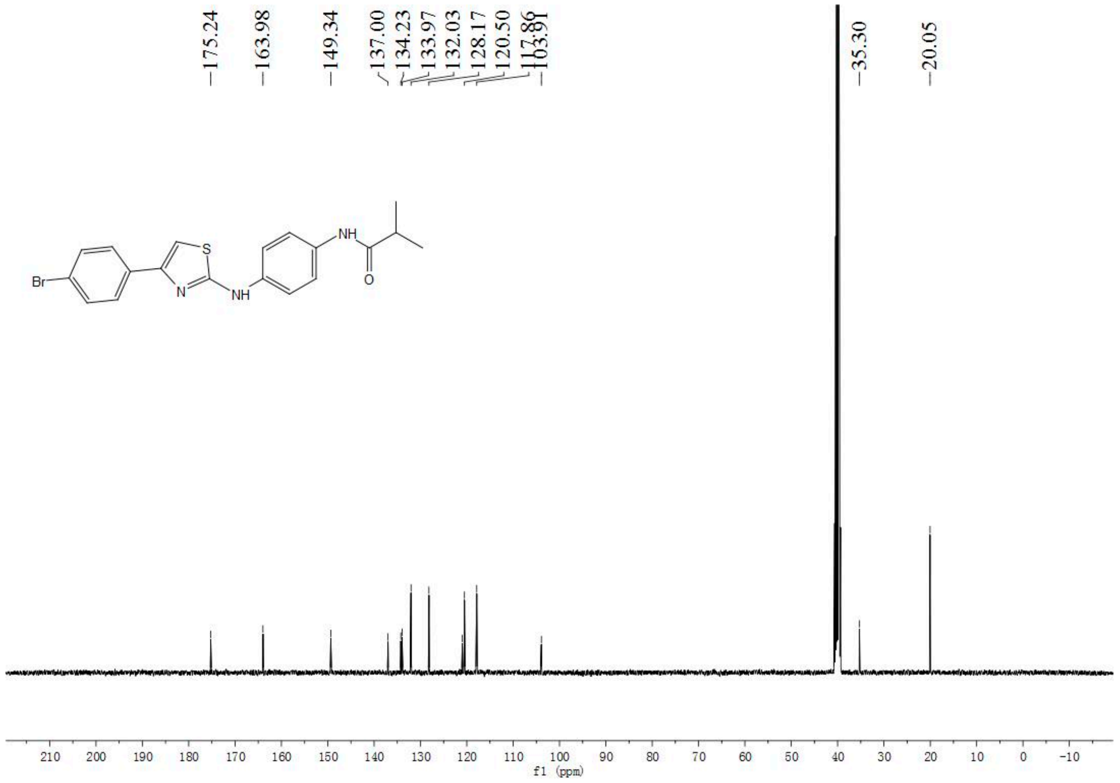

^{13}C NMR spectrum of compound **A**31

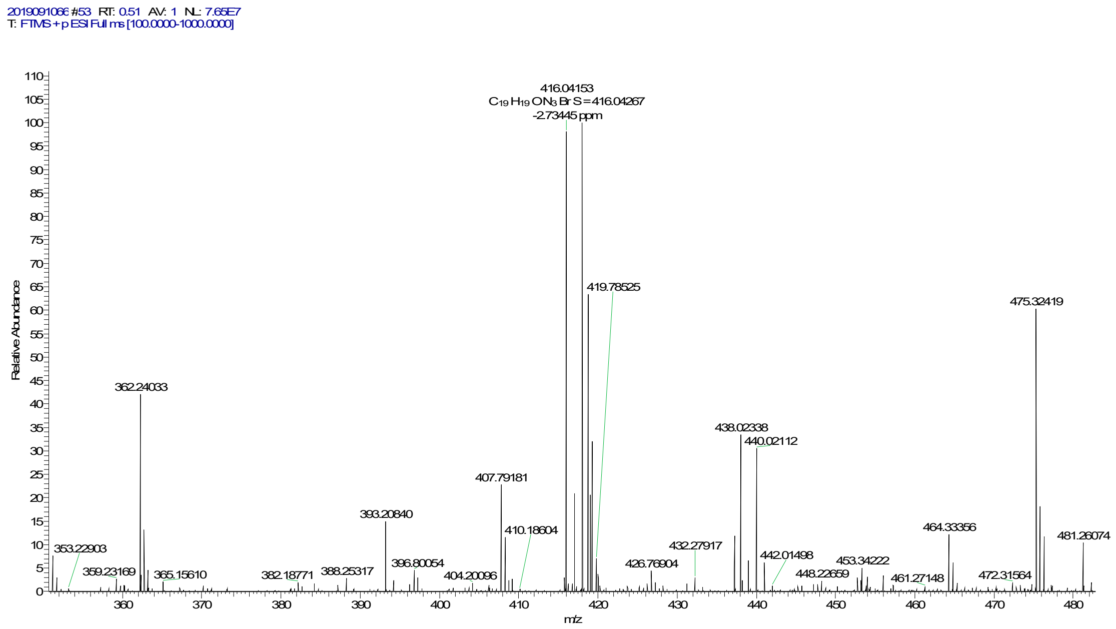

HRMS(ESI) of compound **A**31

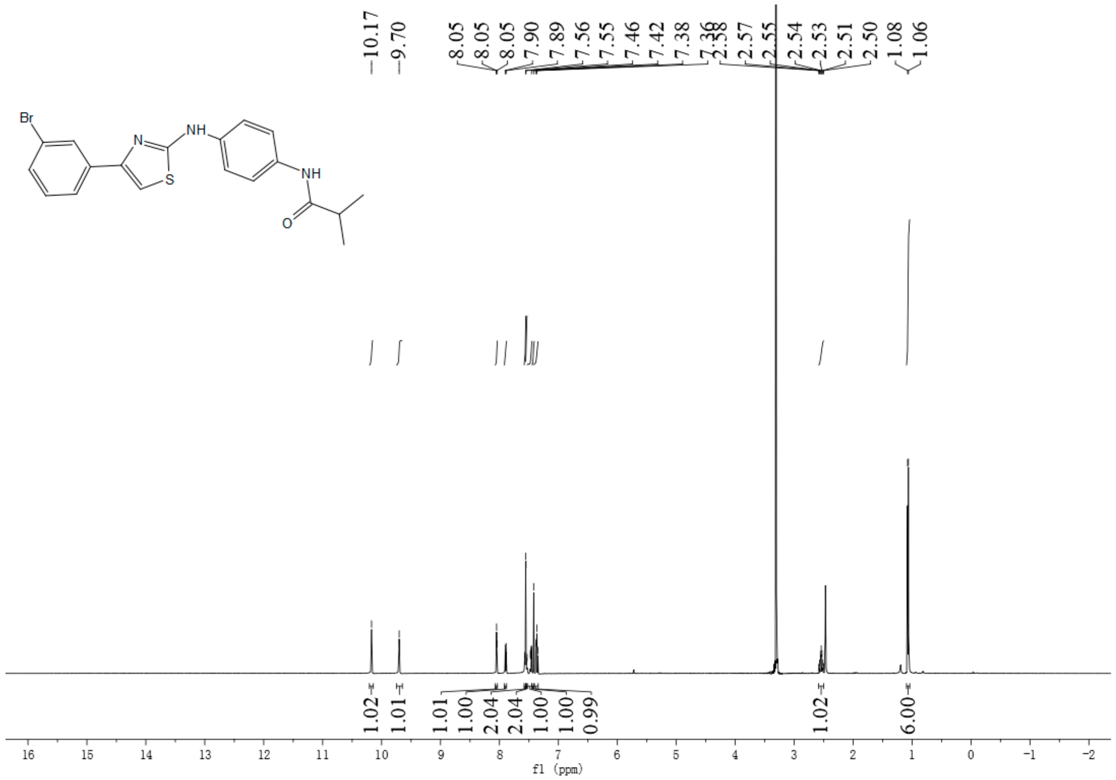

^{1}H NMR spectrum of compound **A**32

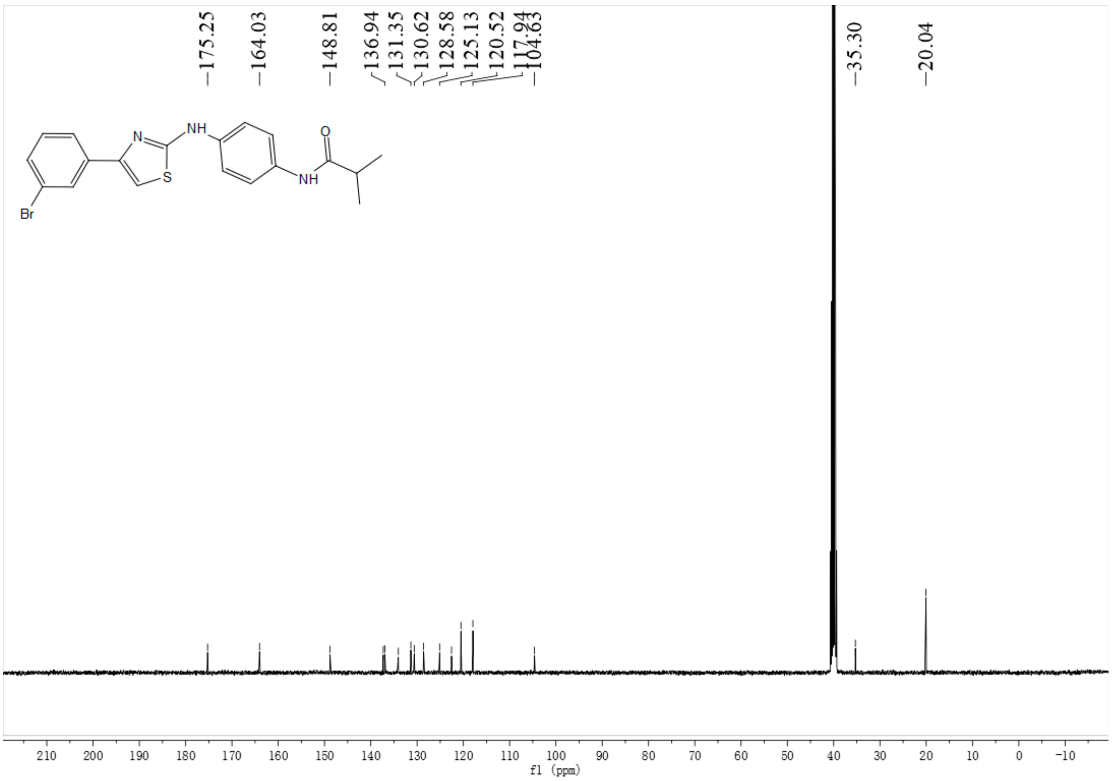

^{13}C NMR spectrum of compound **A**32

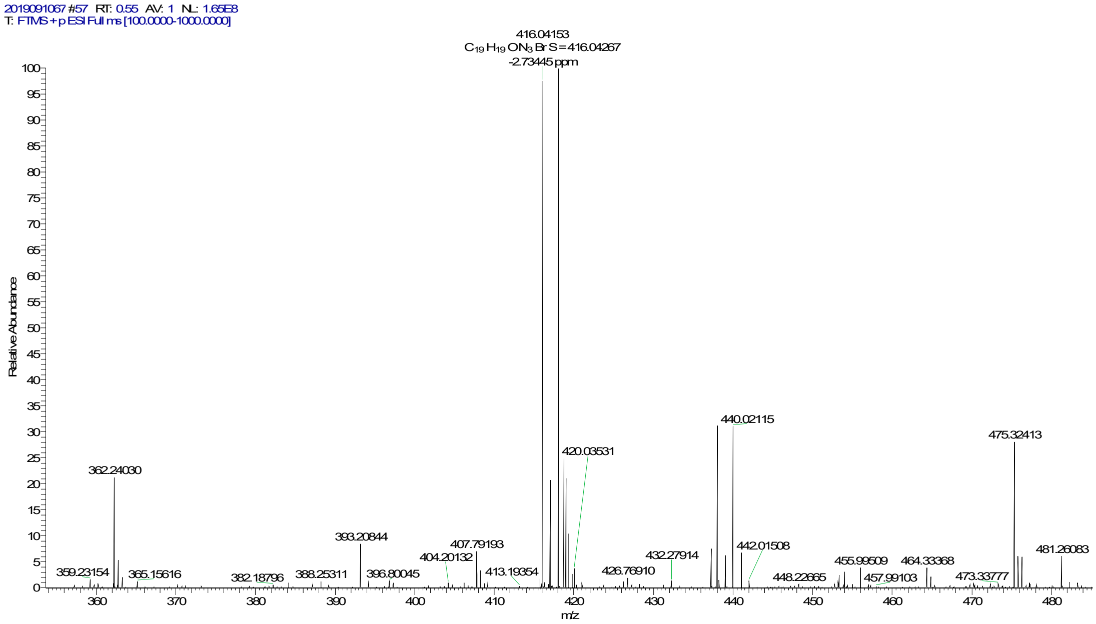

HRMS(ESI) of compound **A**32

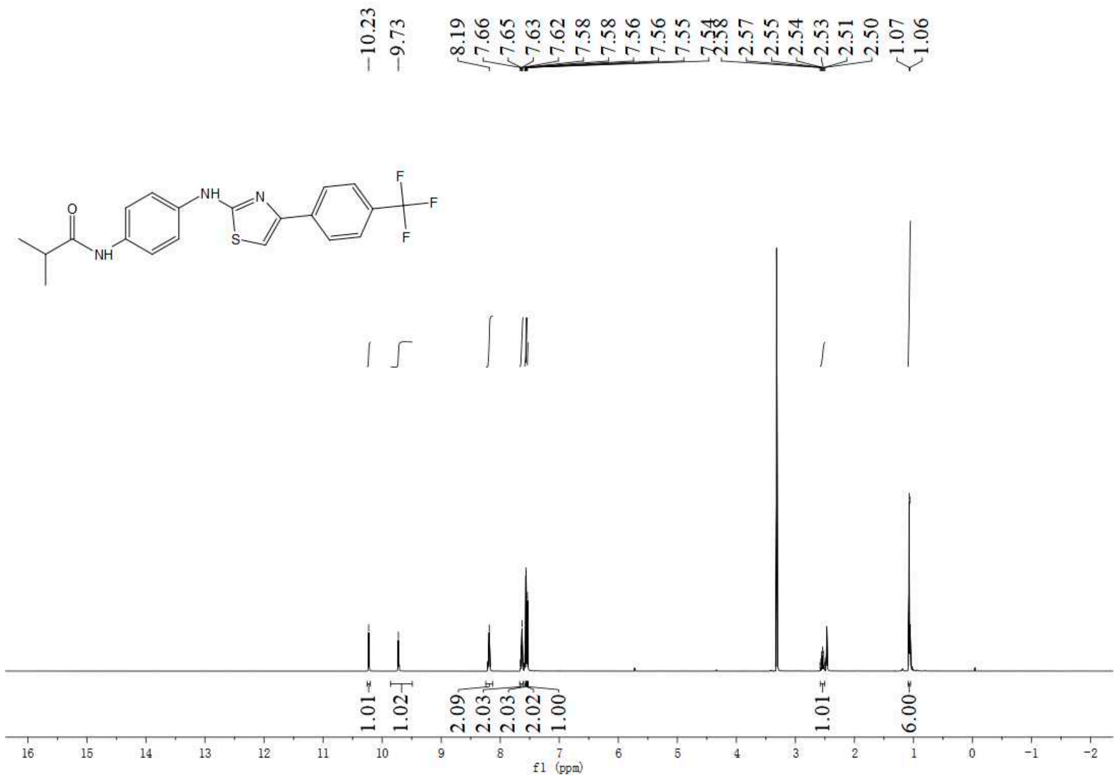

^{1}H NMR spectrum of compound **A**33

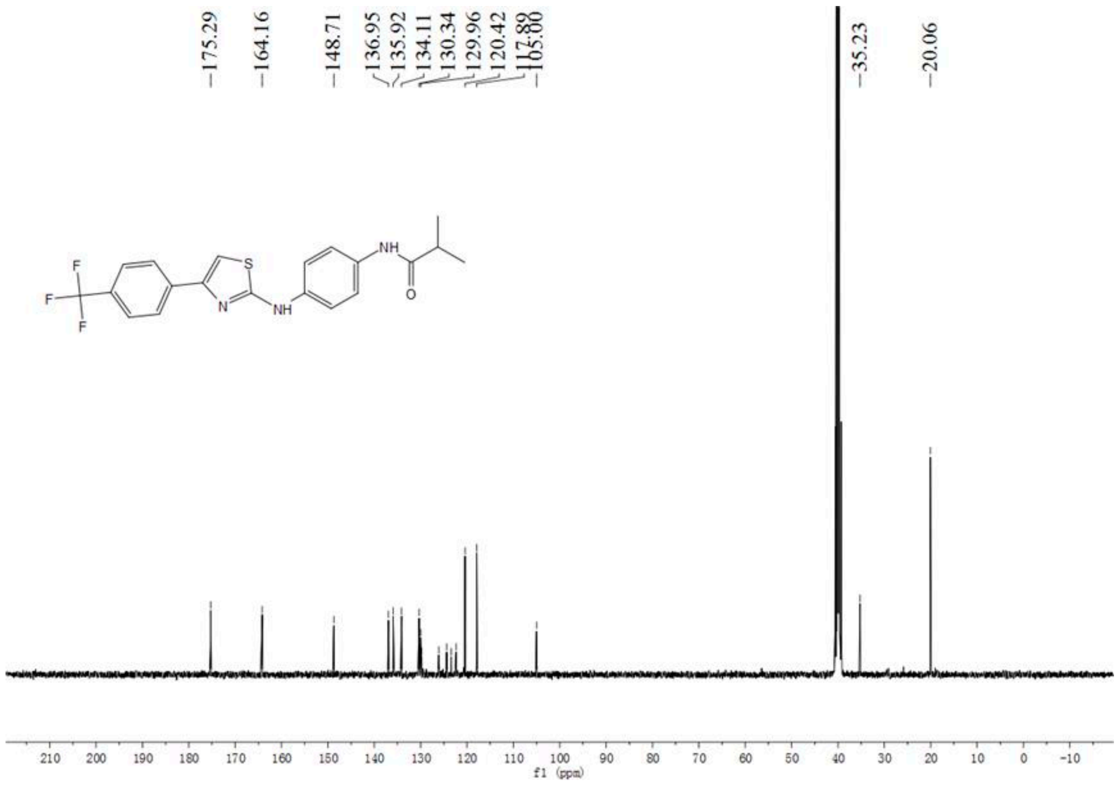

^{13}C NMR spectrum of compound **A**33

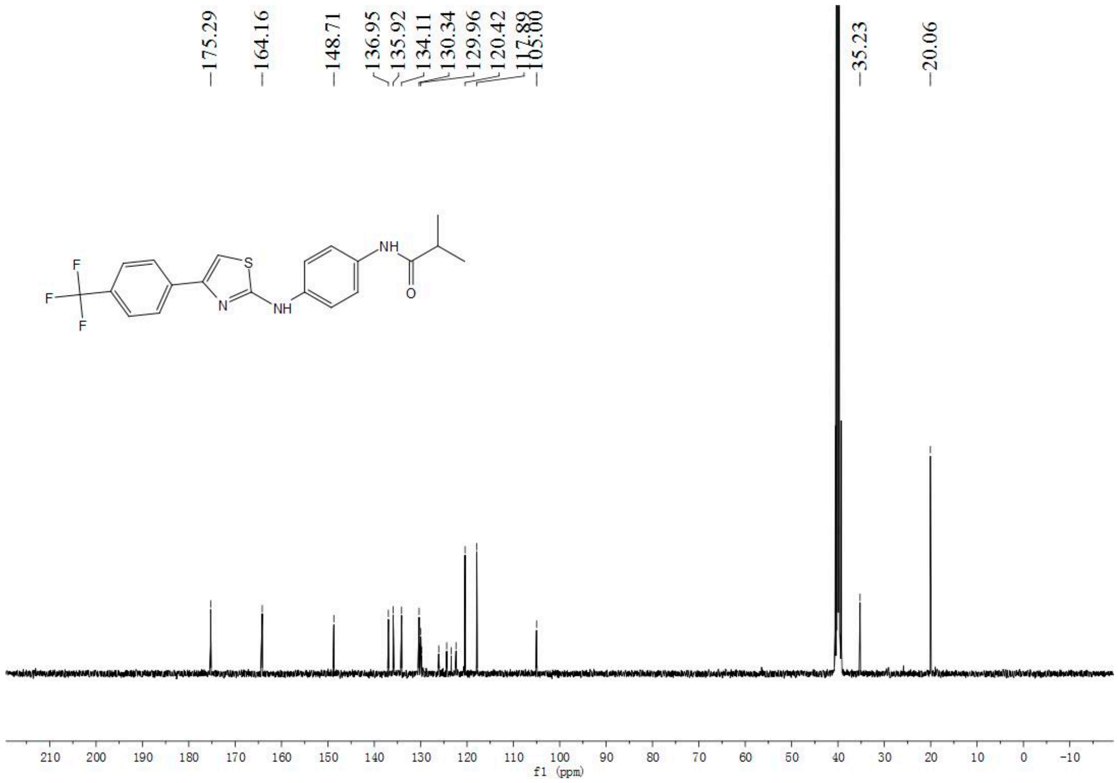

^{19}F NMR spectrum of compound **A**33

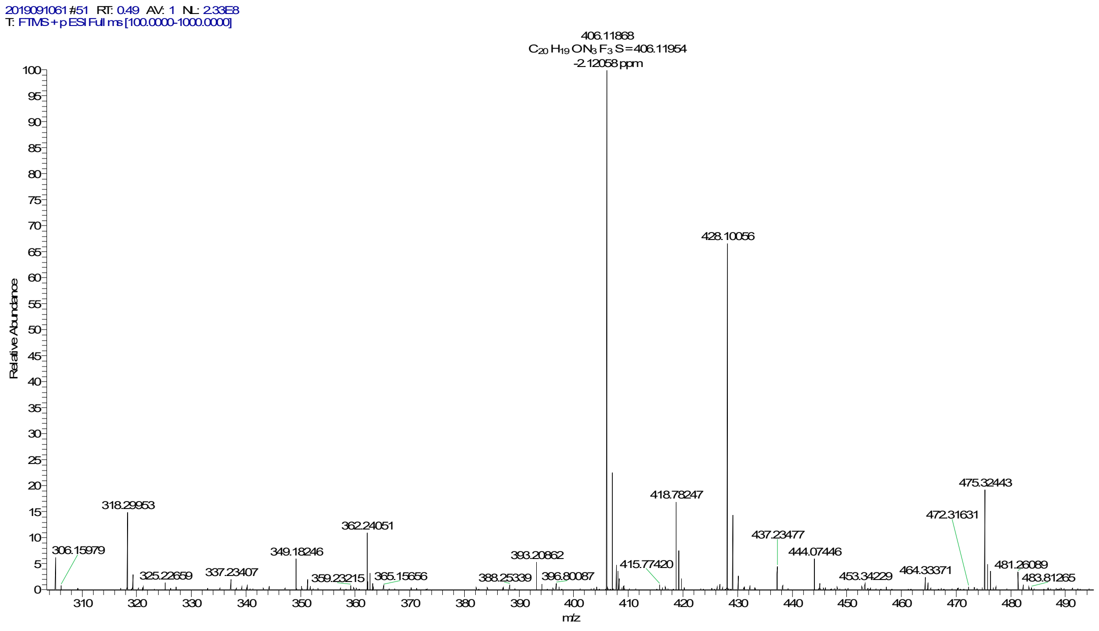

HRMS(ESI) of compound **A**33

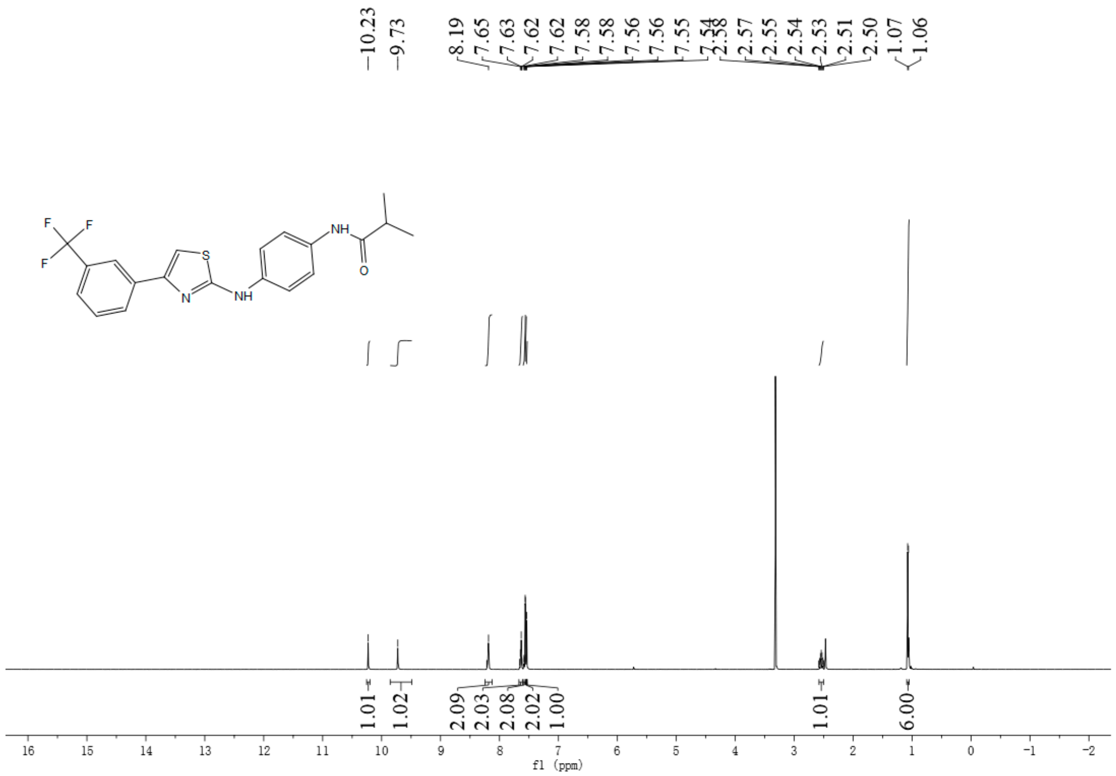

^{1}H NMR spectrum of compound **A**34

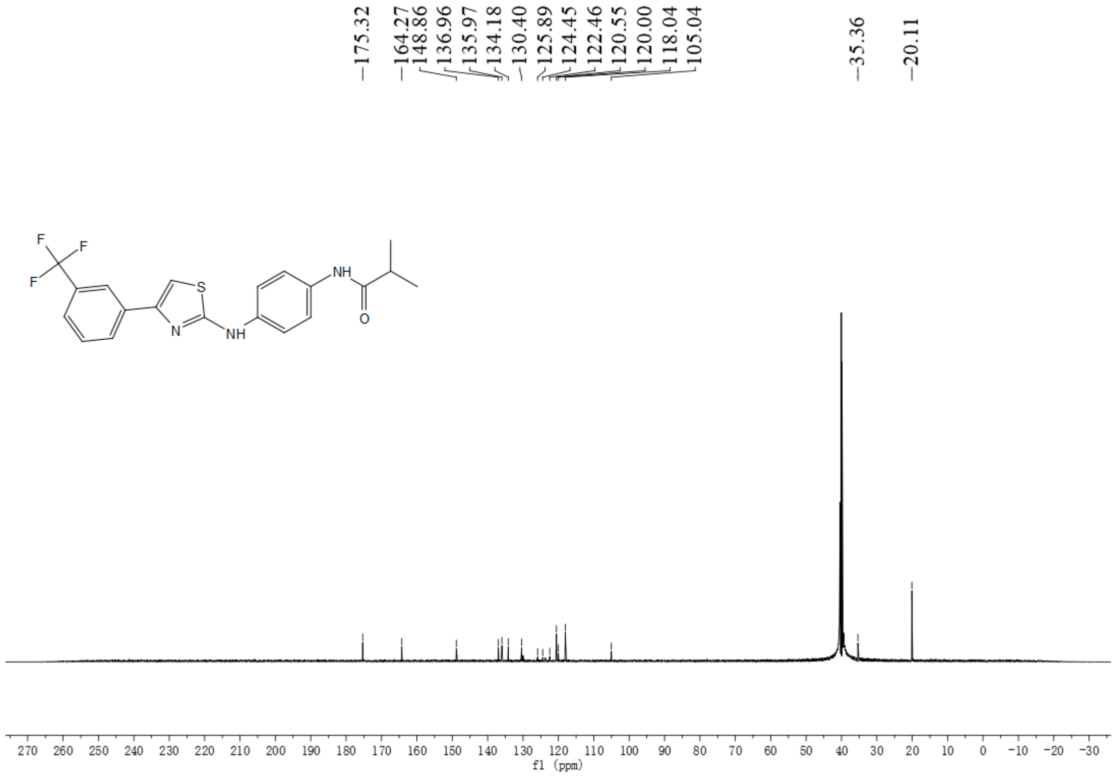

^{13}C NMR spectrum of compound **A**34

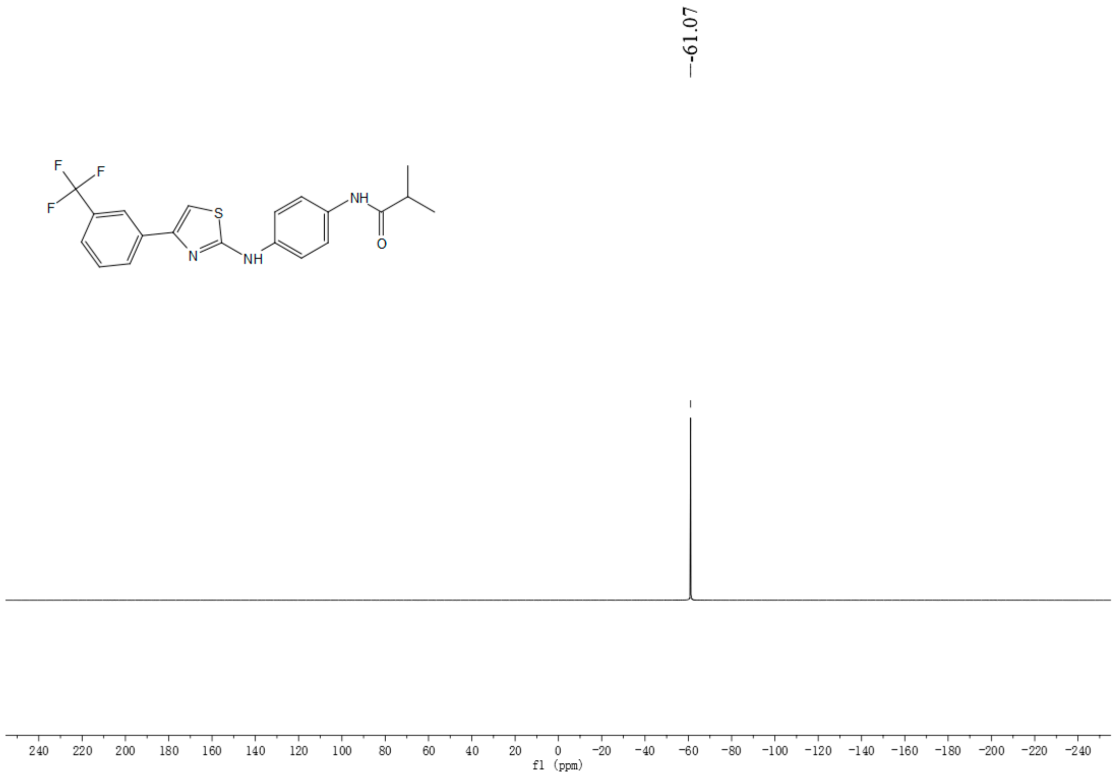

^{19}F NMR spectrum of compound **A**34

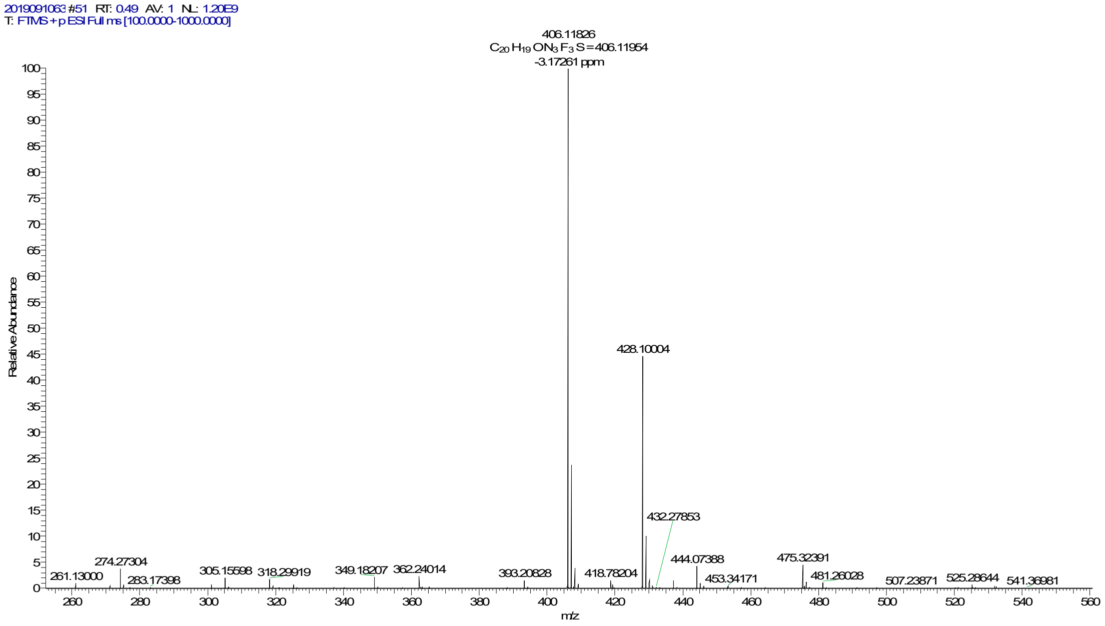

HRMS(ESI) of compound **A**34

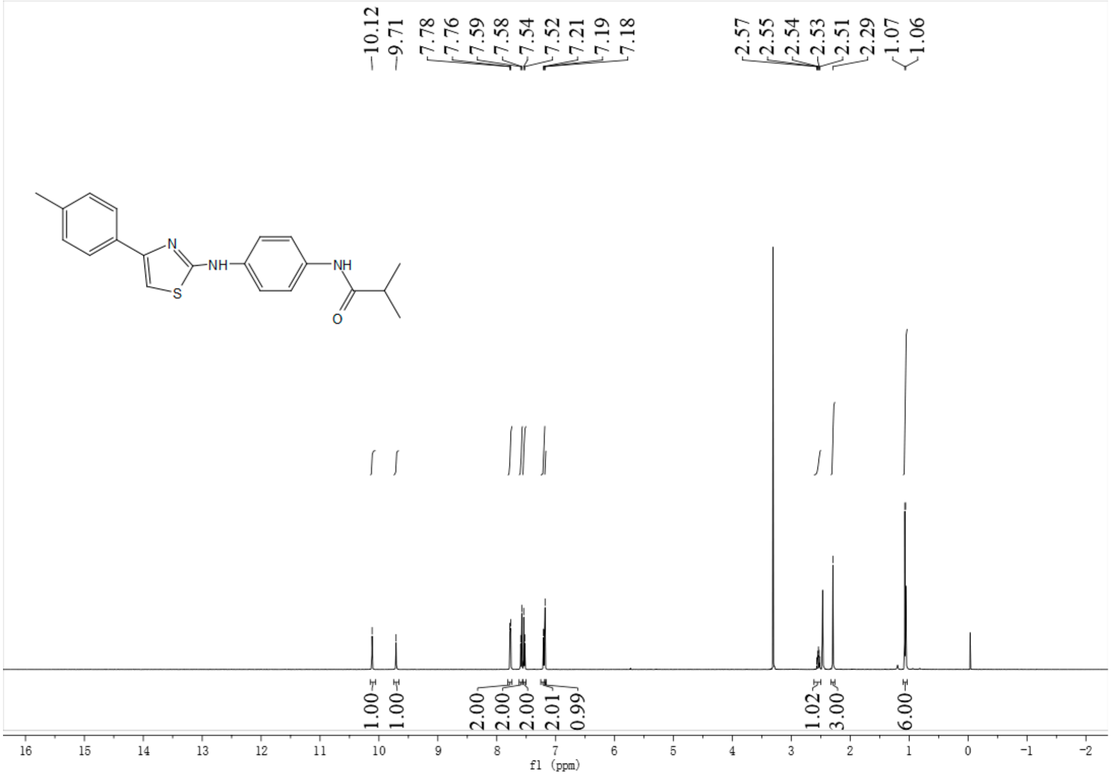

^{1}H NMR spectrum of compound **A**35

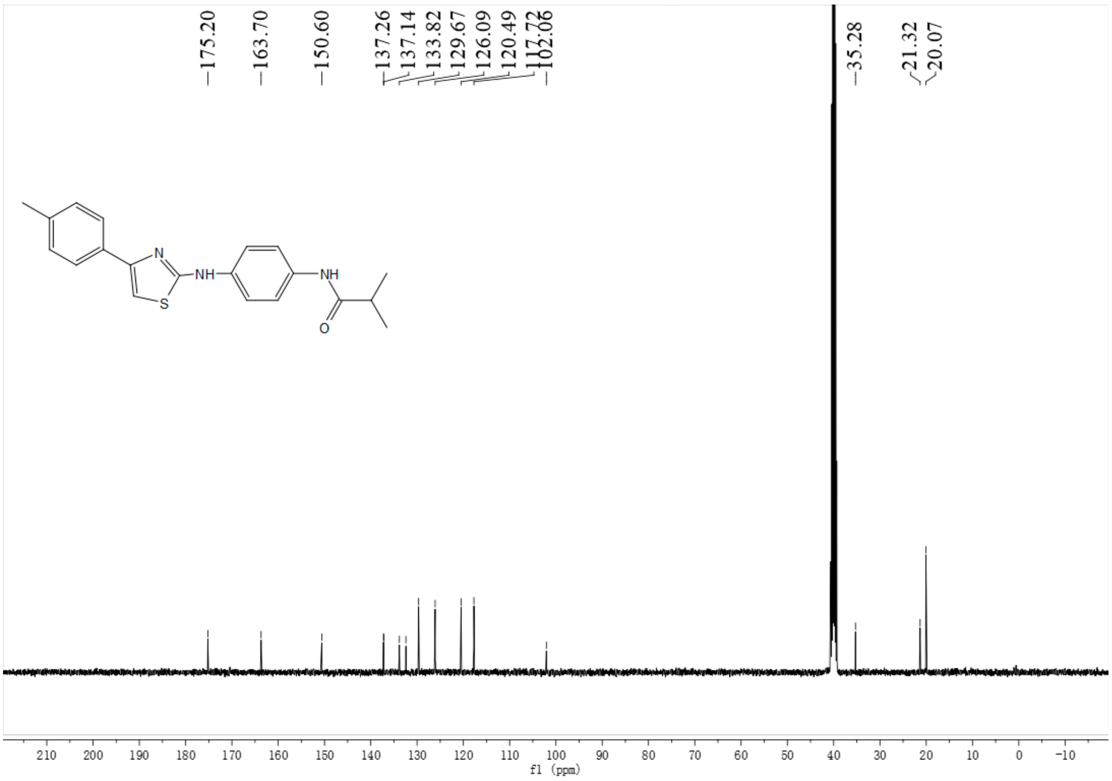

^{13}C NMR spectrum of compound **A**35

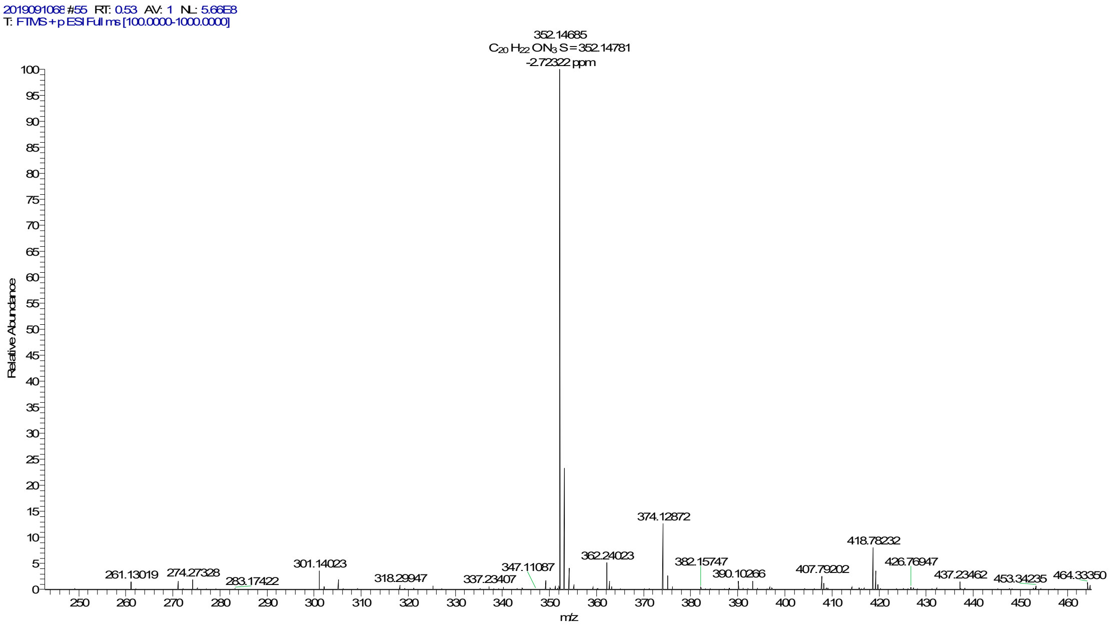

HRMS(ESI) of compound **A**35

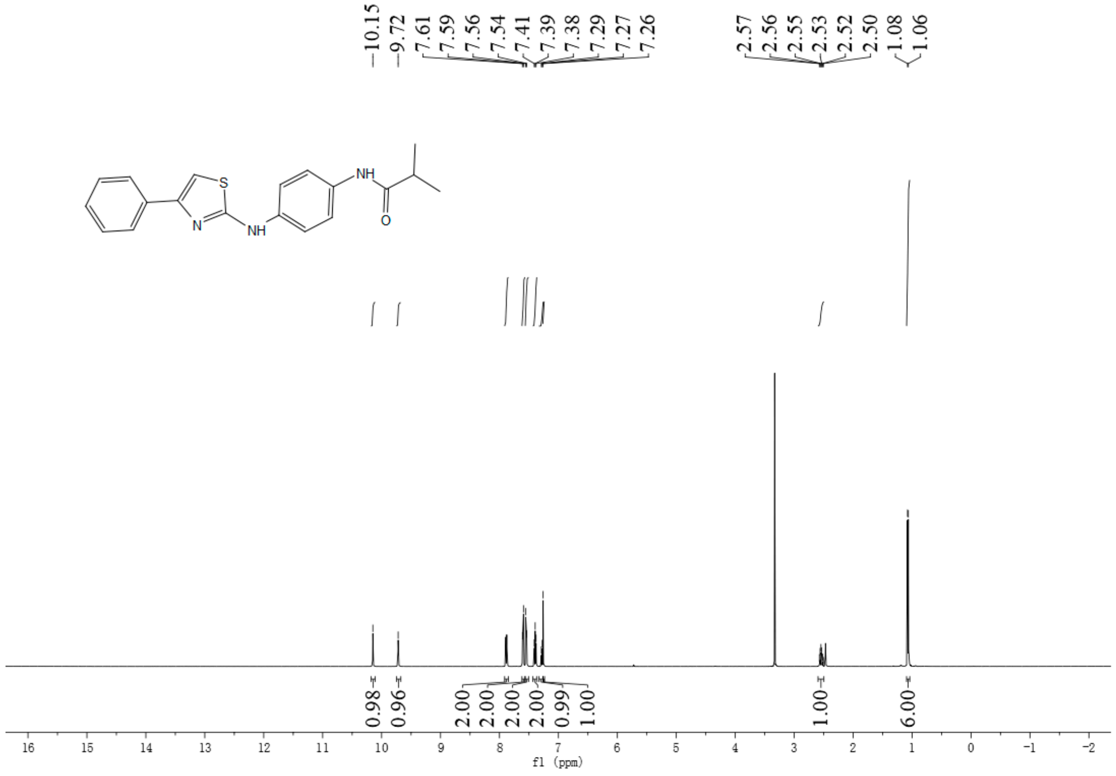

^{1}H NMR spectrum of compound **A**36

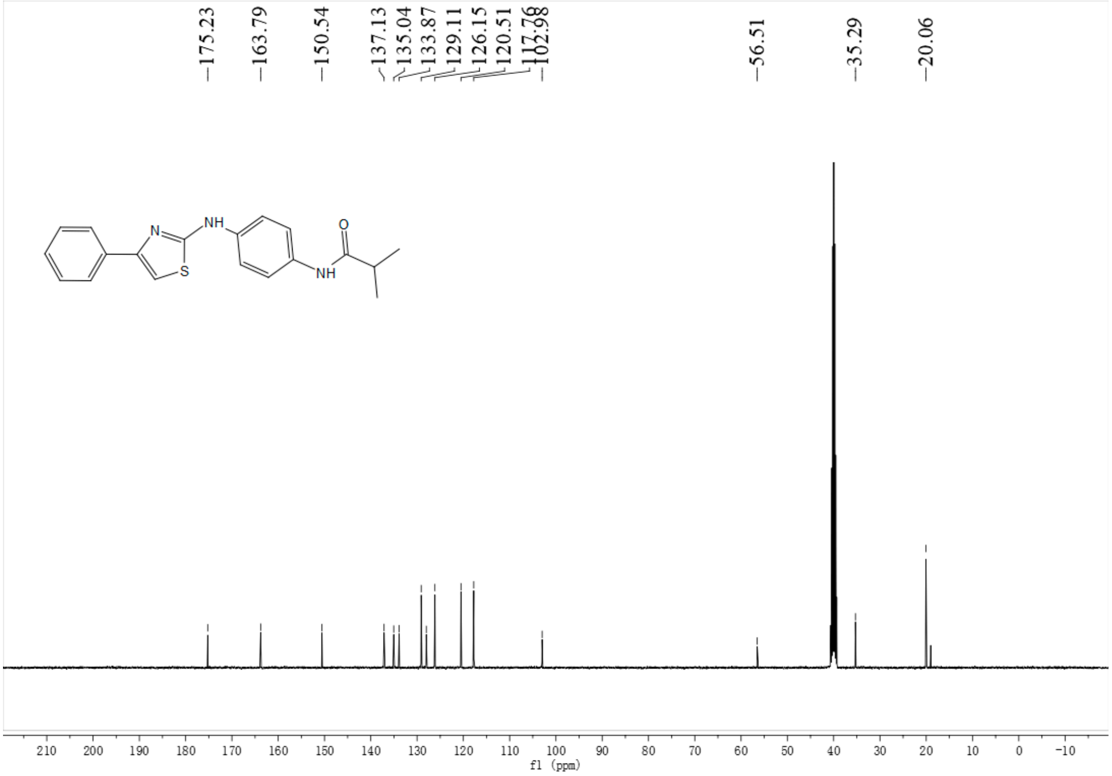

^{13}C NMR spectrum of compound **A**$_{36}$

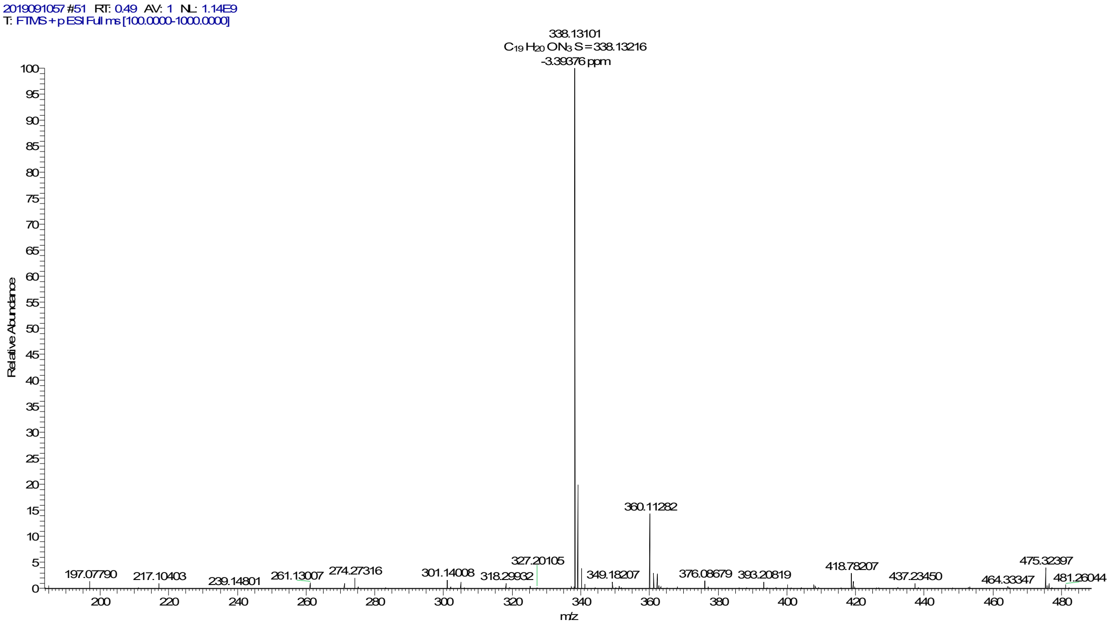

HRMS(ESI) of compound **A**$_{36}$

Table S1. Inhibition rate (%) of compounds $\mathbf{A}_1$-$\mathbf{A}_{36}$ against *Xoo*, *Xac* and *Xoc*.

Compd.	R′/R	*Xoo*		*Xac*		*Xoc*	
		200 μg/mL	100 μg/mL	200 μg/L	100 μg/mL	200 μg/mL	100 μg/mL
$\mathbf{A}_1$	4-F/CH_3	98±1.67	82±0.64	78±3.65	51±1.83	87±2.79	55±3.75
$\mathbf{A}_2$	3-F/CH_3	25±1.09	20±0.82	53±1.55	26±3.96	45±6.80	35±1.68
$\mathbf{A}_3$	3,4-diF /CH_3	76±3.40	59±1.72	44±5.50	21±1.80	73±3.64	43±1.97
$\mathbf{A}_4$	4-Cl/CH_3	85±2.56	68±0.78	75±4.70	45±7.59	81±0.94	62±2.66
$\mathbf{A}_5$	3-Cl/CH_3	47±1.42	13±1.56	36±0.11	16±0.13	56±1.25	35±1.75
$\mathbf{A}_6$	3,4-diCl/CH_3	87±2.1	67±3.20	50±3.10	40±1.41	69±6.11	47±1.48
$\mathbf{A}_7$	4-Br/CH_3	52±0.61	45±1.70	32±1.81	21±0.60	61±1.73	48±2.01
$\mathbf{A}_8$	3-Br/CH_3	38±0.159	23±0.19	31±1.14	29±1.25	57±2.93	30±3.31
$\mathbf{A}_9$	4-CF_3/CH_3	52±0.50	29±0.91	62±1.76	35±4.31	46±1.74	22±1.27
$\mathbf{A}_{10}$	3-CF_3/CH_3	41±1.50	22.4±2.30	18±0.91	16±1.15	35±2.93	19±3.87
$\mathbf{A}_{11}$	4-CH_3/CH_3	61±5.20	52±3.30	39±1.10	32±1.31	37±1.94	7±2.30
$\mathbf{A}_{12}$	H/CH_3	54±1.40	19±2.84	40±3.89	38±1.62	49±1.52	28±3.41
$\mathbf{A}_{13}$	4-F/CH_2CH_3	35±1.90	24±3.72	47±1.90	36±6.37	61±1.67	40±1.91
$\mathbf{A}_{14}$	3-F/CH_2CH_3	41±1.81	17±5.95	38±2.81	27±0.95	55±2.16	21±2.24
$\mathbf{A}_{15}$	3,4-diF/CH_2CH_3	61±1.10	37±4.80	49±2.20	31±4.10	52±5.06	38±4.73
$\mathbf{A}_{16}$	4-Cl/CH_2CH_3	45±1.93	35±2.79	39±2.23	26±1.76	63±1.41	43±1.09
$\mathbf{A}_{17}$	3-Cl/CH_2CH_3	24±2.60	11±5.80	17±2.36	14±2.60	50±3.07	37±2.00
$\mathbf{A}_{18}$	3,4-diCl/CH_2CH_3	35±3.10	13±1.10	52±3.50	33±1.30	47±1.41	10±1.09
$\mathbf{A}_{19}$	4-Br/CH_2CH_3	39±0.70	34±1.81	67±5.65	60±2.59	19±5.60	4±1.82
$\mathbf{A}_{20}$	3-Br/CH_2CH_3	31±4.70	29±3.40	25±4.05	18±4.74	45±3.74	17±2.50
$\mathbf{A}_{21}$	4-CF_3/CH_2CH_3	54±0.60	23±1.20	52±1.47	39±3.14	45±0.78	7±2.17
$\mathbf{A}_{22}$	3-CF_3/CH_2CH_3	26±0.24	19±0.28	31±4.18	25±3.31	39±2.40	10±0.78
$\mathbf{A}_{23}$	4-CH_3 /CH_2CH_3	33±0.73	22±0.90	49±4.98	40±3.96	34±4.48	16±1.12
$\mathbf{A}_{24}$	H/CH_2CH_3	40±2.94	29±5.78	36±5.80	25±3.38	55±5.98	48±1.18
$\mathbf{A}_{25}$	4-F/$CH(CH_3)_2$	40±1.35	37±0.92	28±1.07	24±7.31	38±4.23	26±2.84
$\mathbf{A}_{26}$	3-F/$CH(CH_3)_2$	14±2.79	6±2.0	52±3.10	31±1.68	47±2.58	28±5.60
$\mathbf{A}_{27}$	3,4-diF/$CH(CH_3)_2$	28±1.51	13±3.30	48±1.38	28±4.46	60±3.52	34±2.73
$\mathbf{A}_{28}$	4-Cl/$CH(CH_3)_2$	36±4.90	23±0.24	63±2.25	37±2.26	58±6.40	29±4.84
$\mathbf{A}_{29}$	3-Cl/$CH(CH_3)_2$	27±4.8	17±0.17	58±5.38	42±3.78	46±0.81	12±1.27
$\mathbf{A}_{30}$	3,4-diCl/$CH(CH_3)_2$	56±1.50	27±1.13	52±5.18	45±1.31	66±2.39	39±5.16
$\mathbf{A}_{31}$	4-CF_3/$CH(CH_3)_2$	40±4.74	37±3.12	61±2.85	27±3.72	23±1.45	18±1.06
$\mathbf{A}_{32}$	3-CF_3/$CH(CH_3)_2$	48±2.57	37±3.08	49±2.50	26±3.07	52±2.79	31±0.97
$\mathbf{A}_{33}$	4-Br/$CH(CH_3)_2$	46±2.79	28±9.93	50±2.49	18±3.69	46±4.38	12±1.27
$\mathbf{A}_{34}$	3-Br/$CH(CH_3)_2$	39±1.80	34±2.56	53±4.20	43±7.87	25±3.42	11±1.20
$\mathbf{A}_{35}$	4-CH_3/$CH(CH_3)_2$	34±1.69	30±3.10	44±1.68	23±7.73	33±0.96	14±1.27
$\mathbf{A}_{36}$	H/$CH(CH_3)_2$	40±4.53	29±3.81	48±1.38	28±1.16	25±2.80	19±4.15
**Bismerthiazol**		87±1.34	67±1.03	100±4.84	72±5.21	95±7.50	77±3.29
**Thiodiazole copper**		76±8.61	51±4.31	86±3.64	59±4.79	74±1.72	52±5.87

Table S2. Antibacterial activity of compounds against Xoo, Xac and Xoc.

Compd.	*Xoo*			*Xac*			*Xoc*		
	EC_{50} (μg/mL)	regression equation	r2	EC_{50} (μg/mL)	regression equation	r2	EC_{50} (μg/mL)	regression equation	r2
**A_1**	51.27±2.41	y=3.96x-1.78	0.98	49.72±2.56	y=5.69x+1.56	0.99	81.99±2.61	y=2.81x-0.39	0.96
**A_3**	90.01±3.12	y=4.06x-2.95	0.98	193.78±2.34	y=2.27x-0.19	0.99	124.69±3.22	y=1.74x+1.55	0.96
**A_4**	61.46±2.12	y=2.62x+0.32	0.97	96.21±3.21	y=3.23x-1.43	0.96	66.85±2.76	y=3.45x-1.30	0.98
**A_6**	53.03±1.22	y=1.67x+2.13	0.98	130.92±2.10	y=2.18x+0.39	0.97	93.68±4.56	y=2.49x-0.09	0.95
**A_{11}**	164.83±4.34	y=1.61x+1.43	0.98	262.44±3.56	y=1.19x+2.11	0.95	390.68±5.31	y=1.57x+0.93	0.97
**A_{13}**	184.66±5.21	y=1.46x+1.67	0.99	225.27±3.44	y=1.29x+1.95	0.96	130.99±2.15	y=1.80x+1.19	0.95
**A_{25}**	326.28±6.76	y=1.67x+2.13	0.96	421.17±4.22	y=1.21x+1.83	0.98	254.43±3.19	y=1.64x+2.13	0.95
**Bismerthiazol**[b]	61.45±2.10	y = 2.25x+0.98	0.98	45.22±1.10	y = 2.99x+0.05	0.95	70.97±2.29	y=2.02x+1.26	0.95
**Thiodiazole copper**[b]	88.14±2.22	y = 1.82x+1.46	0.95	77.04±3.22	y = 1.83x+1.55	0.96	98.22±1.78	y=2.43x+0.16	0.97

[a] The statistic analysis was conducted by ANOVA method at the condition of equal variances assumed ($p > 0.05$) and equal variances not assumed ($p< 0.05$). [b] The commercial antibacterial agents bismerthiazol and thiodiazole-copper were used as positive control.
